# Metabarcoding using multiplexed markers increases species detection in complex zooplankton communities

**DOI:** 10.1111/eva.12694

**Published:** 2018-09-15

**Authors:** Guang K. Zhang, Frédéric J. J. Chain, Cathryn L. Abbott, Melania E. Cristescu

**Affiliations:** ^1^ Department of Biology McGill University Montreal Quebec Canada; ^2^ Pacific Biological Station, Fisheries and Oceans Canada Nanaimo British Columbia Canada; ^3^ Present address: Department of Biological Sciences University of Massachusetts Lowell One University Avenue Lowell MA

**Keywords:** 18S, cytochrome *c* oxidase subunit I, metabarcoding, multigene, multiple primer pairs, zooplankton

## Abstract

Metabarcoding combines DNA barcoding with high‐throughput sequencing, often using one genetic marker to understand complex and taxonomically diverse samples. However, species‐level identification depends heavily on the choice of marker and the selected primer pair, often with a trade‐off between successful species amplification and taxonomic resolution. We present a versatile metabarcoding protocol for biomonitoring that involves the use of two barcode markers (COI and 18S) and four primer pairs in a single high‐throughput sequencing run, via sample multiplexing. We validate the protocol using a series of 24 mock zooplanktonic communities incorporating various levels of genetic variation. With the use of a single marker and single primer pair, the highest species recovery was 77%. With all three COI fragments, we detected 62%–83% of species across the mock communities, while the use of the 18S fragment alone resulted in the detection of 73%–75% of species. The species detection level was significantly improved to 89%–93% when both markers were used. Furthermore, multiplexing did not have a negative impact on the proportion of reads assigned to each species and the total number of species detected was similar to when markers were sequenced alone. Overall, our metabarcoding approach utilizing two barcode markers and multiple primer pairs per barcode improved species detection rates over a single marker/primer pair by 14% to 35%, making it an attractive and relatively cost‐effective method for biomonitoring natural zooplankton communities. We strongly recommend combining evolutionary independent markers and, when necessary, multiple primer pairs per marker to increase species detection (i.e., reduce false negatives) in metabarcoding studies.

## 1 INTRODUCTION

Molecular methods of species identification are generating new opportunities for surveying biodiversity by overcoming important limitations of traditional taxonomy, which is laborious and invasive and requires advanced taxonomic expertise (Creer et al., 2016; Cristescu, 2014). One of the most promising methods, often referred as metabarcoding (Taberlet, Coissac, Hajibabaei, & Rieseberg, 2012), combines the DNA barcoding approach, which was originally developed to identify single specimens (Hebert, Cywinska, Ball, & deWaard, 2003; Ratnasingham & Hebert, 2007), with high‐throughput sequencing (HTS) technologies to reveal species composition in “bulk” samples or environmental DNA (eDNA) samples (i.e., DNA that leaks into the environment; reviewed in Taberlet et al., 2012). Although metabarcoding is a very promising method, its efficient application is still hindered by several technical limitations which are often responsible for generating both false negatives (species being present in a sample but not detected) and false positives (species being detected but not present). This method relies on well‐designed primers to amplify a homologous marker gene from a taxonomically complex sample (Creer et al., 2016). Thus, challenges often include finding a suitable DNA region to amplify across target taxa, dealing with PCR amplification errors and sequencing artifacts, developing high‐quality reference sequence databases, and choosing the appropriate bioinformatic steps to accommodate variable sequence divergence thresholds among species (Cristescu, 2014; Taberlet et al., 2012; Yoccoz, 2012). Choosing one or more appropriate genetic markers for metabarcoding is considered essential for accurate molecular species identification (Bucklin, Lindeque, Rodriguez‐Ezpeleta, Albaina, & Lehtiniemi, 2016; Clarke, Beard, Swadling, & Deagle, 2017), as it affects both PCR amplification success and species‐level resolution.

To allow efficient species identification, the genetic marker used must show high interspecific variation and low intraspecific variation. However, it is often difficult to strike a balance between high amplification success across diverse taxon groups and species‐level resolution (Bohle & Gabaldón, 2012). Markers that undergo fast rates of evolution have discriminative taxonomic power for resolving closely related species but often lack conserved primer binding sites appropriate for amplifying broad taxonomic groups. Degenerate primers are often designed when conserved primer binding sites are not available. However, primer‐template mismatches can generate imperfect primer match with some DNA templates (Pinol, Mir, Gomez‐Polo, & Agust, 2015). This primer bias often distorts the biotic composition.

Most current metabarcoding projects use a single locus approach, and the most common markers are the cytochrome *c* oxidase subunit I (COI) for animals (Hebert et al., 2003; Leray et al., 2013), internal transcribed spacer (ITS) for fungi (Horton & Bruns, 2001; Schmidt et al., 2013), and plastid DNA (*mat*K and *rbc*L) for land plants (Chase & Fay, 2009; Yoccoz, 2012). Alternative single markers are standardly used for particular taxa. For example, 12S is the most commonly used metabarcoding marker for fish (Miya et al., 2015; Valentini et al., 2016). Using a single organelle marker can occasionally cause erroneous species identification due to interspecific mitochondrial introgressions (Funk & Omland, 2003; Meyer & Paulay, 2005); therefore, the use of both uniparentally inherited organelle DNA and biparentally inherited DNA has been recommended (Taberlet et al., 2012). The mitochondrial COI gene has high resolution for species identification and relatively extensive reference sequence libraries (Ratnasingham & Hebert, 2007), but it is often difficult to amplify consistently across diverse taxonomic groups due to lack of conserved primer binding sites (Deagle, Jarman, Coissac, Pompanon, & Taberlet, 2014). It was suggested that using well‐designed degenerated COI primers could reduce the COI primer bias (Elbrecht & Leese, 2017). An alternative approach, tested here, is the use of multiple primers pairs per markers. In contrast to the mitochondrial COI gene, the nuclear 18S gene provides more conserved priming sites for greater amplification success across broad taxonomic groups, but often provides lower resolution for species identification (Bucklin et al., 2016; Hebert et al., 2003; Saccone, Giorgi, Gissi, Pesole, & Reyes, 1999). Another major disadvantage with using 18S as a metabarcoding marker is that it varies in length at V4 region across diverse species, causing sequence alignment uncertainties across broad taxa and consequently difficulties estimating divergence thresholds and implementing clustering approaches for species identification (Flynn, Brown, Chain, MacIsaac, & Cristescu, 2015; Hebert et al., 2003).

These marker‐related problems led many researchers to propose the need to use multiple markers in metabarcoding studies (Bucklin et al., 2016; Chase & Fay, 2009; Drummond et al., 2015). The multimarker approach has the potential to reduce rates of false negatives and false positives. Despite these promises, a multigene approach has rarely been applied in metabarcoding studies, and comparisons of biodiversity estimates across the different markers are usually not reported (e.g., COI for metazoan and RuBiscCO for diatoms, Zaiko et al., 2015; species‐specific primer pairs of COI and cytochrome *b* markers, Thomsen et al., 2012; chloroplast *trn*L and *rbc*L for surveying different terrestrial habitats, Yoccoz, 2012). In addition, many multimarker metabarcoding studies use a single primer pair per marker (Clarke et al., 2017; Drummond et al., 2015; Kermarrec et al., 2013). Using multiple primer pairs is expected to reduce amplification biases and increase the opportunities of detecting all targeted taxonomic groups. To fully understand the performance of a multigene metabarcoding approach, mock communities are ideal because the expected number of species is known a priori (Clarke, Soubrier, Weyrich, Weyrich, & Cooper, 2014; Elbrecht et al., 2016; Kermarrec et al., 2013). Nonetheless, there are few studies that have taken this approach (but see Clarke et al., 2014). As such, there is an urgent need for experiments that test species detection rates and taxonomic identification accuracy in mock communities using multimarker and multiprimer pair metabarcoding to test the validity of this method for biomonitoring.

In this study, we use a combination of mitochondrial (COI) and nuclear markers (18S) and multiple COI primer pairs in a single Illumina run for recovering species by metabarcoding mock communities of zooplankton. Species detection is assessed among markers and primer pairs to evaluate the benefit of multimarker and multiprimer pairs per marker. We also compare species detection rates and detection accuracies with a single‐marker metabarcoding experiment in which 18S was used alone. We calibrate the multiplexing multigene approach using a series of mock communities containing single individuals per species (SIS), multiple individuals per species (MIS), and populations of single species (PSS). The resulting calibrated workflow performs better than a single marker or single primer pair approach and can be applied to assess zooplankton biodiversity in natural aquatic habitats.

## 2 MATERIALS AND METHODS

### 2.1 Primer testing

**Figure 1:**
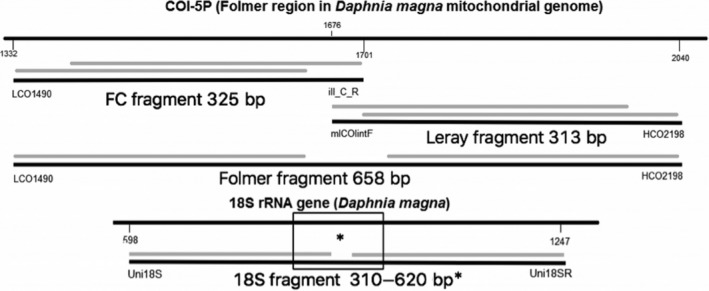
The amplified fragments used for metabarcoding. The 5′ end fragment of 325 bp refers to the FC fragment matching the COI‐5P gene before the nucleotide position 400. The 3′ end fragment of 313 bp refers to the Leray fragment matching the COI‐5P gene after nucleotide position 300, and the whole COI‐5P gene of 658 bp refers to the Folmer fragment with forward reads R1 matching before nucleotide position 300 and the reverse reads R2 matching after nucleotide position 400. The primers are not included in the fragment lengths, and the gray lines refer to the forward and reverse reads from the paired‐end 300 bp Illumina MiSeq next‐generation sequencing. *The 18S fragment sizes vary between species, resulting in some forward and reverse reads that do not overlap

Preliminary primer amplification tests were conducted qualitatively on a total of 103 species using 13 COI primer pairs (COI‐5P region) and one 18S primer pair (V4 region; see Supporting information Table S1 for the complete list of primers). We selected primer pairs known to provide amplification success across a wide range of taxa as well as good discriminatory power for species identification. The only 18S primer pair tested is known for its successful amplification across a broad range of zooplankton groups (Brown, Chain, Zhan, MacIsaac, & Cristescu, 2016; Chain, Brown, MacIsaac, & Cristescu, 2016; Zhan et al., 2013). Specimens used for primer testing were sampled from 16 major Canadian ports across four geographic regions (Atlantic coast, Pacific coast, Arctic, Great lakes; Chain et al., 2016; Brown et al., 2016) and were identified morphologically by taxonomists. A total of 103 species belonging to the phyla Rotifera, Crustacea, Mollusca, and the Subphylum Tunicata were selected and tested (see Supporting information Table S2). A subset of those species was used to assemble mock communities for metabarcoding validation (see Supporting information Table S2). PCR amplification was performed in a total volume of 12.5 μl: 0.2 μM of each forward and reverse primers, 1.25U *taq* DNA polymerase (GeneScript, VWR), 2 mM Mg^2+^, 0.2 μM dNTP, and 2 μl of genomic DNA. The PCR conditions of each primer pair were based on their sources in the literature (Supporting information Table S1). After the broad primer testing, four primer pairs were selected for metabarcoding several mock communities: one targeting the nuclear 18S V4 region (Zhan et al., 2013) and three COI primer pairs producing three different (partially overlapping) fragments within the COI‐5P region (Figure 1, Supporting information Figure S1, Table 1).

**Table 1:** The four primer pairs used in this metabarcoding study: 18S primer pair amplifying the V4 region and three COI primer pairs amplifying different fragments of the COI‐5P gene. See Supporting information Table S1 for the complete list of primers used for the preliminary primer testing step

Fragment	Primer name	Sequence (5′–3′)	Direction	Target taxa	Reference	Fragment size
18S	Uni18S	AGGGCAAKYCTGGTGCCAGC	F	Metazoan	Zhan et al. (2013)	310–620^a^
	Uni18SR	GRCGGTATCTRATCGYCTT	R	Metazoan	Zhan et al. (2013)	
COI_FC	LCO1490	GGTCAACAAATCATAAAGATATTGG	F	Various phyla	Folmer et al. (1994)	325
	Ill_C_R	GGIGGRTAIACIGTTCAICC	R	Arthropoda	Shokralla et al. (2015)	
COI_Leray	mlCOIintF	GGWACWGGWTGAACWGTWTAYCCYCC	F	Various phyla	Leray et al. (2013)	313
	HCO2198	TAAACTTCAGGGTGACCAAAAAATCA	R	Various phyla	Folmer et al. (1994)	
COI_Folmer	LCO1490	GGTCAACAAATCATAAAGATATTGG	F	Various phyla	Folmer et al. (1994)	658
	HCO2198	TAAACTTCAGGGTGACCAAAAAATCA	R	Various phyla	Folmer et al. (1994)	

Note

a The 18S fragment varies in length for different species.

### 2.2 Assemblage of mock communities

Mock communities were constructed with the aim of incorporating various levels of genetic variation (intragenomic, intraspecific, interspecific) and representing natural zooplankton communities including species from broad taxonomic groups: Mollusca, Rotifera, Tunicata, and Crustacea (Amphipoda, Anostraca, Cladocera, Cirripedia, Copepoda, Decapoda) (see Supporting information Table S3 for species list). Morphologically identified specimens are at the species or genus level, with a few exceptions that were identified only to the family level. DNA was extracted from each specimen using Qiagen DNeasy Blood & Tissue kits and stored in ultrapure water in the freezer at −20°C as described in Brown, Chain, Crease, MacIsaac, and Cristescu (2015). The DNA was eventually combined into several different mock community assemblies. Laboratory blanks were used consistently during DNA extractions to assure no interference from contamination.

**Figure 2:**
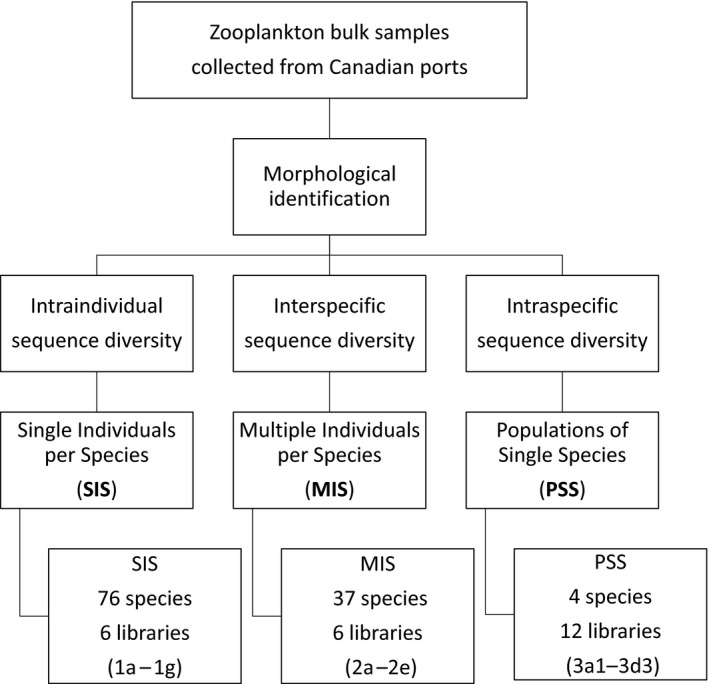
Flowchart of experimental design. See Supporting information Table S9 for a detailed assemblage of the three main mock communities and the libraries that constitute each community

Three types of mock communities were assembled (Figure 2), hereafter referred to as single individuals per species (SIS) consisting of single individuals from each of 76 species (Supporting information Table S3: 1a–e, g), multiple individuals per species (MIS) consisting of various numbers of individuals from 37 species (Supporting information Table S3: 2a–e, g), and populations of single species (PSS) consisting of single, low, and high number of individuals of single species (Supporting information Table S3: 3a1–d3), respectively. The inclusion of single individuals in the SIS communities allowed examination of species detection with only interspecific variation. The MIS communities, which most closely resembled natural communities, allowed the examination of species detection with both intraspecific and interspecific variation. The PSS communities allowed the examination of intraspecific variation and the relationship between read abundance and the number of individuals of the same species.

### 2.3 Library preparation and next‐generation sequencing (NGS)

DNA extractions were first quantified using PicoGreen (Quant‐iT^™^ Picogreen dsDNA Assay Kit, Thermo Fisher Scientific Inc.), then diluted to 5 ng/μl. The protocol “16S Metagenomic Sequencing Library Preparation” (Illumina Inc.) was used with small modifications to prepare the sequencing‐ready libraries. Library preparation involved a first PCR, followed by a first cleaning with Agencourt AMPure beads (Beckman Coulter Life Sciences Inc.), a second PCR with Nextera Index kit (V3), and a second clean‐up prior to next‐generation sequencing (NGS). The first PCR was conducted in two replicates for each library and each of the four DNA fragments. Negative controls were included in each round of PCRs. PCR amplification was performed in a total volume of 12.5 μl: 0.2 μM of each forward and reverse primers, 6 μl of 2xKAPA HiFi HotStart ReadyMix (KAPA Biosystems Inc., USA), and 1.5 μl of diluted genomic DNA. Due to the incompatibility of KAPA kit with primers involving inosine (“I”) in the COI primer Ill_C_R (Shokralla et al., 2015), all the FC fragments were amplified using a standard PCR gradient with taq DNA polymerase (GeneScript, VWR) as in the original paper (Shokralla et al., 2015). The PCR thermocycler regimes were the same as in the original papers: 18S V4 (Zhan et al., 2013), FC (Shokralla et al., 2015), Leray (Leray et al., 2013), and Folmer (Folmer, Black, Hoeh, Lutz, & Vrijenhoek, 1994) (see Figure 1 for details). The two replicates of each PCR reaction for each fragment were pooled together after visualization on a 1% electrophoresis gel. The PCR products were quantified and pooled (equal volumes from each fragment) so that each library contained all four fragments. After this step, there was a total of 24 libraries each with four different PCR amplicons (Supporting information Table S3): six SIS libraries (simple communities); six MIS libraries (complex communities); and 12 PPS libraries (single species communities). The 24 libraries obtained were cleaned using ultrapure beads at ratio of 0.875 (28 μl beads in 32 μl solution), indexed using Nextera^®^ XT Index Kit (24‐index, V3), and final clean‐up using ultrapure beads to become sequencing‐ready. All libraries were submitted to Genome Quebec for final quantification, normalization, and pooling and were sequenced using pair‐end 300‐bp reads on an Illumina MiSeq sequencer in one run. Note that the four single individuals per species (SIS) libraries (1a, 1b, 1c, 1d) and the four multiple individuals per species (MIS) libraries (2a, 2b, 2c, 2d) were quantified and pooled in equal molar for next‐generation sequencing. The PSS libraries (3a1–d3) were quantified and pooled in different molars relative to their number of individuals of the species. It is worth nothing that the pooling of PCR amplicons prior to indexing makes this a more cost‐effective approach than methods that separately index each PCR amplicon prior to pooling.

The same genomic DNA of the four SIS (libraries 1a, 1b, 1c, 1d) and the four MIS (libraries 2a, 2b, 2c, 2d) communities was sequenced using only the 18S primers in a separate run. The library preparation and sequencing was performed in the same proportions of 5% of one flowcell using the same Illumina MiSeq pair‐end 300‐bp platform. This experiment was conducted to compare sequencing depth and species detection rates between a metabarcoding run with a single marker versus a multiplexed metabarcoding run with other markers/fragments (more than one marker/fragment per run for the same sample/library).

### 2.4 Building a local reference database

We created a local database composed of 149 total sequences used for taxonomic assignment of reads (see Supporting information Table S4). Reference sequences were either generated by Sanger sequencing in this study (23 sequences), acquired from related projects conducted at the Biodiversity Institute of Ontario (BIO) or in our laboratory on the same zooplankton populations (18 sequences), or obtained from online databases (108 reference sequences; NCBI GenBank http://www.ncbi.nlm.nih.gov/nuccore, BOLD http://www.boldsystems.org/). Congeneric or confamilial species were used as reference sequences when the focal species were identified to family level only, or when no online reference sequences where available and we had insufficient DNA remaining for Sanger sequencing (Supporting information Table S4). All COI reference sequences were aligned and adjusted to have an equal length of 652 bp, so the FC fragments matched the 5′ end of the reference sequences, the Leray fragments matched the 3′ end of the reference sequences, and the Folmer fragments matched the whole COI‐5P gene region (see Figure 1 for the detailed fragments positions). The 18S reference sequences contained the V4 region without trimming. The best BLAST hit against our local reference database was used to classify each sequence read with a minimum of 95% identity and an alignment length of at least 150 bp in forward and reverse reads. These relatively relaxed thresholds were used to accommodate the species with congeneric or confamiliar reference sequences. In the case of multiple best hits, if the correct species assignments could not be confirmed manually based on reads blasting against the online database on GenBank, they were excluded from further analysis.

### 2.5 Bioinformatics analyses

The bioinformatic pipeline in this study consisted of demultiplexing, quality filtering, trimming raw reads, and assigning taxonomy via BLASTN (Altschul, Gish, Miller, Myers, & Lipman, 1990) against our local reference database. Taxonomic assignment was conducted at a minimum of 95% identity. We performed first a local BLAST to find unique best hits. When multiple species had equal identity, a second BLAST search in GenBank was performed to find unique best hits. If multiple species still appeared as having equal identity, the read was excluded from further analysis (Supporting information Figure S2).

Each mock community was processed as a separate “library” and could be demultiplexed via their unique combination of indices. Raw reads were assigned to their corresponding libraries, generating paired forward R1 and reverse R2 files for each library (Raw read pairs in Table 2). The raw reads were then quality filtered and trimmed via “Quality Trimmer” from the FASTX‐Toolkit (http://hannonlab.cshl.edu/fastx_toolkit/), with a minimum Phred quality score of 20 and a minimum length of 150 bp after trimming (see trimmed‐R1 and trimmed‐R2 in Table 2). After quality trimming, the R1 and R2 reads were separately used as queries in BLAST against the local database. The BLAST results of R1 and R2 were concatenated together (see paired reads after trimming in Table 2), and only the sequences with both R1 and R2 returning an accepted BLAST match to a reference sequence (>95% identity and >150 bp) were kept for further analysis (see filter‐blasting step in Table 2). The BLAST results were then further filtered based on whether both R1 and R2 reads were assigned to the same species (see filter‐blasting same species in Table 2 and Supporting information Figure S2).

**Table 2:** Reads remaining after each bioinformatic filtering step: raw read pairs in each library; R1 and R2 after trimming by quality score (Phred ≥ 20) and length (150 bp); paired reads after performing BLAST searches where each read (R1 and R2) match a reference sequence (>95% identity and >150 bp length); paired reads where both R1 and R2 match the same reference sequence; paired reads assigned to each fragment (18S, FC, Leray, Folmer) based on the read position alignment (Figure 2) on the reference sequence, as reported in the BLAST results

Library	Raw read pairs	Trimmed‐R1	Trimmed‐R2	BLAST matches	BLAST matches identical	18S	FC	Leray	Folmer
1a	786,961	732,060	613,404	324,738	207,556	39,923	4,062	151,626	11,945
1b	854,975	815,425	773,465	472,101	407,426	92,011	55,333	238,036	22,046
1c	929,743	902,841	875,974	584,322	503,691	166,998	73,084	231,414	32,195
1d	884,801	853,816	820,011	338,938	215,921	123,502	15,556	71,483	5,380
1e	914,893	887,362	861,284	533,725	443,829	153,957	61,232	187,120	41,520
1 g	781,706	751,895	725,703	462,968	380,403	103,268	49,898	212,793	14,444
2a	925,818	896,816	879,586	679,961	637,883	216,345	81,245	306,006	34,287
2b	1,036,566	1,009,121	963,326	504,939	454,293	163,410	91,998	177,804	21,081
2c	1,619,499	1,529,135	1,444,012	549,010	321,592	97,184	123,404	14,002	87,002
2d	760,043	738,317	718,755	574,756	383,888	76,266	55,724	220,464	31,434
2e	915,977	890,991	872,002	665,676	550,787	161,688	67,174	272,534	49,391
2 g	933,645	891,848	862,069	464,301	405,228	84,980	58,609	242,543	19,096
3a1	864,551	808,857	758,339	460,992	451,683	106,024	16,847	309,430	19,382
3a2	620,639	599,186	562,566	344,398	343,891	165,357	14,056	154,023	10,455
3a3	848,115	807,089	749,840	487,477	486,745	207,189	23,719	237,827	18,010
3b1	665,830	646,399	618,519	391,294	370,523	49,843	87,122	228,910	4,648
3b2	598,325	582,154	560,215	360,447	344,473	45,137	80,649	216,754	1,933
3b3	604,169	551,276	516,445	254,613	245,801	39,495	7,421	198,880	5
3c1	878,572	837,934	796,782	639,100	453,527	71,604	37,155	325,269	19,499
3c2	984,226	945,575	896,894	734,085	514,066	87,262	56,390	336,935	33,479
3c3	947,283	906,549	856,936	708,977	509,943	75,427	61,220	348,371	24,925
3d1	845,249	734,376	670,594	494,308	409,633	52	104,521	295,096	9,964
3d2	826,818	773,111	732,549	594,312	486,103	266	167,564	301,917	16,356
3d3	702,450	615,365	563,521	418,385	323,118	33	139,858	164,883	18,344
Average	863,786	821,146	778,866	501,826	410,500	96,968	63,910	226,838	22,784
STD	200,992	194,408	189,389	129,699	104,787	62,575	41,850	81,269	18,427

The comparison of species detection among the four fragments in the SIS and MIS communities was performed on 55 of 78 species, due to 13 species having been identified only to the family or genus levels, seven species with no available 18S V4 reference sequences, and three species with more than 100 bp missing of their 18S V4 reference sequences. Species detection was confirmed when one or more read(s) matched a reference sequence with >95% identity and >150 bp. The custom python and bash script can be viewed in Appendix A1.

## 3 RESULTS

### 3.1 Primer testing

The preliminary amplification success of 14 primer pairs was tested on 103 species (Supporting information Table S2). The highest success of 76% was observed for the 18S fragment (Zhan et al., 2013), followed by the COI_Radulovici fragment (58%) (Radulovici, Bernard, & Dufresne, 2009) and then the COI_FC fragment (50%) (Shokralla et al., 2015). The overall amplification success rate of the COI_Leray fragment (38.5%) (Leray et al., 2013) was similar to the COI_Folmer fragment (37.5%) (Folmer et al., 1994). Although the three COI fragments COI_FC, COI_Leray, and COI_Folmer were designed to target a wide range of phyla, amplification success was found to be dependent on the species group. When selecting the primer for the metabarcoding study, we considered not only the overall performance of the primers under our specific conditions but also the size of the amplicons, as well as the general use of the primer pair in other barcoding‐related studies.

### 3.2 Read abundance comparison

**Figure 3:**
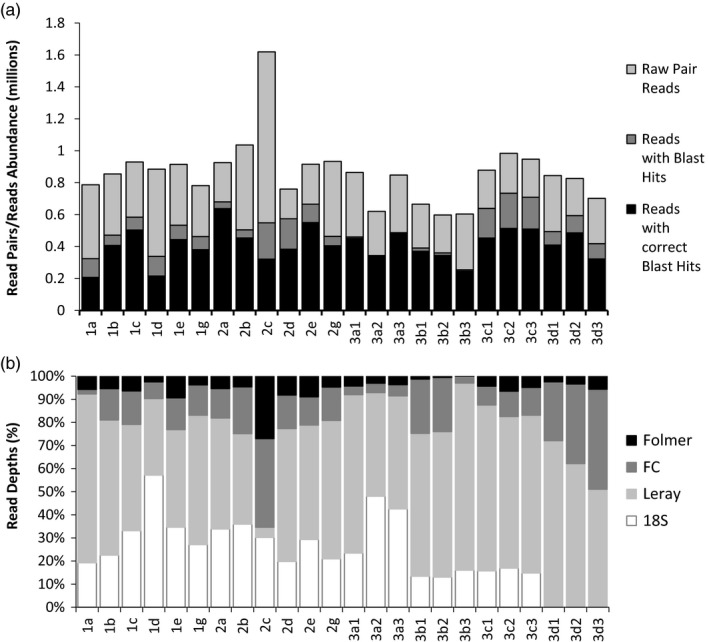
Read abundance across the 24 libraries. (a) The total number of reads retrieved before and after filtering represented in stacking columns. (b) The percentages of reads from each of the four fragments shown in the 100% stacking columns. The 24 libraries are as follows: 1a–g: single individuals per species (SIS); 2a–g: multiple individuals per species (MIS); 3a1–d3: populations of single species (PSS)

A total of 20.73 million raw read pairs were sequenced from the mock communities, of which 16.72 million paired reads remained after quality filtering (Table 2). After performing BLAST searches, 12.04 million paired reads remained with taxonomic assignments (Figure 3a). The number of raw read pairs varied in the four single individuals per species (SIS) libraries (1a, 1b, 1c, 1d) and the four multiple individuals per species (MIS) libraries (2a, 2b, 2c, 2d), especially 2c, despite that libraries were quantified and pooled in equal molar before sequencing (Figure 3a). Based on the BLAST results, the forward R1 and reverse R2 reads could generally be assigned to the same species but not always (see Figure 3a). The relative percentages of read abundance of the four fragments differed across the 24 libraries: The most abundant fragment overall was the Leray fragment (average read abundance of 0.227 million), followed by the 18S fragment (0.097 million), then the FC fragment (0.064 million), and the Folmer fragment with the lowest abundance of 0.023 million reads (Figure 3b). No significant correlation was observed at the intraspecific level between the number of individuals and the read abundance in the PSS libraries.

### 3.3 The performance of the 18S marker when used alone vs. with other markers

The method tested here is a multimarker approach with more than one marker sequenced in one run rather than requiring multiple runs, making it versatile and cost‐effective. However, the impact of this “multiplex” approach on species detection rates and sequencing depth (number of reads per individual/species) needs to be examined. We compared results from the 18S marker in our multiplexed multimarker approach to those in a single marker approach using both the SIS communities (Supporting information Table S5) and MIS communities (Table 3). In both cases, the sequencing depth (number of reads) on average and per individual or per species was consistent between the single‐marker and multimarker datasets (Table 3 and Supporting information Table S5). In the SIS communities of 56 species, discrepancies were only found when read counts were very low. For example, three species were detected exclusively in the single‐marker dataset, while three species were detected exclusively in the multimarker datasets, but the number of reads in all six of these instances was low (≤11 reads), representing about 0.003% of the total taxonomically‐assigned reads. In the MIS communities of 14 species, only two species had different detection between the single‐marker and multimarker datasets: *Leptodora kindtii* was only detected in the single‐marker experiment with 47 reads, and *Corbicula fluminea* was only detected in the multimarker datasets with nine reads (see Table 3). This demonstrates that the majority of species were consistently detected in both single‐marker and multimarker metabarcoding approaches (50 of 56 species in SIS and 12 of 14 species in MIS). Furthermore, the sequencing depth per individual and per species (between single‐marker and multimarker approach) was highly positively correlated (SIS: Pearson's correlation coefficient *r* = 0.965, *R* ^2^ = 0.931; MIS: Pearson's *r* = 0.873, *R* ^2^ = 0.763).

**Table 3:** Read count comparison for the 18S V4 marker when used alone vs. when multiplexed with three COI markers/fragments to sequence the multiple individuals per species (MIS) libraries (2a, 2b, 2c, 2d)

Phylum (subphylum)	Order	Family	Genus/family	Species	*n*	18S marker alone	18S marker multiplexed with other markers/fragments
Crustacea	Amphipoda	Gammaridae	*Gammarus*	*Gammarus lawrencianus*	1	0	0
Crustacea	Amphipoda	Hyalellidae	*Hyalella*	*Hyalella* clade 8	5	31239	58960
Crustacea	Anostraca	Artemiidae	*Artemia*	*Artemia spp*	2	74529	82148
Crustacea	Calanoida	Acartiidae	*Acartia*	*Acartia longiremis*	5	0	0
Crustacea	Calanoida	Temoridae	*Eurytemora*	*Eurytemora affinis*	23	78877	75980
Crustacea	Calanoida	Diaptomidae	*Leptodiaptomus*	*Leptodiaptomus minutus*	1	9180	10281
Crustacea	Decapoda	Portunidae	*Carcinus*	*Carcinus maenas*	2	4538	168
Crustacea	Decapoda	Palaemonidae	*Palaemonetes*	*Palaemonetes spp*	5	138730	120748
Crustacea	Diplostraca	Daphniidae	*Daphnia*	*Daphnia pulex*	10	25065	65503
Crustacea	Diplostraca	Leptodoridae	*Leptodora*	*Leptodora kindtii*	5	47*	0*
Crustacea	Sessilia	Balanidae	*Balanus*	*Balanus crenatus*	10	150068	77705
Crustacea	Sessilia	Chthamalinae	*Chthamalus*	*Chthamalus dalli*	1	13758	22117
Mollusca	Cycloneritimorpha	Neritidae	*Nerita*	*Nerita spp*	1	7745	31272
Mollusca	Venerida	Cyrenidae	*Corbicula*	*Corbicula fluminea*	5	0*	9*
Average number of reads per individual (*n* = 76)						7023	7170
Average number of reads per species (*n* = 14)						38127	38921

The “*n*” refers to the number of individuals per species. Two instances when species detection differed between the 18S marker alone and 18S marker multiplexed are marked as an asterisk. Pearson's correlation coefficient *r* = 0.873, *R* ^2^ = 0.763.

### 3.4 Primer pair choice and species detection

**Figure 4:**
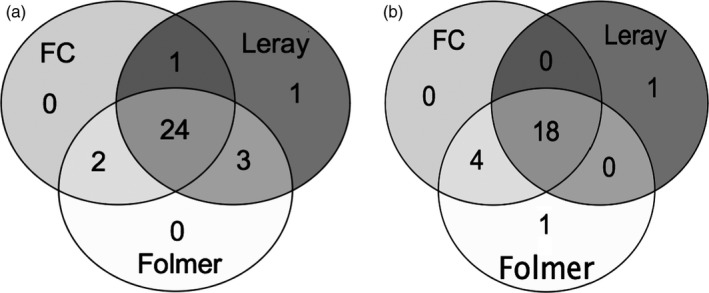
Comparison of three COI fragments (FC, Leray, Folmer) on species detection in (a) single individuals per species (SIS) and (b) multiple individuals per species (MIS)

The three different COI fragments selected correspond to the COI‐5P region (Figure 1), encompass regions with different levels of genetic variation across the species included in the mock communities, and show variation in the amplification success of various taxonomic groups. The number of species detected among the three COI fragments was compared in both SIS communities and MIS communities (Figure 4). We found that few species (2%–3%) were uniquely recovered by a single COI fragment, and most of the species (45%–60%) were consistently detected by all three COI fragments. The three COI fragments together detected 59% of the species in SIS and 80% of the species in MIS communities (Figure 4). The use of three COI primer pairs improved species detection rates by 3.8%–7.5% in SIS and 3%–17% in MIS communities compared to using a single COI primer pair.

### 3.5 Marker choice and species detection

**Figure 5:**
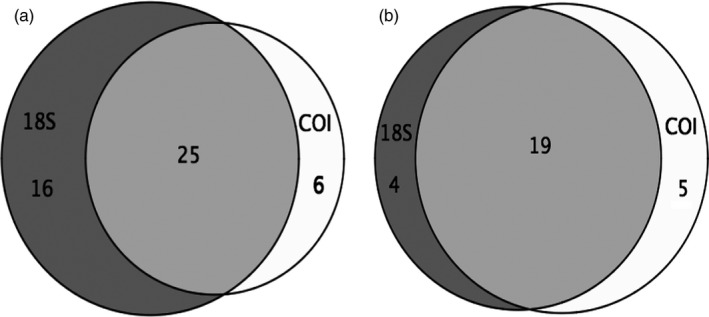
Comparison of COI vs. 18S markers on species detection among mock communities: (a) single individuals per species (SIS) and (b) multiple individuals per species (MIS)

**Figure 6:**
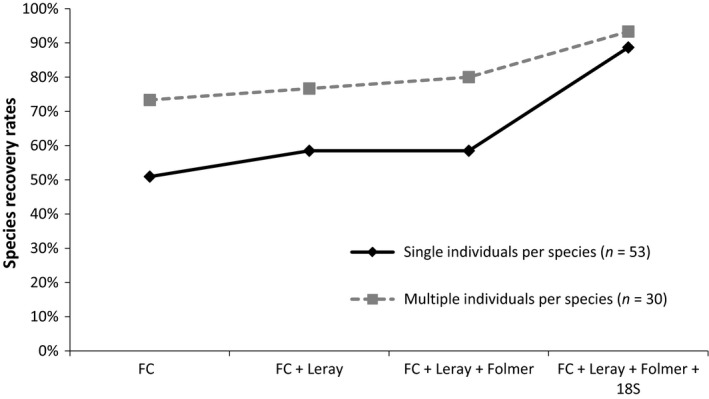
Accumulation of species recovery percentages after including different fragments in both single individuals per species (SIS) and multiple individuals per species (MIS). The first data points show the percentage of species detected by the FC fragment alone and then species detected by both FC and Leray fragments, followed by adding the Folmer and 18S fragments subsequently

Species detection rates in the SIS communities and MIS communities were also compared between the 18S V4 marker and the three COI fragments considered together (Figure 5). The 18S marker detected 18.9% more of the species compared to the combination of the three COI markers in the SIS community, but 3.3% fewer species in the MIS communities. The two markers generally recovered about half of the same species (47.2%–63.3%). Species recovery was significantly improved by adding both markers (18S + COI) in both SIS and MIS communities (Figure 6), improving species detection rates by 11.3%–16.6% compared to using the 18S marker alone and by 13.3%–30.2% compared to using the three fragments of the COI marker (see Supporting information Table S6 for detailed species detection).

### 3.6 False positives: detection of species not intentionally included in mock communities

The incidence of false positives (species detected but not intentionally included) in the three main communities were compared between the 18S V4 marker and the three COI fragments (Table 4). In general, a low number of reads (sometime single reads) were assigned as contaminants (Supporting information Tables S7 and S8). For SIS libraries, the 18S marker had the lowest average contamination rates (3.36%), while the COI_Folmer marker had the highest average contamination rates (38.9%). For MIS libraries, all four fragments had relatively low contamination rates. For PSS libraries, the 18S marker had high contamination rates in 3d1–3d3 libraries. These libraries were composed of multiple individuals of the species *Leptodora kindtii,* a voracious predator with potentially diverse gut content. The three COI fragments had high contamination rates in libraries 3a1 and 3c2, and the Folmer fragment had the highest contamination rates in libraries 3b3 and 3d1. Overall, false positives are relatively low in SIS and MIS communities. In the PSS libraries, rates of false positives vary greatly depending on species and fragments in the PSS libraries.

**Table 4:** Rates of false negatives (species not detected) and false positives (species detected but not included in the mock communities) for the four fragments in all libraries

Library	False negatives %	False positives (contamination) %			
		18S	FC	Leray	Folmer
1a	40.00	0.43	1.90	0.15	0.16
1b	31.25	4.90	0.11	0.34	0.05
1c	28.57	6.09	36.24	94.16	9.91
1d	25.00	4.96	16.12	80.79	93.51
1e	28.57	3.59	32.18	57.95	7.38
1 g	7.41	0.22	0.06	0.02	0.06
SIS Average	26.80	3.36	14.43	38.90	18.51
2a	0.00	3.52	1.61	0.05	0.54
2b	20.00	0.15	0.09	0.13	0.11
2c	0.00	0.24	0.21	1.60	0.02
2d	0.00	0.40	0.20	0.11	0.07
2e	7.14	1.89	0.73	0.01	0.21
2 g	11.54	0.34	0.03	0.02	0.05
MIS Average	6.45	1.09	0.48	0.32	0.17
3a1	0.00	0.07	14.96	51.59	16.62
3a2	0.00	0.01	0.18	0.09	0.02
3a3	0.00	0.01	0.17	0.07	0.02
3b1	0.00	0.12	0.02	0.05	0.09
3b2	0.00	6.95	9.01	0.35	0.52
3b3	0.00	0.23	0.12	0.04	60.00
3c1	0.00	0.48	2.02	2.13	0.41
3c2	0.00	0.41	36.36	45.27	8.61
3c3	0.00	0.55	0.41	0.81	0.21
3d1	0.00	80.77	0.04	0.08	100.00
3d2	0.00	96.99	0.01	0.09	0.05
3d3	0.00	75.76	0.02	0.12	0.04
PPS Average	0.00	21.86	5.28	8.39	15.55

## 4 DISCUSSION

Marker choice has been the focus of much discussion in many metabarcoding studies because all markers have some advantages and disadvantages (Deagle et al., 2014). Both the hypervariable 18S V4 region and the COI‐5P region have previously been used for assessing aquatic biodiversity in single marker metabarcoding studies (Aylagas, Borja, Irigoien, & Rodriguez‐Ezpeleta, 2016; Brown et al., 2016; Chain et al., 2016; Leray et al., 2013; Zhan et al., 2013). The use of multiple group‐specific COI primer pairs has been suggested as an efficient method for obtaining higher amplification success when studying broad taxonomic groups (Bucklin et al., 2016; Cristescu, 2014). Moreover, the use of both uniparentally inherited markers such as COI and biparentally inherited markers such as 18S has been suggested as an efficient method for increasing the accuracy of species identification (Taberlet et al., 2012). Through the use of mock communities with known taxonomic composition, we demonstrate that a multigene (COI and 18S) and multiprimer pair (three COI primer pairs) metabarcoding approach can improve species detection and provides the built‐in ability to cross‐validate results.

### 4.1 Multiple primer pairs

The mitochondrial COI marker has been reported to be technically challenging for amplification of broad taxonomic groups due to the lack of conserved priming sites (Bucklin et al., 2016; Deagle et al., 2014). Both group‐specific (Bucklin et al., 2010) and species‐specific (Thomsen et al., 2012) primer pairs have been used in COI barcoding and metabarcoding. The 18S primer pair used in this study targets the V4 region of zooplankton and was successful in previous metabarcoding studies (Brown et al., 2015, 2016; Chain et al., 2016; Zhan et al., 2013). The 13 COI primer pairs tested here showed major differences in overall amplification success depending on the group of species. Overall, amplification success of the 13 COI primer pairs followed species‐specific rather than group‐specific patterns in the majority of taxa tested here (Supporting information Table S2). In addition to amplification success across taxa of interest, amplicon length is also an important consideration for studies using degraded environmental DNA, which require short amplicons (Meusnier et al., 2008), and is upwardly limited by the capacity of NGS technology to obtain accurate long reads (Shaw, Weyrich, & Cooper, 2017). For example, primer pairs used here that amplified more than 600 bp (Tables S1 and S2) had sequence gaps between the forward and reverse reads when sequenced on the Illumina MiSeq pair‐end 300‐bp platform. Therefore, the combination of the full COI fragment of 658 bp (COI_Folmer) with overlapping two short COI fragments (COI_FC and COI_Leray) of 325 bp and 313 bp was chosen for metabarcoding our mock communities with the expectation of generating higher species amplification success and suitability for studying natural community DNA or degraded eDNA.

Most metabarcoding studies use a single primer pair, but multiple primer pairs (species‐specific or not) has been suggested and shown to improve amplification success from community samples (Bucklin et al., 2010, 2016; Clarke et al., 2014; Elbrecht & Leese, 2017; Thomsen et al., 2012). Species detection rates of the three COI fragments in our metabarcoded mock communities were expected to be higher than species amplification success during the qualitative primer testing due to massive parallel sequencing and high level of sensitivity. This was generally true but species detection varied across the three COI fragments presumably due to primer biases (see Supporting information Figure S1 for comparison). The majority of species were detected by all three COI primer pairs across all mock communities. However, few taxonomic groups were detected by one COI primer pair alone or two COI primer pairs, such as Harpacticoida with only the Leray fragment and Stolidobranchia with only the FC and Folmer fragments. The combination of three COI primer pairs did improve the overall species detection rates in both primer testing and metabarcoding mock communities. Multiple COI primer pairs (species‐specific or most taxonomic coverage) have been used to increase the number of species amplified and the taxonomic resolution in other studies (Clarke et al., 2014; Letendu et al., 2014; Thomsen et al., 2012). The multiple COI primer pairs covering different regions of the same marker in this study were found to improve species detection rates in both SIS (3.8%–7.5%) and MIS (3.3%–16.7%) mock communities. However, degenerate COI primer pairs have been shown to have better species detection rates than nondegenerate primers (Elbrecht & Leese, 2017) when very broad taxonomic groups are investigated. Therefore, the use of degenerate reverse primer for the Leray and Folmer fragments may farther improve the species recovery rates. The use of multiple primers pairs can be applied as an alternative approach for the markers without such fully degenerated primers available.

### 4.2 Marker choice

It is generally accepted that the choice of metabarcoding marker can greatly affect species estimates (Bucklin et al., 2016; Cristescu, 2014; Tang et al., 2012). Nevertheless, only a limited number of metabarcoding studies have used a multigene approach, and the use of multiple evolutionarily independent markers has even more rarely been sequenced in a single NGS run. A few metabarcoding biodiversity studies have compared 18S and COI markers, with results varying across different taxonomic groups. Drummond et al. (2015) reported both COI and 18S markers providing good proxies to a traditional biodiversity survey dataset for soil eDNA. Tang et al. (2012) reported that COI in eDNA surveys of meiofauna estimated more species than morphospecies (species identified by morphology), whereas 18S underestimated species richness. However, both of these studies lacked a dataset with known species and abundances that could groundtruth results by cross‐validation. By examining mock zooplankton communities, Clarke et al. (2017) reported COI having similar taxonomic coverage of zooplankton phyla as 18S but resolving up to threefold more taxa to species compared to 18S.

Our results suggest that different species and taxonomic groups were detected using the evolutionary independent markers 18S and COI. For example, the orders Cyclopoida, Cardiida, and Neogastropoda were only detected with the 18S marker, while the order Thecosomata was only detected with the COI marker. Despite using a local reference database, we experienced some difficulties with assigning taxonomy to some 18S reads in certain species, where reverse reads matched multiple reference sequences for closely related species (Prokopowich, Gregory, & Crease, 2003; Tang et al., 2012). Brown et al. (2015) listed problematic species for taxonomic assignment using 18S, such as *Artemia* species, *Balanus* species, and *Daphnia* species due to high congeneric sequence similarity, as well as *Corbicula fluminea, Diaphanosoma brachyurum, Eurytemora affinis, Leptodora kindtii, Macrocyclops albidus,* and *Pseudocalanus mimus* due to high intraspecific and sometime intraindividual variation in the 18S V4 region. The difficulty assigning 18S reads to species led to lower species detection in metabarcoding than during primer testing and often resulted in taxonomic identification to a higher level (e.g., genus or family). Furthermore, we experienced difficulty amplifying the COI marker for crustacean groups such as *Calanus, Oithona,* and *Pseudocalanus*. These groups were also reported as problematic for amplification in Young, Abbott, Therriault, and Adamowicz (2016). No major difficulties were encountered when assigning COI reads to the corresponding species, and we were able to distinguish closely related species from the same genus. However, many species were only detected with either 18S or COI, likely due to the low amplification success of the COI primer pairs and the inability to taxonomically identify 18S sequences due to conserved sequences among related species. Overall, the combination of 18S and COI improved species detection rates by 11%–30% compared to using a single 18S or COI marker with the tested primers.

Sequencing depth is often of major concern for fully describing community members from a complex sample. The number of libraries pooled in one sequencing run affects the number of reads per species (Letendu et al., 2014; Shaw et al., 2017). As expected, we found that the number of reads per individual or species varied significantly across markers and fragments. We consider that efficient equimolar quantifications prior to pooling including amplicons of similar length and adjusted bioinformatics pipelines could potentially also counter this variation. On a more positive note, the number of reads assigned to each species and overall species detection rates were consistent whether using a single‐marker or multimarker metabarcoding approach. Therefore, the sequencing depth and species detection rates were not affected using multiple markers in one sequencing run, indicating that multiplexing several primer pairs and markers can provide a robust method to characterize samples without appreciably sacrificing read depth or species detection.

Our study compares species detection success in zooplankton metabarcoding using two evolutionarily independent markers combined with different primer pairs of the same marker. It is important to recognize that the relatively high species recovery we report might not be achieved in studies applying different bioinformatics steps such as implementing OTU clustering methods or using online reference databases which are likely to increase both false positives and false negatives. With the increasing data output from NGS technologies and the ability to pool libraries for sequencing, our results support the use of multiple genetic markers as a cost‐effective approach to assessing biodiversity in a broad range of taxa within the same run. This approach also provides a built‐in means to cross‐validate species detection among the markers. PCR‐free methods have been developed to avoid PCR bias and to enable use of more markers (Liu et al., 2013; Zhou et al., 2013). Through this study, the use of two evolutionarily independent markers significantly improved species detection rates, and the use of three COI primer pairs improved species detection rates for particular taxa.

## 5 CONCLUSIONS

Most metabarcoding studies to date have sequenced single markers, but the choice of marker is known to greatly affect species estimates and detection accuracy. Our results suggest that a multiplexed metabarcoding approach using multiple markers and multiple primer pairs can ultimately achieve more accurate biodiversity estimates by reducing both false positives and negatives. Furthermore, the sequencing depth (number of reads per species) and species detection rates remained consistent whether multiplexing multiple fragments or using a single marker. Overall, our metabarcoding approach utilizing multiple markers and multiple primer pairs improved the species detection rates compared to using a single primer pair and/or marker. Thus, metabarcoding based on multiplexed fragments can be cost‐effective and useful for biomonitoring zooplankton in natural communities.

## CONFLICT OF INTEREST

None declared.

## DATA ACCESSIBILITY

The scripts used for bioinformatics analysis are available in the Appendix. Sequences of the mock communities are available in Dryad Digital Repository: https://doi.org/10.5061/dryad.m83jc20
