# Supplementary material for: Metabarcoding using multiplexed markers increases species detection in complex zooplankton communities
Source: Evol Appl. 2018 Sep 15;11(10):1901–14. doi: 10.1111/eva.12694 (PMC6231476; doi:10.1111/eva.12694)
Supplement: Supplementary file 1 [file EVA-11-1901-s001.docx]

**Metabarcoding using multiplexed markers increases species detection**

**in complex zooplankton communities**

Guang K. Zhang,* Frédéric J.J. Chain,*† Cathryn L. Abbott,¶ and Melania E. Cristescu*

**Table S1**: The complete list of primers used for primer testing, and the 4 primer pairs used in the metabarcoding study are in bold. The fragment name refers to the 14 primer pairs tested in the supporting information table S2. *Note that the 18S fragment varies in length for different species.

| **Fragment** | **Primer Name** | **Sequence (5' - 3')** | **Direction** | **Target Taxa** | **Reference** | **Fragment Size** |
| --- | --- | --- | --- | --- | --- | --- |
| **18S** | **Uni18S** | **AGGGCAAKYCTGGTGCCAGC** | **F** | **Metazoan** | **Zhan et al., 2013** | **310-620*** |
|  | **Uni18SR** | **GRCGGTATCTRATCGYCTT** | **R** | **Metazoan** | **Zhan et al., 2013** |  |
| **COI_FC** | **LCO1490** | **GGTCAACAAATCATAAAGATATTGG** | **F** | **Various phyla** | **Folmer et al., 1994** | **325** |
|  | **Ill_C_R** | **GGIGGRTAIACIGTTCAICC** | **R** | **Arthropoda** | **Shokralla et al., 2015** |  |
| **COI_Leray** | **mlCOIintF** | **GGWACWGGWTGAACWGTWTAYCCYCC** | **F** | **Various phyla** | **Leray et al., 2013** | **313** |
|  | **HCO2198** | **TAAACTTCAGGGTGACCAAAAAATCA** | **R** | **Various phyla** | **Folmer et al., 1994** |  |
| **COI_Folmer** | **LCO1490** | **GGTCAACAAATCATAAAGATATTGG** | **F** | **Various phyla** | **Folmer et al., 1994** | **658** |
|  | **HCO2198** | **TAAACTTCAGGGTGACCAAAAAATCA** | **R** | **Various phyla** | **Folmer et al., 1994** |  |
| COI_Leray2 | mlCOIintF | GGWACWGGWTGAACWGTWTAYCCYCC | F | Various phyla | Leray et al., 2013 | 313 |
|  | jgHCO2198 | TAIACYTCIGGRTGICCRAARAAYCA | R | Invertebrates | Geller et al., 2013 |  |
| COI_Prosser | ZplankF1_t1 | TCTASWAATCATAARGATATTGG | F | Zooplankton | Prosser et al., 2013 | 663 |
|  | ZplankR1_t1 | TTCAGGRTGRCCRAARAATCA | R | Zooplankton | Prosser et al., 2013 |  |
| COI_Geller | jgLCO1490 | TITCIACIAAYCAYAARGAYATTGG | F | Invertebrates | Geller et al., 2013 | 658 |
|  | jgHCO2198 | TAIACYTCIGGRTGICCRAARAAYCA | R | Invertebrates | Geller et al., 2013 |  |
| COI_Meyer | dgLCO1490 | GGTCAACAAATCATAAAGAYATYGG | F | Mollusca | Meyer 2003 | 658 |
|  | dgHCO2198 | TAAACTTCAGGGTGACCAAARAAYCA | R | Mollusca | Meyer 2003 |  |
| COI_Radulovici | CrustDF1 | GGTCWACAAAYCATAAAGAYATTGG | F | Crustacea | Radulovici et al., 2009 | 658 |
|  | CrustDR1 | TAAACYTCAGGRTGACCRAARAAYCA | R | Crustacea | Radulovici et al., 2009 |  |
| COI_Meusnier | Uni-MinibarF1 | CAAAATCATAATGAAGGCATGAGC | F | Various phyla | Meusnier et al., 2008 | 130 |
|  | Uni-MinibarR1 | TCCACTAATCACAARGATATTGGTAC | R | Various phyla | Meusnier et al., 2008 |  |
| COI_Niels | TS2AscF2 | TCNACHAAYCATAARGATATT | F | Tunicates | Niels Van Steenkiste (DFO) | 663 |
|  | TS2AscR2 | ACYTCNGGRTGNCYAAAAAAYCA | R | Tunicates | Niels Van Steenkiste (DFO) |  |
| COI_Lobo | LoboF1 | KBTCHACAAAYCAYAARGAYATHGG | F | Marine metazoans | Lobo et al., 2013 | 658 |
|  | LoboR1 | TGRTTYTTYGGWCAYCCWGARGTTTA | R | Marine metazoans | Lobo et al., 2013 |  |
| COI_CostaF1 | CrustF1 | TTTTCTACAAATCATAAAGACATTGG | F | Crustacea | Costa et al., 2007 | 658 |
|  | HCO2198 | TAAACTTCAGGGTGACCAAAAAATCA | R | Various phyla | Folmer et al., 1994 |  |
| COI_CostaF2 | CrustF2 | GGTTCTTCTCCACCAACCACAARGAYATHGG | F | Crustacea | Costa et al., 2007 | 658 |
|  | HCO2198 | TAAACTTCAGGGTGACCAAAAAATCA | R | Various phyla | Folmer et al., 1994 |  |

**Table S2**: A list of species (n = 104) tested with one 18S primer pair and 13 COI primer pairs (“0” refers to not amplified; “1” refers to successfully amplified with PCR as indicated by a gel electrophoresis band). Species/genera/family included in the mock communities are highlighted in grey. The 14 primer pairs tested were: **1**) 18S (Uni18S/Uni18SR), **2**) COI_FC (LCO1490/ Ill_C_R), **3**) COI_Leray (mlCOIintF/ HCO2198), **4**) COI_Folmer (LCO1490/ HCO2198), **5**) COI_Leray2 (mlCOIintF/ gHCO2198), **6**) COI_Prosser ( ZplankF1_t1/ ZplankR1_t1), **7**) COI_Geller (jgLCO1490/ gHCO2198), **8**) COI_Meyer (dgLCO1490/dgHCO2198), **9**) COI_Radulovici (CrustDF1/CrustDR1), **10**) COI_Meusnier (Uni-MinibarF1/Uni-MinibarR1), **11**) COI_Niels (TS2AscF2/TS2AscR2), **12**) COI_Lobo (LoboF1/HCO2198), **13**) COI_CostaF1 (CrustF1/HCO2198), **14**) COI_CostaF2 (CrustF2/ HCO2198).

| **Species tested** | **1** | **2** | **3** | **4** | **5** | **6** | **7** | **8** | **9** | **10** | **11** | **12** | **13** | **14** |
| --- | --- | --- | --- | --- | --- | --- | --- | --- | --- | --- | --- | --- | --- | --- |
| Annelida (n = 1) | 1 | 0 | 0 | 0 | 0 | 1 | 0 | 0 | 0 | 0 | 0 | 0 | 0 | 0 |
| *Tubifex tubifex* | 1 | 0 | 0 | 0 | 0 | 1 | 0 | 0 | 0 | 0 | 0 | 0 | 0 | 0 |
| Arthropoda (n = 73) | 53 | 36 | 28 | 29 | 22 | 26 | 31 | 33 | 41 | 19 | 16 | 0 | 20 | 24 |
| Amphipoda | 4 | 6 | 4 | 1 | 0 | 4 | 3 | 4 | 5 | 2 | 2 | 0 | 1 | 3 |
| *Caprella mutica* | 0 | 1 | 1 | 0 | 0 | 1 | 1 | 1 | 1 | 0 | 1 | 0 | 0 | 1 |
| Crangonyx | 1 | 1 | 0 | 0 | 0 | 0 | 0 | 0 | 0 | 1 | 0 | 0 | 0 | 0 |
| *Gammarus lawrencianus* | 0 | 1 | 1 | 0 | 0 | 1 | 0 | 0 | 1 | 0 | 0 | 0 | 0 | 0 |
| *Gammarus oceanicus* | 1 | 1 | 1 | 0 | 0 | 0 | 1 | 1 | 1 | 1 | 1 | 0 | 1 | 1 |
| *Gammarus spp.* | 1 | 1 | 1 | 1 | 0 | 1 | 1 | 1 | 1 | 0 | 0 | 0 | 0 | 1 |
| *Hyalella azteca* | 1 | 1 | 0 | 0 | 0 | 1 | 0 | 1 | 1 | 0 | 0 | 0 | 0 | 0 |
| Anostraca | 1 | 1 | 1 | 1 | 1 | 0 | 1 | 1 | 1 | 1 | 0 | 0 | 1 | 0 |
| *Artemia salina* | 1 | 1 | 1 | 1 | 1 | 0 | 1 | 1 | 1 | 1 | 0 | 0 | 1 | 0 |
| Calanoida | 10 | 5 | 5 | 3 | 2 | 2 | 6 | 6 | 8 | 5 | 0 | 0 | 0 | 2 |
| *Acartia hudsonica* | 1 | 0 | 0 | 0 | 0 | 0 | 0 | 0 | 1 | 0 | 0 | 0 | 0 | 0 |
| *Acartia longiremis* | 1 | 1 | 0 | 0 | 0 | 0 | 0 | 0 | 1 | 0 | 0 | 0 | 0 | 0 |
| *Centropages hamatus* | 0 | 1 | 1 | 1 | 1 | 1 | 1 | 1 | 1 | 1 | 0 | 0 | 0 | 1 |
| *Epischura lacustris* | 1 | 1 | 1 | 0 | 1 | 0 | 1 | 0 | 0 | 1 | 0 | 0 | 0 | 1 |
| *Eurytemora herdmani* | 1 | 0 | 0 | 0 | 0 | 0 | 0 | 0 | 0 | 0 | 0 | 0 | 0 | 0 |
| *Metridia pacifica* | 0 | 1 | 1 | 0 | 0 | 0 | 0 | 0 | 0 | 0 | 0 | 0 | 0 | 0 |
| *Microcalanus pusillus* | 0 | 0 | 0 | 0 | 0 | 0 | 0 | 0 | 0 | 1 | 0 | 0 | 0 | 0 |
| *Paracalanus spp.* | 1 | 0 | 0 | 0 | 0 | 0 | 0 | 0 | 0 | 0 | 0 | 0 | 0 | 0 |
| *Pseudocalanus newmani* | 1 | 0 | 1 | 0 | 0 | 1 | 1 | 1 | 1 | 0 | 0 | 0 | 0 | 0 |
| *Pseudodiaptomus* | 0 | 0 | 0 | 0 | 0 | 0 | 0 | 0 | 0 | 1 | 0 | 0 | 0 | 0 |
| *Senecella calanoides* | 1 | 0 | 0 | 0 | 0 | 0 | 1 | 1 | 1 | 0 | 0 | 0 | 0 | 0 |
| *Skistodiaptomus oregonensis* | 1 | 0 | 0 | 1 | 0 | 0 | 1 | 1 | 1 | 0 | 0 | 0 | 0 | 0 |
| *Temora longicornis* | 1 | 0 | 0 | 0 | 0 | 0 | 0 | 1 | 1 | 1 | 0 | 0 | 0 | 0 |
| *Tortanus discaudatus* | 1 | 1 | 1 | 1 | 0 | 0 | 1 | 1 | 1 | 0 | 0 | 0 | 0 | 0 |
| Cirripedia | 3 | 3 | 2 | 2 | 2 | 2 | 2 | 2 | 3 | 2 | 2 | 0 | 2 | 2 |
| *Balanus crenatus* | 1 | 1 | 0 | 0 | 0 | 0 | 0 | 0 | 1 | 0 | 0 | 0 | 0 | 0 |
| *Chthamalus dalli* | 1 | 1 | 1 | 1 | 1 | 1 | 1 | 1 | 1 | 1 | 1 | 0 | 1 | 1 |
| Cirripedia cyprid | 1 | 1 | 1 | 1 | 1 | 1 | 1 | 1 | 1 | 1 | 1 | 0 | 1 | 1 |
| Cladocera | 19 | 11 | 6 | 11 | 7 | 8 | 9 | 10 | 11 | 3 | 7 | 0 | 6 | 9 |
| *Alona affinis* | 1 | 1 | 0 | 0 | 0 | 0 | 0 | 0 | 0 | 0 | 0 | 0 | 0 | 0 |
| *Bosmina coregoni* | 1 | 0 | 0 | 0 | 0 | 0 | 0 | 0 | 0 | 0 | 0 | 0 | 0 | 0 |
| *Bythotrephes longimanus* | 1 | 1 | 1 | 1 | 1 | 1 | 1 | 1 | 1 | 0 | 0 | 0 | 1 | 1 |
| *Cercopagis pengoi* | 1 | 1 | 0 | 1 | 0 | 0 | 0 | 1 | 1 | 0 | 0 | 0 | 0 | 0 |
| *Ceriodaphnia lacustris* | 1 | 1 | 1 | 1 | 1 | 1 | 1 | 1 | 1 | 0 | 1 | 0 | 0 | 1 |
| *Chydorus globosus* | 1 | 0 | 0 | 0 | 0 | 0 | 0 | 0 | 1 | 0 | 0 | 0 | 0 | 0 |
| *Daphnia ambigua* | 1 | 0 | 0 | 1 | 0 | 1 | 1 | 1 | 1 | 0 | 1 | 0 | 1 | 1 |
| *Daphnia dentifera* | 0 | 0 | 0 | 0 | 0 | 0 | 0 | 0 | 0 | 0 | 0 | 0 | 0 | 0 |
| *Daphnia magna* | 1 | 1 | 1 | 1 | 0 | 1 | 1 | 1 | 1 | 0 | 1 | 0 | 1 | 1 |
| *Daphnia mendotae* | 0 | 0 | 0 | 1 | 0 | 1 | 1 | 1 | 1 | 0 | 1 | 0 | 0 | 0 |
| *Daphnia obtusa* | 1 | 0 | 0 | 1 | 1 | 0 | 1 | 1 | 1 | 1 | 1 | 0 | 0 | 1 |
| *Daphnia pulex* | 1 | 1 | 1 | 1 | 0 | 1 | 1 | 1 | 0 | 0 | 1 | 0 | 1 | 1 |
| *Daphnia pulicaria* | 1 | 0 | 0 | 1 | 1 | 1 | 1 | 1 | 1 | 0 | 1 | 0 | 1 | 1 |
| *Diaphanosoma brachyurum* | 1 | 0 | 0 | 0 | 0 | 0 | 0 | 0 | 1 | 0 | 0 | 0 | 0 | 0 |
| *Eurycercus lamellatus* | 0 | 1 | 0 | 0 | 0 | 0 | 0 | 0 | 0 | 0 | 0 | 0 | 0 | 0 |
| *Evadne spp.* | 1 | 0 | 0 | 0 | 0 | 0 | 0 | 0 | 0 | 0 | 0 | 0 | 0 | 0 |
| *Holopedium gibberum* | 1 | 1 | 1 | 0 | 1 | 1 | 1 | 1 | 1 | 1 | 0 | 0 | 1 | 1 |
| *Leptodora kindtii* | 1 | 1 | 0 | 0 | 0 | 0 | 0 | 0 | 0 | 0 | 0 | 0 | 0 | 0 |
| *Pleopsis polyphemoides* | 1 | 0 | 0 | 1 | 1 | 0 | 0 | 0 | 0 | 0 | 0 | 0 | 0 | 0 |
| *Pleuroxus procurvus* | 0 | 0 | 0 | 0 | 0 | 0 | 0 | 0 | 0 | 0 | 0 | 0 | 0 | 0 |
| *Podon spp.* | 1 | 0 | 0 | 0 | 0 | 0 | 0 | 0 | 0 | 0 | 0 | 0 | 0 | 0 |
| *Polyphemus pediculus* | 1 | 1 | 1 | 1 | 1 | 0 | 0 | 0 | 0 | 1 | 0 | 0 | 0 | 0 |
| *Sida crystallina* | 1 | 0 | 0 | 0 | 0 | 0 | 0 | 0 | 0 | 0 | 0 | 0 | 0 | 0 |
| *Simocephalus* | 0 | 1 | 0 | 0 | 0 | 0 | 0 | 0 | 0 | 0 | 0 | 0 | 0 | 1 |
| Cyclopoida | 4 | 1 | 1 | 1 | 1 | 1 | 1 | 1 | 2 | 0 | 0 | 0 | 1 | 0 |
| *Eucyclops speratus* | 1 | 0 | 0 | 0 | 0 | 0 | 0 | 0 | 0 | 0 | 0 | 0 | 0 | 0 |
| *Macrocyclops albidus* | 1 | 0 | 0 | 0 | 0 | 0 | 0 | 0 | 1 | 0 | 0 | 0 | 0 | 0 |
| *Mesocyclops edax* | 1 | 1 | 1 | 1 | 1 | 1 | 1 | 1 | 1 | 0 | 0 | 0 | 1 | 0 |
| *Oithona similis* | 1 | 0 | 0 | 0 | 0 | 0 | 0 | 0 | 0 | 0 | 0 | 0 | 0 | 0 |
| *Oithona spp.* | 0 | 0 | 0 | 0 | 0 | 0 | 0 | 0 | 0 | 0 | 0 | 0 | 0 | 0 |
| Decapoda | 8 | 8 | 7 | 8 | 8 | 7 | 8 | 8 | 9 | 5 | 5 | 0 | 8 | 6 |
| Cancridae | 1 | 0 | 0 | 0 | 0 | 0 | 0 | 0 | 1 | 0 | 0 | 0 | 0 | 0 |
| Grapsidae | 0 | 0 | 0 | 0 | 0 | 0 | 0 | 0 | 0 | 0 | 0 | 0 | 0 | 0 |
| *Hemigrapsus nudus* | 0 | 1 | 1 | 1 | 1 | 1 | 1 | 1 | 1 | 0 | 1 | 0 | 1 | 1 |
| *Hemigrapsus oregonensis* | 1 | 1 | 1 | 1 | 1 | 1 | 1 | 1 | 1 | 1 | 0 | 0 | 1 | 1 |
| *Lophopanopeus bellus* | 1 | 1 | 1 | 1 | 1 | 1 | 1 | 1 | 1 | 1 | 1 | 0 | 1 | 0 |
| Majidae | 0 | 0 | 0 | 0 | 0 | 0 | 0 | 0 | 0 | 0 | 0 | 0 | 1 | 0 |
| *Metacarcinus magister* | 1 | 0 | 1 | 1 | 1 | 0 | 1 | 1 | 1 | 0 | 0 | 0 | 1 | 1 |
| Paguroidea; hermit crab | 1 | 1 | 1 | 1 | 1 | 1 | 1 | 1 | 1 | 1 | 1 | 0 | 0 | 1 |
| *Phyllolithodes papillosus* | 1 | 1 | 0 | 1 | 1 | 1 | 1 | 1 | 1 | 1 | 1 | 0 | 1 | 1 |
| *Pugettia gracilis* | 1 | 1 | 1 | 1 | 1 | 1 | 1 | 1 | 1 | 0 | 1 | 0 | 1 | 1 |
| *Scyra acutifrons* | 0 | 1 | 0 | 0 | 0 | 0 | 0 | 0 | 0 | 0 | 0 | 0 | 0 | 0 |
| Xanthidae | 1 | 1 | 1 | 1 | 1 | 1 | 1 | 1 | 1 | 1 | 0 | 0 | 1 | 0 |
| Diptera | 0 | 0 | 0 | 0 | 0 | 0 | 0 | 0 | 0 | 0 | 0 | 0 | 0 | 0 |
| *Tachididae* | 0 | 0 | 0 | 0 | 0 | 0 | 0 | 0 | 0 | 0 | 0 | 0 | 0 | 0 |
| Ephemeroptera | 1 | 0 | 1 | 1 | 1 | 1 | 1 | 1 | 0 | 0 | 0 | 0 | 1 | 1 |
| *Hexagenia* | 1 | 0 | 1 | 1 | 1 | 1 | 1 | 1 | 0 | 0 | 0 | 0 | 1 | 1 |
| Harpacticoida | 1 | 1 | 0 | 0 | 0 | 0 | 0 | 0 | 1 | 1 | 0 | 0 | 0 | 0 |
| *Clytemnestra scutellata* | 0 | 0 | 0 | 0 | 0 | 0 | 0 | 0 | 1 | 0 | 0 | 0 | 0 | 0 |
| *Microsetella norvegica* | 0 | 1 | 0 | 0 | 0 | 0 | 0 | 0 | 0 | 1 | 0 | 0 | 0 | 0 |
| Unknown Harpacticoid | 1 | 0 | 0 | 0 | 0 | 0 | 0 | 0 | 0 | 0 | 0 | 0 | 0 | 0 |
| Isopoda | 1 | 0 | 1 | 1 | 0 | 1 | 0 | 0 | 1 | 0 | 0 | 0 | 0 | 1 |
| Unknown Isopoda | 1 | 0 | 1 | 1 | 0 | 1 | 0 | 0 | 1 | 0 | 0 | 0 | 0 | 1 |
| Mysida | 0 | 0 | 0 | 0 | 0 | 0 | 0 | 0 | 0 | 0 | 0 | 0 | 0 | 0 |
| *Hemimysis anomala* | 0 | 0 | 0 | 0 | 0 | 0 | 0 | 0 | 0 | 0 | 0 | 0 | 0 | 0 |
| Poecilostomatoida | 1 | 0 | 0 | 0 | 0 | 0 | 0 | 0 | 0 | 0 | 0 | 0 | 0 | 0 |
| *Corycaeus anglicus* | 1 | 0 | 0 | 0 | 0 | 0 | 0 | 0 | 0 | 0 | 0 | 0 | 0 | 0 |
| Chordata (n = 11) | 10 | 6 | 4 | 5 | 5 | 4 | 6 | 7 | 5 | 7 | 4 | 0 | 1 | 2 |
| Actinopterygii | 2 | 2 | 2 | 1 | 2 | 2 | 2 | 2 | 2 | 2 | 1 | 0 | 1 | 2 |
| Unknown Fish | 1 | 1 | 1 | 1 | 1 | 1 | 1 | 1 | 1 | 1 | 1 | 0 | 0 | 1 |
| *Gasterosteus aculeatus* | 1 | 1 | 1 | 0 | 1 | 1 | 1 | 1 | 1 | 1 | 0 | 0 | 1 | 1 |
| Tunicata | 8 | 4 | 2 | 4 | 3 | 2 | 4 | 5 | 3 | 5 | 3 | 0 | 0 | 0 |
| *Botryllus schlosseri* | 1 | 1 | 0 | 0 | 0 | 0 | 0 | 1 | 1 | 0 | 0 | 0 | 0 | 0 |
| *cfr. Cnemidocarpa finmarkiensis* | 1 | 1 | 1 | 1 | 1 | 0 | 1 | 1 | 1 | 1 | 1 | 0 | 0 | 0 |
| *Ciona intestinalis* | 1 | 0 | 0 | 0 | 0 | 0 | 0 | 0 | 0 | 0 | 0 | 0 | 0 | 0 |
| *Eudistoma molle?* | 1 | 0 | 0 | 0 | 0 | 1 | 0 | 0 | 0 | 0 | 0 | 0 | 0 | 0 |
| *Halocynthia aurantium* | 1 | 1 | 0 | 1 | 1 | 0 | 1 | 1 | 0 | 1 | 1 | 0 | 0 | 0 |
| *Oikopleura* | 1 | 0 | 0 | 0 | 0 | 0 | 0 | 0 | 0 | 1 | 0 | 0 | 0 | 0 |
| *Pyura haustor* | 1 | 0 | 0 | 0 | 0 | 0 | 0 | 0 | 0 | 0 | 0 | 0 | 0 | 0 |
| *Styela clava* | 0 | 0 | 0 | 1 | 0 | 0 | 1 | 1 | 0 | 1 | 0 | 0 | 0 | 0 |
| *Styela gibbsii* | 1 | 1 | 1 | 1 | 1 | 1 | 1 | 1 | 1 | 1 | 1 | 0 | 0 | 0 |
| Mollusca (n = 12) | 8 | 9 | 7 | 3 | 5 | 5 | 7 | 6 | 8 | 2 | 3 | 0 | 4 | 1 |
| Bivalvia | 6 | 7 | 6 | 0 | 4 | 4 | 4 | 4 | 6 | 2 | 3 | 0 | 3 | 1 |
| *Corbicula fluminea* | 1 | 1 | 1 | 0 | 1 | 1 | 1 | 1 | 1 | 0 | 0 | 0 | 1 | 0 |
| *Crassostrea gigas* | 1 | 1 | 1 | 0 | 1 | 1 | 1 | 1 | 1 | 0 | 1 | 0 | 1 | 1 |
| *Leukoma staminea* | 0 | 1 | 1 | 0 | 0 | 0 | 0 | 0 | 0 | 0 | 0 | 0 | 0 | 0 |
| *Limnoperna fortunei* | 1 | 1 | 1 | 0 | 1 | 1 | 1 | 1 | 1 | 1 | 1 | 0 | 1 | 0 |
| *Macoma secta* | 1 | 1 | 1 | 0 | 1 | 1 | 1 | 1 | 1 | 0 | 1 | 0 | 0 | 0 |
| *Mytilus edulis* | 2 | 2 | 1 | 0 | 0 | 0 | 0 | 0 | 2 | 1 | 0 | 0 | 0 | 0 |
| *Ruditapes philippinarum* | 0 | 0 | 0 | 0 | 0 | 0 | 0 | 0 | 0 | 0 | 0 | 0 | 0 | 0 |
| *Saxidomas gigantea* | 0 | 0 | 0 | 0 | 0 | 0 | 0 | 0 | 0 | 0 | 0 | 0 | 0 | 0 |
| Gastropoda | 1 | 2 | 1 | 2 | 1 | 1 | 2 | 1 | 1 | 0 | 0 | 0 | 1 | 0 |
| *Limacina helicina* | 1 | 1 | 1 | 1 | 1 | 1 | 1 | 1 | 1 | 0 | 0 | 0 | 1 | 0 |
| *Nassarius distortus* | 0 | 0 | 0 | 0 | 0 | 0 | 0 | 0 | 0 | 0 | 0 | 0 | 0 | 0 |
| *Nerita spp.* | 0 | 1 | 0 | 1 | 0 | 0 | 1 | 0 | 0 | 0 | 0 | 0 | 0 | 0 |
| Pteropoda | 1 | 0 | 0 | 1 | 0 | 0 | 1 | 1 | 1 | 0 | 0 | 0 | 0 | 0 |
| Unknown Pteropoda | 1 | 0 | 0 | 1 | 0 | 0 | 1 | 1 | 1 | 0 | 0 | 0 | 0 | 0 |
| Platyhelminthes (n = 1) | 1 | 0 | 0 | 0 | 0 | 0 | 0 | 0 | 1 | 0 | 0 | 0 | 0 | 0 |
| beige flatworm | 1 | 0 | 0 | 0 | 0 | 0 | 0 | 0 | 1 | 0 | 0 | 0 | 0 | 0 |
| Rotifera (n = 5) | 5 | 0 | 1 | 2 | 1 | 3 | 2 | 4 | 5 | 3 | 0 | 0 | 0 | 1 |
| *Brachionus calyciflorus* | 1 | 0 | 1 | 1 | 1 | 1 | 1 | 1 | 1 | 1 | 0 | 0 | 0 | 0 |
| *Brachionus rubens* | 1 | 0 | 0 | 1 | 0 | 1 | 1 | 1 | 1 | 1 | 0 | 0 | 0 | 0 |
| *Cephalodella acidophila* | 1 | 0 | 0 | 0 | 0 | 1 | 0 | 1 | 1 | 1 | 0 | 0 | 0 | 0 |
| *Keratella cochlearis* | 1 | 0 | 0 | 0 | 0 | 0 | 0 | 0 | 1 | 0 | 0 | 0 | 0 | 0 |
| *Keratella quadrata* | 1 | 0 | 0 | 0 | 0 | 0 | 0 | 1 | 1 | 0 | 0 | 0 | 0 | 1 |
| Total (n = 103)  % | 78 (75) | 51 (50) | 40 (39) | 39 (38) | 33 (32) | 39 (38) | 46 (45) | 50 (49) | 60 (58) | 31 (30) | 23 (22) | 0 (0) | 25 (24) | 28 (27) |

**Table S3**: Mock communities species list, where ‘n’ refers to the number of individuals (1a-1g: single individuals per species (SIS); 2a-2g: multiple individuals per species (MIS); 3a1-3d3: populations of single species (PSS)). The libraries 1e and 2e were assembled using the pooled DNA from four separate DNA extractions: 1a-1d and 2a-2d respectively (DNA extraction and dilutions are described in Brown *et al.* 2015).

| **Library** | **Order** | **Family** | | **Species** | **n** | **Provider** |
| --- | --- | --- | --- | --- | --- | --- |
| **Single Individuals per Species (‘SIS’: 1a,1b,1c,1d,1e,1g)** | | | | | | |
| 1a | Cyclopoida | Cyclopidae | *Acanthocyclops vernalis* | | 1 | Brown et al. 2015 |
| 1a | Diplostraca | Bosminidae | *Bosmina longirostris* | | 1 | Brown et al. 2015 |
| 1a | Diplostraca | Daphniidae | *Ceriodaphnia lacustris* | | 1 | Brown et al. 2015 |
| 1a | Diplostraca | Daphniidae | *Daphnia parvula* | | 1 | Brown et al. 2015 |
| 1a | Diplostraca | Daphniidae | *Daphnia pulex* | | 1 | Brown et al. 2015 |
| 1a | Diplostraca | Daphniidae | *Daphnia pulicaria* | | 1 | Brown et al. 2015 |
| 1a | Diplostraca | Holopediidae | *Holopedium gibberum* | | 1 | Brown et al. 2015 |
| 1a | Diplostraca | Leptodoridae | *Leptodora kindtii* | | 1 | Brown et al. 2015 |
| 1a | Diplostraca | Polyphemidae | *Polyphemus pediculus* | | 1 | Brown et al. 2015 |
| 1a | Calanoida | Clausocalanidae | *Pseudocalanus mimus* | | 1 | Brown et al. 2015 |
| 1b | Anostraca | Artemiidae | *Artemia spp* | | 1 | Brown et al. 2015 |
| 1b | Phlebobranchia | Cionidae | *Ciona intestinalis* | | 1 | Brown et al. 2015 |
| 1b | Venerida | Cyrenidae | *Corbicula fluminea* | | 1 | Brown et al. 2015 |
| 1b | Amphipoda | Crangonyctidae | *Crangonyx* | | 1 | Brown et al. 2015 |
| 1b | Myida | Dreissenidae | *Dreissena polymorpha* | | 1 | Brown et al. 2015 |
| 1b | Calanoida | Temoridae | *Eurytemora affinis* | | 1 | Brown et al. 2015 |
| 1b | Amphipoda | Gammaridae | *Gammarus lacustris* | | 1 | Brown et al. 2015 |
| 1b | Amphipoda | Gammaridae | *Gammarus oceanicus* | | 1 | Brown et al. 2015 |
| 1b | Amphipoda | Gammaridae | *Gammarus lawrencianus* | | 1 | Brown et al. 2015 |
| 1b | Amphipoda | Hyalellidae | *Hyalella azteca* | | 1 | Brown et al. 2015 |
| 1b | Amphipoda | Hyalellidae | *Hyalella* clade 1 | | 1 | Brown et al. 2015 |
| 1b | Amphipoda | Hyalellidae | *Hyalella* clade 8 | | 1 | Brown et al. 2015 |
| 1b | Amphipoda | Hyperiidae | *Hyperia galba* | | 1 | Brown et al. 2015 |
| 1b | Amphipoda | Hyperiidae | *Hyperoche mediterranea* | | 1 | Brown et al. 2015 |
| 1b | Mytilida | Mytilidae | *Limnoperna fortunei* | | 1 | Brown et al. 2015 |
| 1b | Amphipoda | Hyperiidae | *Themisto libellula* | | 1 | Brown et al. 2015 |
| 1c | Sessilia | Balanidae | *Balanus crenatus* | | 1 | Brown et al. 2015 |
| 1c | Sessilia | Balanidae | *Balanus glandula* | | 1 | Brown et al. 2015 |
| 1c | Decapoda | Portunidae | *Carcinus maenas* | | 1 | Brown et al. 2015 |
| 1c | Decapoda | Atyidae | *Caridean larvae* | | 1 | Brown et al. 2015 |
| 1c | Sessilia | Chthamalinae | *Chthamalus dalli* | | 1 | Brown et al. 2015 |
| 1c | Decapoda | Crangonidae | *Crangonidae* | | 1 | Brown et al. 2015 |
| 1c | Decapoda | Xanthidae | *Xanthidae* | | 1 | Brown et al. 2015 |
| 1c | Decapoda | Grapsidae | *Grapsidae* | | 1 | Brown et al. 2015 |
| 1c | Thecosomata | Limacinidae | *Limacina helicina* | | 1 | Brown et al. 2015 |
| 1c | Decapoda | Majidae | *Majidae* | | 1 | Brown et al. 2015 |
| 1c | Mytilida | Mytilidae | *Mytilus edulis* | | 1 | Brown et al. 2015 |
| 1c | Neogastropoda | Nassariidae | *Nassarius distortus* | | 1 | Brown et al. 2015 |
| 1c | Cycloneritimorpha | Neritidae | *Nerita spp* | | 1 | Brown et al. 2015 |
| 1c | Decapoda | Hippolytidae | *Hippolytidae* | | 1 | Brown et al. 2015 |
| 1d | Diplostraca | Cercopagididae | *Bythotrephes longimanus* | | 1 | Brown et al. 2015 |
| 1d | Calanoida | Calanidae | *Calanus finmarchicus* | | 1 | Brown et al. 2015 |
| 1d | Calanoida | Centropagidae | *Centropages abdominalis* | | 1 | Brown et al. 2015 |
| 1d | Diplostraca | Sididae | *Diaphanosoma brachyurum* | | 1 | Brown et al. 2015 |
| 1d | Cyclopoida | Cyclopidae | *Eucyclops speratus* | | 1 | Brown et al. 2015 |
| 1d | Harpacticoida | Tachidiidae | *Tachidiidae* | | 1 | Brown et al. 2015 |
| 1d | Calanoida | Diaptomidae | *Leptodiaptomus minutus* | | 1 | Brown et al. 2015 |
| 1d | Calanoida | Centropagidae | *Limnocalanus macrurus* | | 1 | Brown et al. 2015 |
| 1d | Cyclopoida | Cyclopidae | *Macrocyclops albidus* | | 1 | Brown et al. 2015 |
| 1d | Decapoda | Callianassidae | *Neotrypaea californiensis* | | 1 | Brown et al. 2015 |
| 1d | Harpacticoida | Peltidiidae | *Clytemnestra scutellata* | | 1 | Brown et al. 2015 |
| 1d | Copelata | Oikopleuridae | *Oikopleura labradonensis* | | 1 | Brown et al. 2015 |
| 1d | Cyclopoida | Oithonidae | *Oithona atlantica* | | 1 | Brown et al. 2015 |
| 1d | Anaspidea | Aplysiidae | *Pteropoda* | | 1 | Brown et al. 2015 |
| 1d | Harpacticoida | Tisbidae | *Tisbe furcata* | | 1 | Brown et al. 2015 |
| 1d | Harpacticoida | Harpacticidae | *Zaus abbreviatus* | | 1 | Brown et al. 2015 |
| 1e | pooled DNA from 1a-1d | | | | 56 | Brown et al. 2015 |
| 1g | Calanoida | Acartiidae | | *Acartia hudsonica* | 1 | Brown et al. 2015 |
| 1g | Trachymedusae | Rhopalonematidae | | *Aglantha digitale* | 1 | Brown et al. 2015 |
| 1g | Anostraca | Artemiidae | | *Artemia spp* | 1 | Brown et al. 2015 |
| 1g | Isopoda | Asellidae | | *Asellus* | 1 | Brown et al. 2015 |
| 1g | Sessilia | Balanidae | | *Balanus crenatus* | 1 | Brown et al. 2015 |
| 1g | Stolidobranchia | Styelidae | | *Botrylloides violaceus* | 1 | PBS-DF |
| 1g | Stolidobranchia | Styelidae | | *Botryllus schlosseri* | 1 | PBS-DFO |
| 1g | Ploima | Brachionidae | | *Brachionus calyciflorus* | 1 | Brown et al. 2015 |
| 1g | Diplostraca | Cercopagididae | | *Bythotrephes longimanus* | 1 | Brown et al. 2015 |
| 1g | Amphipoda | Caprellidae | | *Caprella mutica* | 1 | PBS-DFO |
| 1g | Diplostraca | Cercopagididae | | *Cercopagis pengoi* | 1 | Brown et al. 2015 |
| 1g | Venerida | Cyrenidae | | *Corbicula fluminea* | 1 | Brown et al. 2015 |
| 1g | Ostreida | Ostreidae | | *Crassostrea gigas* | 1 | PBS-DFO |
| 1g | Diplostraca | Daphniidae | | *Daphnia magna* | 1 | Brown et al. 2015 |
| 1g | Decapoda | Varunidae | | *Hemigrapsus oregonensis* | 1 | PBS-DFO |
| 1g | Mysida | Mysidae | | *Hemimysis anomala* | 1 | Brown et al. 2015 |
| 1g | Amphipoda | Hyalellidae | | *Hyalella azteca* | 1 | Brown et al. 2015 |
| 1g | Ploima | Branchionidae | | *Keratella quadrata* | 1 | Brown et al. 2015 |
| 1g | Thecosomata | Limacinidae | | *Limacina helicina* | 1 | Brown et al. 2015 |
| 1g | Mytilida | Mytilidae | | *Limnoperna fortunei* | 1 | Brown et al. 2015 |
| 1g | Decapoda | Panopeidae | | *Lophopanopeus bellus* | 1 | PBS-DFO |
| 1g | Cardiida | Tellinidae | | *Macoma secta* | 1 | PBS-DFO |
| 1g | Cyclopoida | Cyclopidae | | *Mesocyclops edax* | 1 | Brown et al. 2015 |
| 1g | Decapoda | Cancridae | | *Metacarcinus magister* | 1 | PBS-DFO |
| 1g | Harpacticoida | Ectinosomatidae | | *Microsetella norvegica* | 1 | Brown et al. 2015 |
| 1g | Stolidobranchia | Styelidae | | *Styela clava* | 1 | PBS-DFO |
| 1g | Calanoida | Tortanidae | | *Tortanus discaudatus* | 1 | Brown et al. 2015 |
| **Multiple Individuals per Species (‘MIS’: 2a,2b,2c,2d,2e,2g)** | | | | | | |
| 2a | Sessilia | Balanidae | | *Balanus crenatus* | 10 | Brown et al. 2015 |
| 2a | Amphipoda | Gammaridae | | *Gammarus lawrencianus* | 1 | Brown et al. 2015 |
| 2a | Calanoida | Diaptomidae | | *Leptodiaptomus minutus* | 1 | Brown et al. 2015 |
| 2a | Decapoda | Palaemonidae | | *Palaemonetes spp* | 5 | Brown et al. 2015 |
| 2b | Calanoida | Acartiidae | | *Acartia longiremis* | 5 | Brown et al. 2015 |
| 2b | Anostraca | Artemiidae | | *Artemia spp* | 2 | Brown et al. 2015 |
| 2b | Sessilia | Chthamalinae | | *Chthamalus dalli* | 1 | Brown et al. 2015 |
| 2b | Amphipoda | Hyalellidae | | *Hyalella* clade 8 | 5 | Brown et al. 2015 |
| 2b | Diplostraca | Leptodoridae | | *Leptodora kindtii* | 5 | Brown et al. 2015 |
| 2c | Decapoda | Portunidae | | *Carcinus maenas* | 2 | Brown et al. 2015 |
| 2c | Venerida | Cyrenidae | | *Corbicula fluminea* | 5 | Brown et al. 2015 |
| 2c | Diplostraca | Daphniidae | | *Daphnia pulex* | 10 | Brown et al. 2015 |
| 2c | Cycloneritimorpha | Neritidae | | *Nerita spp* | 1 | Brown et al. 2015 |
| 2d | Calanoida | Temoridae | | *Eurytemora affinis* | 23 | Brown et al. 2015 |
| 2e | pooled DNA from 2a-2d | | | | 76 | Brown et al. 2015 |
| 2g | Calanoida | Acartiidae | | *Acartia hudsonica* | 4 | Brown et al. 2015 |
| 2g | Anostraca | Artemiidae | | *Artemia spp* | 2 | Brown et al. 2015 |
| 2g | Isopoda | Asellidae | | *Asellus* | 1 | Brown et al. 2015 |
| 2g | Sessilia | Balanidae | | *Balanus crenatus* | 3 | Brown et al. 2015 |
| 2g | Stolidobranchia | Styelidae | | *Botrylloides violaceus* | 2 | PBS-DFO |
| 2g | Stolidobranchia | Styelidae | | *Botryllus schlosseri* | 1 | PBS-DFO |
| 2g | Ploima | Brachionidae | | *Brachionus calyciflorus* | 5 | Brown et al. 2015 |
| 2g | Diplostraca | Cercopagididae | | *Bythotrephes longimanus* | 2 | Brown et al. 2015 |
| 2g | Amphipoda | Caprellidae | | *Caprella mutica* | 1 | PBS-DFO |
| 2g | Diplostraca | Cercopagididae | | *Cercopagis pengoi* | 3 | Brown et al. 2015 |
| 2g | Venerida | Cyrenidae | | *Corbicula fluminea* | 2 | Brown et al. 2015 |
| 2g | Ostreida | Ostreidae | | *Crassostrea gigas* | 2 | PBS-DFO |
| 2g | Diplostraca | Daphniidae | | *Daphnia magna* | 3 | Brown et al. 2015 |
| 2g | Decapoda | Varunidae | | *Hemigrapsus oregonensis* | 2 | PBS-DFO |
| 2g | Mysida | Mysidae | | *Hemimysis anomala* | 1 | Brown et al. 2015 |
| 2g | Amphipoda | Hyalellidae | | *Hyalella azteca* | 3 | Brown et al. 2015 |
| 2g | Ploima | Branchionidae | | *Keratella quadrata* | 5 | Brown et al. 2015 |
| 2g | Thecosomata | Limacinidae | | *Limacina helicina* | 1 | Brown et al. 2015 |
| 2g | Mytilida | Mytilidae | | *Limnoperna fortunei* | 3 | Brown et al. 2015 |
| 2g | Decapoda | Panopeidae | | *Lophopanopeus bellus* | 2 | PBS-DFO |
| 2g | Cardiida | Tellinidae | | *Macoma secta* | 1 | PBS-DFO |
| 2g | Cyclopoida | Cyclopidae | | *Mesocyclops edax* | 4 | Brown et al. 2015 |
| 2g | Decapoda | Cancridae | | *Metacarcinus magister* | 1 | PBS-DFO |
| 2g | Harpacticoida | Ectinosomatidae | | *Microsetella norvegica* | 3 | Brown et al. 2015 |
| 2g | Stolidobranchia | Styelidae | | *Styela clava* | 1 | PBS-DFO |
| 2g | Calanoida | Tortanidae | | *Tortanus discaudatus* | 4 | Brown et al. 2015 |
| **Populations of Single Species (‘PSS’: single, low, high number of individuals)** | | | | | | |
| 3a1 | Mytilida | Mytilidae | | *Limnoperna fortunei* | 1 | Brown et al. 2015 |
| 3a2 | Mytilida | Mytilidae | | *Limnoperna fortunei* | 10 | Brown et al. 2015 |
| 3a3 | Mytilida | Mytilidae | | *Limnoperna fortunei* | 30 | Brown et al. 2015 |
| 3b1 | Sessilia | Balanidae | | *Balanus crenatus* | 1 | Brown et al. 2015 |
| 3b2 | Sessilia | Balanidae | | *Balanus crenatus* | 10 | Brown et al. 2015 |
| 3b3 | Sessilia | Balanidae | | *Balanus crenatus* | 17 | Brown et al. 2015 |
| 3c1 | Calanoida | Tortanidae | | *Tortanus discaudatus* | 1 | Brown et al. 2015 |
| 3c2 | Calanoida | Tortanidae | | *Tortanus discaudatus* | 8 | Brown et al. 2015 |
| 3c3 | Calanoida | Tortanidae | | *Tortanus discaudatus* | 15 | Brown et al. 2015 |
| 3d1 | Diplostraca | Leptodoridae | | *Leptodora kindtii* | 1 | Brown et al. 2015 |
| 3d2 | Diplostraca | Leptodoridae | | *Leptodora kindtii* | 10 | Brown et al. 2015 |
| 3d3 | Diplostraca | Leptodoridae | | *Leptodora kindtii* | 28 | Brown et al. 2015 |

**Table S4**: The accession numbers for the references in our local sequence database, including sequences generated in this study, CAISN (Canadian Aquatic Invasive Species) network, or from the BOLD and NCBI online databases. The reference sequences of closely related species were used for the specimens identified at family level and for species without available reference sequences (as indicated in brackets).

| **Species** | **18S-Database** | **18S-Accession** | **COI-Database** | **COI-Accession** |
| --- | --- | --- | --- | --- |
| *Acanthocyclops vernalis* | NA | NA | BOLD | EES04912 |
| *Acartia hudsonica* | CAISN | Pending-XXXX | BOLD-CAISN | CAISN867-13 |
| *Acartia longiremis* | NCBI | GU969156 | This study | Pending-XXXX |
| *Aglantha digitale* | NCBI | EU247821 | BOLD-CAISN | CAISN185-12 |
| *Artemia spp* | NCBI (*Artemia salina*) | X01723 | NCBI (*Artemia franciscana*) | EF615573.1 |
| *Asellus spp* | This study | Pending-XXXX | BOLD (*Asellus aquaticus*) | GU130252 |
| *Balanus crenatus* | This study | Pending-XXXX | This study | Pending-XXXX |
| *Balanus glandula* | NCBI | AF201663.1 | NCBI | KM217564.1 |
| *Bosmina longirostris* | CAISN | BIOUG01746F09 | BOLD | ZPLMX590-06 |
| *Botrylloides violaceus* | NCBI | AY903927.1 | BOLD | GBGC6336-09 |
| *Botryllus schlosseri* | NCBI | FM244858.1 | This study | Pending-XXXX |
| *Brachionus calyciflorus* | This study | Pending-XXXX | This study | Pending-XXXX |
| *Bythotrephes longimanus* | NCBI | AF070094 | BOLD | GBCB0018-06 |
| *Calanus finmarchicus* | NCBI | AF367719 | BOLD (*Calanus glacialis*) | GBCX1672-14 |
| *Caprella mutica* | NCBI (*Caprella equilibra*) | AY743950.1 | BOLD | WWGSL281-08 |
| *Carcinus maenas* | CAISN | BIOUG04840 | BOLD | BNSDE364-14 |
| *Caridean larvae* | NCBI (*Thoralus cranchii*) | EU868758 | BOLD (*Caridina rubella*) | GBCDA445-12 |
| *Centropages abdominalis* | NCBI | GU969163.1 | BOLD | GBA6913-10 |
| *Cercopagis pengoi* | NCBI | EF189620.1 | BOLD | RBGC142-03 |
| *Ceriodaphnia lacustris* | NCBI (*Ceriodaphnia dubia*) | AF144208 | BOLD-CAISN (*Ceriodaphnia dubia*) | CAISN048-12 |
| *Chthamalus dalli* | NCBI | KM974371 | BOLD | GBFCC0231-06 |
| *Ciona intestinalis* | NCBI | AB013017 | BOLD | GBGC0236-06 |
| *Clytemnestra scutellata* | NCBI (*Alteuthellopsis* species) | EU380289.1 | BOLD (Clytemnestrinae) | GBA14468-13 |
| *Corbicula fluminea* | NCBI | EU660782 | BOLD | GBMIN1846-12 |
| *Crangonidae* | NCBI (*Crangon crangon*) | EU920938.1 | BOLD (*Crangon septemspinosa*) | SDP258010-15 |
| *Crangonyx* | NCBI (*Crangonyx forbesi*) | AF202980.1 | BOLD (*Crangonyx gracilis*) | DSMYS144-07 |
| *Crassostrea gigas* | NCBI | AB064942.1 | BOLD | BNAGB698-14 |
| *Daphnia magna* | This study | Pending-XXXX | This study | Pending-XXXX |
| *Daphnia parvula* | NA | NA | BOLD | GBFCE0136-06 |
| *Daphnia pulex* | NCBI | AF014011.1 | BOLD | GBFCE0275-06 |
| *Daphnia pulicaria* | NA | NA | BOLD | GBFCE335-1 |
| *Diaphanosoma brachyurum* | CAISN (*Diaphanosoma* species) | Pending-XXXX | BOLD | GBCB266-07 |
| *Dreissena polymorpha or rostriformis bugensis* | NCBI (*Dreissena polymorpha*) | AF120552.1 | BOLD (*Dreissena polymorpha*) | GBMBV2960-14 |
| *Eucyclops speratus* | NCBI | AJ746333.1 | BOLD | ACSD100-11 |
| *Eurytemora affinis* | NCBI | JX995300.1 | BOLD-CAISN | CAISN901-13 |
| *Gammarus lacustris* | NCBI | EF582915.1 | BOLD-CAISN | CRCN14909 |
| *Gammarus lawrencianus* | NA | NA | NCBI | FJ581660.1 |
| *Gammarus oceanicus* | NCBI (*Gammarus setosus*) | JF966164 | BOLD | ECCRU002-10 |
| *Grapsidae* | NCBI (*Grapsus albolineatus*) | FJ172755.1 | BOLD (*Grapsus adscensionis*) | JSDAZ028-08 |
| *Hemigrapsus oregonensis* | NCBI (*Hemigrapsus sinensis*) | EU284146.1 | BOLD-CAISN | CAISN465-13 |
| *Hemimysis anomala* | This study | Pending-XXXX | BOLD | GBA2802-08 |
| *Hippolytidae* | NCBI (*Hippolyte obliquimanus*) | EU868752.1 | BOLD (*Hippolyte obliquimanus*) | GBCMD13610-13 |
| *Holopedium gibberum* | NCBI | AF070111.1 | BOLD | RBGC149-03 |
| *Hyalella* clade 1 | This study | Pending-XXXX | NCBI | DQ464605.1 |
| *Hyalella* clade 8 | This study | Pending-XXXX | NCBI | DQ464630.1 |
| *Hyalella azteca* | This study | Pending-XXXX | BOLD | CDINV045-07 |
| *Hyperia galba* | NA | NA | NCBI | KT209327.1 |
| *Hyperoche mediterranea* | NCBI (*Hyperoche medusarum*) | KC428897.1 | NCBI (*Hyperoche medusarum*) | EF989667.1 |
| *Keratella quadrata* | NCBI | DQ297697.1 | NCBI | DQ297774.1 |
| *Leptodiaptomus minutus* | NCBI | AY339153.1 | BOLD | GBA3065-08 |
| *Leptodora kindtii* | This study | Pending-XXXX | This study | Pending-XXXX |
| *Limacina helicina* | NA | NA | BOLD | GBMLG12535-13 |
| *Limnocalanus macrurus* | NCBI | HQ407006.1 | BOLD | RBGC101-03 |
| *Limnoperna fortunei* | This study | Pending-XXXX | BOLD | GBMBM348-13 |
| *Lophopanopeus bellus* | This study | Pending-XXXX | BOLD | GBCDA1886-12 |
| *Macoma secta* | NCBI | AY553975.1 | NCBI (*Macoma balthica*) | KP977733.1 |
| *Macrocyclops albidus* | NCBI | DQ538505 | NCBI | KC627343.1 |
| *Majidae* | NCBI (*Maja squinado*) | DQ079758.1 | BOLD (*Maja squinado*) | GBCMA8843-14 |
| *Mesocyclops edax* | NA | NA | BOLD | ZPII1002-11 |
| *Metacarcinus magister* | NCBI | AY527220.1 | BOLD | DSCRA162-07 |
| *Microsetella norvegica* | BOLD-CAISN | BIOUG01750-B03 | BOLD | CAISN1149-13 |
| *Mytilus edulis* | NCBI | KC429331.1 | This study | Pending-XXXX |
| *Nassarius distortus* | NCBI (*Nassarius hepaticus*) | HQ834030.1 | BOLD (*Nassarius hepaticus*) | GBMLS5449-09 |
| *Neotrypaea californiensis* | NCBI | AF436003.1 | BOLD (*Callianassa subterranea*) | BNSDE184-12 |
| *Nerita spp* | This study | Pending-XXXX | This study | Pending-XXXX |
| *Oikopleura labradonensis* | NCBI | FM244869.1 | BOLD (*Oilopleura intermedia*) | GBMIN43462-14 |
| *Oithona atlantica* | CAISN | Pending-XXXX | BOLD-CAISN | CAISN735-13 |
| *Palaemonetes spp* | NCBI (*Palaemonetes vulgaris*) | AY743941.1 | BOLD (*Palaemonetes vulgaris*) | VATWO139-14 |
| *Polyphemus pediculus* | NCBI | EF189633.1 | BOLD | RBGC141-03 |
| *Pseudocalanus mimus* | NCBI (*Pseudocalanus elongatus*) | JX995319.1 | NCBI | AF513651.1 |
| *Pteropoda* | This study | Pending-XXXX | BOLD (*Stylocheilus longicauda*) | GBMLG0219-06 |
| *Styela clava* | This study | Pending-XXXX | NCBI | FJ528635.1 |
| *Tachidiidae* | NCBI (*Tachidius triagularis*) | JQ315760.1 | BOLD (*Tachidius discipes*) | BNSCP121-14 |
| *Themisto libellula* | NCBI | JN039368.1 | NCBI | FJ602467.1 |
| *Tisbe furcata* | CAISN | Pending-XXXX | BOLD-CAISN | CAISN1131-13 |
| *Tortanus discaudatus* | This study | Pending-XXXX | BOLD-CAISN | CAISN1455-14 |
| *Xanthidae* | NCBI (*Xantho poressa*) | FM161989.1 | BOLD (*Xantho hydrophilus*) | MLALN020-10 |
| *Zaus abbreviatus* | NCBI (*Zaus abbreviatus*) | EU380284.1 | BOLD-CAISN | CAISN1170-13 |

**Table S5:** Comparison of species detection and sequencing depth for the 18S V4 marker in experiments where 18S was sequenced alone vs. where 18S was multiplexed with three COI fragments. Both experiments sequenced the same mock community DNA from the Single Individuals per Species (SIS) (library 1a, 1b, 1c, 1d). The inconsistencies (presence/absence) in species detection between 18S alone and 18S multiplexed are highlighted in grey. Pearson correlation r = 0.965, R^2^ = 0.931.

| **Phylum/Subphylum** | **Order** | **Genus/Family** | **Species** | 18S alone | 18S multiplexed |
| --- | --- | --- | --- | --- | --- |
| Crustacea | Amphipoda | *Crangonyx* | *Crangonyx* | 3 | 46 |
|  | Amphipoda | *Gammarus* | *Gammarus lacustris* | 19 | 5785 |
|  | Amphipoda | *Gammarus* | *Gammarus lawrencianus* | 0 | 0 |
|  | Amphipoda | *Gammarus* | *Gammarus oceanicus* | 1 | 0 |
|  | Amphipoda | *Hyalella* | *Hyalella azteca* | 0 | 0 |
|  | Amphipoda | *Hyalella* | *Hyalella* clade 1 | 4 | 0 |
|  | Amphipoda | *Hyalella* | *Hyalella* clade 8 | 4710 | 8259 |
|  | Amphipoda | *Hyperia* | *Hyperia galba* | 0 | 0 |
|  | Amphipoda | *Hyperoche* | *Hyperoche mediterranea* | 0 | 0 |
|  | Amphipoda | *Themisto* | *Themisto libellula* | 7257 | 9322 |
|  | Anostraca | *Artemia* | *Artemia spp* | 44983 | 48709 |
|  | Calanoida | *Calanus* | *Calanus finmarchicus* | 16943 | 115 |
|  | Calanoida | *Centropages* | *Centropages abdominalis* | 46425 | 39936 |
|  | Calanoida | *Eurytemora* | *Eurytemora affinis* | 10009 | 12309 |
|  | Calanoida | *Leptodiaptomus* | *Leptodiaptomus minutus* | 4637 | 5535 |
|  | Calanoida | *Limnocalanus* | *Limnocalanus macrurus* | 0 | 0 |
|  | Calanoida | *Pseudocalanus* | *Pseudocalanus mimus* | 0 | 0 |
|  | Cyclopoida | *Acanthocyclops* | *Acanthocyclops vernalis* | 0 | 0 |
|  | Cyclopoida | *Eucyclops* | *Eucyclops speratus* | 5306 | 7905 |
|  | Cyclopoida | *Macrocyclops* | *Macrocyclops albidus* | 6320 | 8719 |
|  | Cyclopoida | *Oithona* | *Oithona atlantica* | 19 | 8 |
|  | Decapoda | *Carcinus* | *Carcinus maenas* | 1257 | 7 |
|  | Decapoda | *Caridean* | *Caridean larvae* | 7883 | 17169 |
|  | Decapoda | Crangonidae | *Crangonidae* | 46012 | 51560 |
|  | Decapoda | Grapsidae | *Grapsidae* | 0 | 0 |
|  | Decapoda | Hippolytidae | *Hippolytidae* | 0 | 0 |
|  | Decapoda | Majidae | *Majidae* | 0 | 0 |
|  | Decapoda | *Neotrypaea* | *Neotrypaea californiensis* | 41745 | 50011 |
|  | Decapoda | Xanthidae | *Xanthidae* | 0 | 0 |
|  | Diplostraca | *Bosmina* | *Bosmina longirostris* | 1364 | 2383 |
|  | Diplostraca | *Bythotrephes* | *Bythotrephes longimanus* | 0 | 0 |
|  | Diplostraca | *Ceriodaphnia* | *Ceriodaphnia lacustris* | 2 | 8 |
|  | Diplostraca | *Daphnia* | *Daphnia parvula* | 0 | 0 |
|  | Diplostraca | *Daphnia* | *Daphnia pulex* | 19918 | 30247 |
|  | Diplostraca | *Daphnia* | *Daphnia pulicaria* | 0 | 0 |
|  | Diplostraca | *Diaphanosoma* | *Diaphanosoma brachyurum* | 48 | 26 |
|  | Diplostraca | *Holopedium* | *Holopedium gibberum* | 0 | 1 |
|  | Diplostraca | *Leptodora* | *Leptodora kindtii* | 48 | 1 |
|  | Diplostraca | *Polyphemus* | *Polyphemus pediculus* | 1693 | 7113 |
|  | Harpacticoida | *Clytemnestra* | *Clytemnestra scutellata* | 0 | 0 |
|  | Harpacticoida | *Tachidiidae* | *Tachidiidae* | 0 | 0 |
|  | Harpacticoida | *Tisbe* | *Tisbe furcata* | 1265 | 1707 |
|  | Harpacticoida | *Zaus* | *Zaus abbreviatus* | 2031 | 3402 |
|  | Sessilia | *Balanus* | *Balanus crenatus* | 27 | 5 |
|  | Sessilia | *Balanus* | *Balanus glandula* | 85431 | 70521 |
|  | Sessilia | *Chthamalus* | *Chthamalus dalli* | 2998 | 3621 |
| Mollusca | Anaspidea | *Pteropoda* | *Pteropoda* | 0 | 0 |
|  | Cycloneritimorpha | *Nerita* | *Nerita spp* | 1258 | 4137 |
|  | Myida | *Dreissena* | *Dreissena polymorpha or rostriformis bugensis* | 0 | 0 |
|  | Mytilida | *Limnoperna* | *Limnoperna fortunei* | 1425 | 3051 |
|  | Mytilida | *Mytilus* | *Mytilus edulis* | 2739 | 6523 |
|  | Neogastropoda | *Nassarius* | *Nassarius distortus* | 594 | 3291 |
|  | Thecosomata | *Limacina* | *Limacina helicina* | 0 | 0 |
|  | Venerida | *Corbicula* | *Corbicula fluminea* | 0 | 2 |
| Tunicata | Copelata | *Oikopleura* | *Oikopleura labradonensis* | 0 | 11 |
|  | Phlebobranchia | *Ciona* | *Ciona intestinalis* | 8 | 0 |
| Average number of reads per individual or species (n=56) | | | | 6507 | 7169 |

**Table S6**: Detailed number of reads of all the species for 18S and 3 COI fragments across 24 libraries. The column ‘n’ refers to the number of individuals.

| **Library** | **Order** | **Genus/Family** | **Species** | **n** | **18S** | **FC** | **Leray** | **Folmer** |
| --- | --- | --- | --- | --- | --- | --- | --- | --- |
| 1a | Cyclopoida | *Acanthocyclops* | *Acanthocyclops vernalis* | 1 | 0 | 0 | 0 | 0 |
| 1a | Diplostraca | *Bosmina* | *Bosmina longirostris* | 1 | 2383 | 0 | 0 | 0 |
| 1a | Diplostraca | *Ceriodaphnia* | *Ceriodaphnia lacustris* | 1 | 8 | 12 | 12803 | 64 |
| 1a | Diplostraca | *Daphnia* | *Daphnia parvula* | 1 | 0 | 0 | 0 | 0 |
| 1a | Diplostraca | *Daphnia* | *Daphnia pulex* | 1 | 30247 | 10 | 163 | 5963 |
| 1a | Diplostraca | *Daphnia* | *Daphnia pulicaria* | 1 | 0 | 0 | 0 | 0 |
| 1a | Diplostraca | *Holopedium* | *Holopedium gibberum* | 1 | 1 | 555 | 92809 | 161 |
| 1a | Diplostraca | *Leptodora* | *Leptodora kindtii* | 1 | 1 | 2899 | 30377 | 4814 |
| 1a | Diplostraca | *Polyphemus* | *Polyphemus pediculus* | 1 | 7113 | 509 | 15253 | 924 |
| 1a | Calanoida | *Pseudocalanus* | *Pseudocalanus mimus* | 1 | 0 | 0 | 0 | 0 |
| 1b | Anostraca | *Artemia* | *Artemia spp* | 1 | 48709 | 341 | 97932 | 475 |
| 1b | Phlebobranchia | *Ciona* | *Ciona intestinalis* | 1 | 0 | 0 | 0 | 0 |
| 1b | Venerida | *Corbicula* | *Corbicula fluminea* | 1 | 2 | 9935 | 46 | 11205 |
| 1b | Amphipoda | *Crangonyx* | *Crangonyx* | 1 | 46 | 0 | 0 | 0 |
| 1b | Myida | *Dreissena* | *Dreissena polymorpha or rostriformis bugensis* | 1 | 0 | 0 | 0 | 0 |
| 1b | Calanoida | *Eurytemora* | *Eurytemora affinis* | 1 | 12309 | 4480 | 121711 | 1263 |
| 1b | Amphipoda | *Gammarus* | *Gammarus lacustris* | 1 | 5785 | 0 | 0 | 0 |
| 1b | Amphipoda | *Gammarus* | *Gammarus lawrencianus* | 1 | 0 | 0 | 0 | 0 |
| 1b | Amphipoda | *Gammarus* | *Gammarus oceanicus* | 1 | 0 | 37145 | 9671 | 2617 |
| 1b | Amphipoda | *Hyalella* | *Hyalella azteca* | 1 | 0 | 50 | 905 | 2336 |
| 1b | Amphipoda | *Hyalella* | *Hyalella* clade 1 | 1 | 0 | 0 | 0 | 0 |
| 1b | Amphipoda | *Hyalella* | *Hyalella* clade 8 | 1 | 8259 | 0 | 0 | 0 |
| 1b | Amphipoda | *Hyperia* | *Hyperia galba* | 1 | 0 | 0 | 4 | 1 |
| 1b | Amphipoda | *Hyperoche* | *Hyperoche mediterranea* | 1 | 0 | 0 | 0 | 0 |
| 1b | Mytilida | *Limnoperna* | *Limnoperna fortunei* | 1 | 3051 | 3320 | 6966 | 4139 |
| 1b | Amphipoda | *Themisto* | *Themisto libellula* | 1 | 9322 | 0 | 0 | 0 |
| 1c | Sessilia | *Balanus* | *Balanus crenatus* | 1 | 5 | 3 | 419 | 3 |
| 1c | Sessilia | *Balanus* | *Balanus glandula* | 1 | 70521 | 14483 | 11278 | 16675 |
| 1c | Decapoda | *Carcinus* | *Carcinus maenas* | 1 | 7 | 22 | 1 | 18 |
| 1c | Decapoda | *Caridean* | *Caridean larvae* | 1 | 17169 | 0 | 0 | 0 |
| 1c | Sessilia | *Chthamalus* | *Chthamalus dalli* | 1 | 3621 | 32066 | 1713 | 9452 |
| 1c | Decapoda | Crangonidae | *Crangonidae* | 1 | 51560 | 0 | 0 | 0 |
| 1c | Decapoda | Grapsidae | *Grapsidae* | 1 | 0 | 0 | 0 | 0 |
| 1c | Decapoda | Hippolytidae | *Hippolytidae* | 1 | 0 | 0 | 0 | 0 |
| 1c | Thecosomata | *Limacina* | *Limacina helicina* | 1 | 0 | 18 | 14 | 1223 |
| 1c | Decapoda | Majidae | *Majidae* | 1 | 0 | 0 | 0 | 0 |
| 1c | Mytilida | *Mytilus* | *Mytilus edulis* | 1 | 6523 | 0 | 62 | 9 |
| 1c | Neogastropoda | *Nassarius* | *Nassarius distortus* | 1 | 3291 | 0 | 0 | 0 |
| 1c | Cycloneritimorpha | *Nerita* | *Nerita spp* | 1 | 4137 | 7 | 31 | 1624 |
| 1c | Decapoda | Xanthidae | *Xanthidae* | 1 | 0 | 0 | 0 | 0 |
| 1d | Diplostraca | *Bythotrephes* | *Bythotrephes longimanus* | 1 | 0 | 9 | 1966 | 4 |
| 1d | Calanoida | *Calanus* | *Calanus finmarchicus* | 1 | 115 | 0 | 0 | 0 |
| 1d | Calanoida | *Centropages* | *Centropages abdominalis* | 1 | 39936 | 367 | 1 | 5 |
| 1d | Harpacticoida | *Clytemnestra* | *Clytemnestra scutellata* | 1 | 0 | 0 | 0 | 0 |
| 1d | Diplostraca | *Diaphanosoma* | *Diaphanosoma brachyurum* | 1 | 26 | 0 | 0 | 0 |
| 1d | Cyclopoida | *Eucyclops* | *Eucyclops speratus* | 1 | 7905 | 0 | 0 | 0 |
| 1d | Calanoida | *Leptodiaptomus* | *Leptodiaptomus minutus* | 1 | 5535 | 12671 | 9232 | 195 |
| 1d | Calanoida | *Limnocalanus* | *Limnocalanus macrurus* | 1 | 0 | 0 | 0 | 0 |
| 1d | Cyclopoida | *Macrocyclops* | *Macrocyclops albidus* | 1 | 8719 | 0 | 0 | 0 |
| 1d | Decapoda | *Neotrypaea* | *Neotrypaea californiensis* | 1 | 50011 | 0 | 0 | 0 |
| 1d | Copelata | *Oikopleura* | *Oikopleura labradonensis* | 1 | 11 | 0 | 0 | 0 |
| 1d | Cyclopoida | *Oithona* | *Oithona atlantica* | 1 | 8 | 0 | 0 | 0 |
| 1d | Anaspidea | *Pteropoda* | *Pteropoda* | 1 | 0 | 0 | 0 | 0 |
| 1d | Harpacticoida | *Tachidiidae* | *Tachidiidae* | 1 | 0 | 0 | 0 | 0 |
| 1d | Harpacticoida | *Tisbe* | *Tisbe furcata* | 1 | 1707 | 0 | 2358 | 1 |
| 1d | Harpacticoida | *Zaus* | *Zaus abbreviatus* | 1 | 3402 | 0 | 0 | 0 |
| 1e | Cyclopoida | *Acanthocyclops* | *Acanthocyclops vernalis* | 1 | 0 | 0 | 0 | 0 |
| 1e | Anostraca | *Artemia* | *Artemia spp* | 1 | 17044 | 21 | 23988 | 151 |
| 1e | Sessilia | *Balanus* | *Balanus crenatus* | 1 | 12 | 22 | 69 | 5 |
| 1e | Sessilia | *Balanus* | *Balanus glandula* | 1 | 46040 | 11466 | 4662 | 18818 |
| 1e | Diplostraca | *Bosmina* | *Bosmina longirostris* | 1 | 48 | 0 | 0 | 0 |
| 1e | Diplostraca | *Bythotrephes* | *Bythotrephes longimanus* | 1 | 0 | 1 | 299 | 0 |
| 1e | Calanoida | *Calanus* | *Calanus finmarchicus* | 1 | 9 | 0 | 0 | 0 |
| 1e | Decapoda | *Carcinus* | *Carcinus maenas* | 1 | 10 | 21 | 0 | 13 |
| 1e | Decapoda | *Caridean* | *Caridean larvae* | 1 | 10548 | 0 | 0 | 0 |
| 1e | Calanoida | *Centropages* | *Centropages abdominalis* | 1 | 6796 | 5 | 0 | 0 |
| 1e | Diplostraca | *Ceriodaphnia* | *Ceriodaphnia lacustris* | 1 | 0 | 0 | 17 | 0 |
| 1e | Sessilia | *Chthamalus* | *Chthamalus dalli* | 1 | 2258 | 24727 | 700 | 9075 |
| 1e | Phlebobranchia | *Ciona* | *Ciona intestinalis* | 1 | 0 | 0 | 0 | 0 |
| 1e | Harpacticoida | *Clytemnestra* | *Clytemnestra scutellata* | 1 | 0 | 0 | 0 | 0 |
| 1e | Venerida | *Corbicula* | *Corbicula fluminea* | 1 | 0 | 714 | 6 | 4670 |
| 1e | Decapoda | Crangonidae | *Crangonidae* | 1 | 34549 | 0 | 0 | 0 |
| 1e | Amphipoda | *Crangonyx* | *Crangonyx* | 1 | 5 | 0 | 0 | 0 |
| 1e | Diplostraca | *Daphnia* | *Daphnia parvula* | 1 | 0 | 0 | 0 | 0 |
| 1e | Diplostraca | *Daphnia* | *Daphnia pulex* | 1 | 917 | 0 | 0 | 9 |
| 1e | Diplostraca | *Daphnia* | *Daphnia pulicaria* | 1 | 0 | 0 | 0 | 0 |
| 1e | Diplostraca | *Diaphanosoma* | *Diaphanosoma brachyurum* | 1 | 10 | 0 | 0 | 0 |
| 1e | Myida | *Dreissena* | *Dreissena polymorpha or rostriformis bugensis* | 1 | 0 | 0 | 0 | 0 |
| 1e | Cyclopoida | *Eucyclops* | *Eucyclops speratus* | 1 | 942 | 0 | 0 | 0 |
| 1e | Calanoida | *Eurytemora* | *Eurytemora affinis* | 1 | 4107 | 333 | 41692 | 527 |
| 1e | Amphipoda | *Gammarus* | *Gammarus lacustris* | 1 | 2350 | 0 | 0 | 0 |
| 1e | Amphipoda | *Gammarus* | *Gammarus lawrencianus* | 1 | 0 | 0 | 0 | 0 |
| 1e | Amphipoda | *Gammarus* | *Gammarus oceanicus* | 1 | 0 | 3513 | 1374 | 768 |
| 1e | Decapoda | Grapsidae | *Grapsidae* | 1 | 0 | 0 | 0 | 0 |
| 1e | Decapoda | Hippolytidae | *Hippolytidae* | 1 | 0 | 0 | 0 | 0 |
| 1e | Diplostraca | *Holopedium* | *Holopedium gibberum* | 1 | 0 | 7 | 1859 | 2 |
| 1e | Amphipoda | *Hyalella* | *Hyalella azteca* | 1 | 0 | 5 | 186 | 888 |
| 1e | Amphipoda | *Hyalella* | *Hyalella* clade 1 | 1 | 0 | 0 | 0 | 0 |
| 1e | Amphipoda | *Hyalella* | *Hyalella* clade 8 | 1 | 3448 | 0 | 0 | 0 |
| 1e | Amphipoda | *Hyperia* | *Hyperia galba* | 1 | 0 | 1 | 1 | 1 |
| 1e | Amphipoda | *Hyperoche* | *Hyperoche mediterranea* | 1 | 0 | 0 | 0 | 0 |
| 1e | Calanoida | *Leptodiaptomus* | *Leptodiaptomus minutus* | 1 | 951 | 406 | 1967 | 7 |
| 1e | Diplostraca | *Leptodora* | *Leptodora kindtii* | 1 | 0 | 34 | 150 | 13 |
| 1e | Thecosomata | *Limacina* | *Limacina helicina* | 1 | 0 | 23 | 7 | 1055 |
| 1e | Calanoida | *Limnocalanus* | *Limnocalanus macrurus* | 1 | 0 | 0 | 0 | 0 |
| 1e | Mytilida | *Limnoperna* | *Limnoperna fortunei* | 1 | 872 | 215 | 1048 | 1324 |
| 1e | Cyclopoida | *Macrocyclops* | *Macrocyclops albidus* | 1 | 1131 | 0 | 0 | 0 |
| 1e | Decapoda | Majidae | *Majidae* | 1 | 0 | 0 | 0 | 0 |
| 1e | Mytilida | *Mytilus* | *Mytilus edulis* | 1 | 3936 | 0 | 73 | 2 |
| 1e | Neogastropoda | *Nassarius* | *Nassarius distortus* | 1 | 1533 | 0 | 0 | 0 |
| 1e | Decapoda | *Neotrypaea* | *Neotrypaea californiensis* | 1 | 5688 | 0 | 0 | 0 |
| 1e | Cycloneritimorpha | *Nerita* | *Nerita spp* | 1 | 1673 | 10 | 26 | 1125 |
| 1e | Copelata | *Oikopleura* | *Oikopleura labradonensis* | 1 | 3 | 0 | 0 | 0 |
| 1e | Cyclopoida | *Oithona* | *Oithona atlantica* | 1 | 1 | 0 | 0 | 0 |
| 1e | Diplostraca | *Polyphemus* | *Polyphemus pediculus* | 1 | 111 | 4 | 89 | 0 |
| 1e | Calanoida | *Pseudocalanus* | *Pseudocalanus mimus* | 1 | 0 | 0 | 0 | 0 |
| 1e | Anaspidea | *Pteropoda* | *Pteropoda* | 1 | 4 | 0 | 0 | 0 |
| 1e | Harpacticoida | *Tachidiidae* | *Tachidiidae* | 1 | 0 | 0 | 0 | 0 |
| 1e | Amphipoda | *Themisto* | *Themisto libellula* | 1 | 2743 | 0 | 0 | 0 |
| 1e | Harpacticoida | *Tisbe* | *Tisbe furcata* | 1 | 240 | 0 | 436 | 0 |
| 1e | Decapoda | Xanthidae | *Xanthidae* | 1 | 0 | 0 | 0 | 0 |
| 1e | Harpacticoida | *Zaus* | *Zaus abbreviatus* | 1 | 447 | 0 | 0 | 0 |
| 1g | Calanoida | Acartia | Acartia hudsonica | 1 | 2 | 0 | 145 | 0 |
| 1g | Trachymedusae | Aglantha | Aglantha digitale | 1 | 2413 | 1 | 1 | 8 |
| 1g | Anostraca | Artemia | Artemia spp | 1 | 3009 | 8 | 3457 | 6 |
| 1g | Isopoda | Asellus | Asellus | 1 | 44 | 0 | 0 | 0 |
| 1g | Sessilia | Balanus | Balanus crenatus | 1 | 16 | 79 | 1149 | 14 |
| 1g | Stolidobranchia | Botrylloides | Botrylloides violaceus | 1 | 0 | 33 | 0 | 31 |
| 1g | Stolidobranchia | Botryllus | Botryllus schlosseri | 1 | 0 | 4272 | 2 | 0 |
| 1g | Ploima | Brachionus | Brachionus calyciflorus | 1 | 0 | 8 | 0 | 1 |
| 1g | Diplostraca | Bythotrephes | Bythotrephes longimanus | 1 | 0 | 0 | 4 | 0 |
| 1g | Amphipoda | Caprella | Caprella mutica | 1 | 4 | 0 | 170 | 1 |
| 1g | Diplostraca | Cercopagis | Cercopagis pengoi | 1 | 2740 | 19 | 163 | 2109 |
| 1g | Venerida | Corbicula | Corbicula fluminea | 1 | 0 | 897 | 2 | 703 |
| 1g | Ostreida | Crassostrea | Crassostrea gigas | 1 | 8083 | 29 | 1627 | 1338 |
| 1g | Diplostraca | Daphnia | Daphnia magna | 1 | 1712 | 995 | 108848 | 1614 |
| 1g | Decapoda | Hemigrapsus | Hemigrapsus oregonensis | 1 | 0 | 13172 | 22895 | 1782 |
| 1g | Mysida | Hemimysis | Hemimysis anomala | 1 | 344 | 21 | 1 | 7 |
| 1g | Amphipoda | Hyalella | Hyalella azteca | 1 | 0 | 0 | 0 | 0 |
| 1g | Ploima | Keratella | Keratella quadrata | 1 | 8 | 0 | 0 | 0 |
| 1g | Thecosomata | Limacina | Limacina helicina | 1 | 0 | 80 | 9 | 1314 |
| 1g | Mytilida | Limnoperna | Limnoperna fortunei | 1 | 75881 | 771 | 869 | 924 |
| 1g | Decapoda | Lophopanopeus | Lophopanopeus bellus | 1 | 4840 | 28915 | 72878 | 2654 |
| 1g | Cardiida | Macoma | Macoma secta | 1 | 2797 | 0 | 0 | 0 |
| 1g | Cyclopoida | Mesocyclops | Mesocyclops edax | 1 | 0 | 0 | 0 | 0 |
| 1g | Decapoda | Metacarcinus | Metacarcinus magister | 1 | 0 | 500 | 351 | 943 |
| 1g | Harpacticoida | Microsetella | Microsetella norvegica | 1 | 22 | 0 | 0 | 0 |
| 1g | Stolidobranchia | Styela | Styela clava | 1 | 321 | 18 | 1 | 973 |
| 1g | Calanoida | Tortanus | Tortanus discaudatus | 1 | 807 | 48 | 173 | 14 |
| 2a | Sessilia | *Balanus* | *Balanus crenatus* | 10 | 77705 | 78621 | 304921 | 33971 |
| 2a | Amphipoda | *Gammarus* | *Gammarus lawrencianus* | 1 | 0 | 251 | 223 | 111 |
| 2a | Calanoida | *Leptodiaptomus* | *Leptodiaptomus minutus* | 1 | 10281 | 1066 | 713 | 21 |
| 2a | Decapoda | *Palaemonetes* | *Palaemonetes spp* | 5 | 120748 | 0 | 0 | 0 |
| 2b | Calanoida | *Acartia* | *Acartia longiremis* | 5 | 0 | 0 | 0 | 0 |
| 2b | Anostraca | *Artemia* | *Artemia spp* | 2 | 82148 | 285 | 136249 | 723 |
| 2b | Sessilia | *Chthamalus* | *Chthamalus dalli* | 1 | 22117 | 87360 | 9683 | 19204 |
| 2b | Amphipoda | *Hyalella* | *Hyalella* clade 8 | 5 | 58960 | 0 | 0 | 0 |
| 2b | Diplostraca | *Leptodora* | *Leptodora kindtii* | 5 | 0 | 4270 | 31645 | 1130 |
| 2c | Decapoda | *Carcinus* | *Carcinus maenas* | 2 | 168 | 2887 | 5 | 57 |
| 2c | Venerida | *Corbicula* | *Corbicula fluminea* | 5 | 9 | 118847 | 855 | 65196 |
| 2c | Diplostraca | *Daphnia* | *Daphnia pulex* | 10 | 65503 | 44 | 3617 | 1027 |
| 2c | Cycloneritimorpha | *Nerita* | *Nerita spp* | 1 | 31272 | 1370 | 9301 | 20703 |
| 2d | Calanoida | *Eurytemora* | *Eurytemora affinis* | 23 | 75980 | 55610 | 220231 | 31413 |
| 2e | Calanoida | *Acartia* | *Acartia longiremis* | 5 | 0 | 0 | 0 | 0 |
| 2e | Anostraca | *Artemia* | *Artemia spp* | 2 | 14504 | 15 | 1169 | 124 |
| 2e | Sessilia | *Balanus* | *Balanus crenatus* | 10 | 34321 | 46910 | 196638 | 30798 |
| 2e | Decapoda | *Carcinus* | *Carcinus maenas* | 2 | 7 | 8 | 0 | 3 |
| 2e | Sessilia | *Chthamalus* | *Chthamalus dalli* | 1 | 1754 | 10880 | 89 | 5254 |
| 2e | Venerida | *Corbicula* | *Corbicula fluminea* | 5 | 0 | 561 | 9 | 3341 |
| 2e | Diplostraca | *Daphnia* | *Daphnia pulex* | 10 | 3682 | 0 | 3 | 23 |
| 2e | Calanoida | *Eurytemora* | *Eurytemora affinis* | 23 | 30051 | 7415 | 74084 | 9078 |
| 2e | Amphipoda | *Gammarus* | *Gammarus lawrencianus* | 1 | 0 | 99 | 110 | 54 |
| 2e | Amphipoda | *Hyalella* | *Hyalella* clade 8 | 5 | 17518 | 0 | 0 | 0 |
| 2e | Calanoida | *Leptodiaptomus* | *Leptodiaptomus minutus* | 1 | 1509 | 496 | 237 | 8 |
| 2e | Diplostraca | *Leptodora* | *Leptodora kindtii* | 5 | 0 | 298 | 157 | 94 |
| 2e | Cycloneritimorpha | *Nerita* | *Nerita spp* | 1 | 1081 | 2 | 7 | 511 |
| 2e | Decapoda | *Palaemonetes* | *Palaemonetes spp* | 5 | 54207 | 0 | 0 | 0 |
| 2g | Calanoida | *Acartia* | *Acartia hudsonica* | 4 | 4 | 0 | 217 | 0 |
| 2g | Anostraca | *Artemia* | *Artemia spp* | 2 | 3019 | 15 | 1510 | 19 |
| 2g | Isopoda | *Asellus* | *Asellus* | 1 | 1 | 0 | 0 | 0 |
| 2g | Sessilia | *Balanus* | *Balanus crenatus* | 3 | 367 | 2491 | 21246 | 299 |
| 2g | Stolidobranchia | *Botrylloides* | *Botrylloides violaceus* | 2 | 0 | 8 | 0 | 15 |
| 2g | Stolidobranchia | *Botryllus* | *Botryllus schlosseri* | 1 | 0 | 17393 | 2 | 20 |
| 2g | Ploima | *Brachionus* | *Brachionus calyciflorus* | 5 | 15 | 8 | 0 | 5 |
| 2g | Diplostraca | *Bythotrephes* | *Bythotrephes longimanus* | 2 | 0 | 0 | 0 | 0 |
| 2g | Amphipoda | *Caprella* | *Caprella mutica* | 1 | 3 | 1 | 568 | 3 |
| 2g | Diplostraca | *Cercopagis* | *Cercopagis pengoi* | 3 | 3756 | 23 | 146 | 1566 |
| 2g | Venerida | *Corbicula* | *Corbicula fluminea* | 2 | 1 | 3339 | 6 | 2738 |
| 2g | Ostreida | *Crassostrea* | *Crassostrea gigas* | 2 | 13453 | 49 | 2693 | 1939 |
| 2g | Diplostraca | *Daphnia* | *Daphnia magna* | 3 | 2853 | 1204 | 132843 | 2264 |
| 2g | Decapoda | *Hemigrapsus* | *Hemigrapsus oregonensis* | 2 | 0 | 4715 | 8063 | 595 |
| 2g | Mysida | *Hemimysis* | *Hemimysis anomala* | 1 | 135 | 4 | 0 | 1 |
| 2g | Amphipoda | *Hyalella* | *Hyalella azteca* | 3 | 0 | 0 | 0 | 0 |
| 2g | Ploima | *Keratella* | *Keratella quadrata* | 5 | 5 | 0 | 0 | 4 |
| 2g | Thecosomata | *Limacina* | *Limacina helicina* | 1 | 0 | 116 | 23 | 2637 |
| 2g | Mytilida | *Limnoperna* | *Limnoperna fortunei* | 3 | 53593 | 1451 | 1526 | 1272 |
| 2g | Decapoda | *Lophopanopeus* | *Lophopanopeus bellus* | 2 | 3862 | 27036 | 72664 | 2533 |
| 2g | Cardiida | *Macoma* | *Macoma secta* | 1 | 1867 | 0 | 0 | 0 |
| 2g | Cyclopoida | *Mesocyclops* | *Mesocyclops edax* | 4 | 0 | 0 | 0 | 0 |
| 2g | Decapoda | *Metacarcinus* | *Metacarcinus magister* | 1 | 0 | 598 | 466 | 1286 |
| 2g | Harpacticoida | *Microsetella* | *Microsetella norvegica* | 3 | 11 | 0 | 0 | 0 |
| 2g | Stolidobranchia | *Styela* | *Styela clava* | 1 | 284 | 18 | 0 | 1848 |
| 2g | Calanoida | *Tortanus* | *Tortanus discaudatus* | 4 | 1463 | 121 | 527 | 43 |
| 3a1 | Mytilida | *Limnoperna* | *Limnoperna fortunei* | 1 | 105955 | 14326 | 149790 | 16161 |
| 3a2 | Mytilida | *Limnoperna* | *Limnoperna fortunei* | 10 | 165347 | 14031 | 153880 | 10453 |
| 3a3 | Mytilida | *Limnoperna* | *Limnoperna fortunei* | 30 | 207171 | 23678 | 237670 | 18006 |
| 3b1 | Sessilia | *Balanus* | *Balanus crenatus* | 1 | 49784 | 87106 | 228805 | 4644 |
| 3b2 | Sessilia | *Balanus* | *Balanus crenatus* | 10 | 42001 | 73379 | 215991 | 1923 |
| 3b3 | Sessilia | *Balanus* | *Balanus crenatus* | 17 | 39405 | 7412 | 198797 | 2 |
| 3c1 | Calanoida | *Tortanus* | *Tortanus discaudatus* | 1 | 71308 | 36404 | 318338 | 19419 |
| 3c2 | Calanoida | *Tortanus* | *Tortanus discaudatus* | 8 | 86973 | 35889 | 184408 | 30596 |
| 3c3 | Calanoida | *Tortanus* | *Tortanus discaudatus* | 15 | 75067 | 60966 | 345532 | 24873 |
| 3d1 | Diplostraca | *Leptodora* | *Leptodora kindtii* | 1 | 10 | 104481 | 294868 | 9954 |
| 3d2 | Diplostraca | *Leptodora* | *Leptodora kindtii* | 10 | 8 | 167544 | 301656 | 16348 |
| 3d3 | Diplostraca | *Leptodora* | *Leptodora kindtii* | 28 | 8 | 139830 | 164686 | 18336 |

**Table S7**: Number of reads assigned to the species level in the local database. Highlighted in grey are the species assembled in the mock communities; species with “NA” are species not intentionally included in the communities and considered contaminants (parasites, gut content or cross contamination).

| **Library** | **Species** | **n** | **18S** | **FC** | **Leray** | **Folmer** | **Total** |
| --- | --- | --- | --- | --- | --- | --- | --- |
| 1a | *Bosmina longirostris* | 1 | 2383 | 0 | 0 | 0 | 2383 |
| 1a | *Ceriodaphnia lacustris* | 1 | 8 | 12 | 12803 | 64 | 12887 |
| 1a | *Daphnia pulex* | 1 | 30247 | 10 | 163 | 5963 | 36383 |
| 1a | *Holopedium gibberum* | 1 | 1 | 555 | 92809 | 161 | 93526 |
| 1a | *Leptodora kindtii* | 1 | 1 | 2899 | 30377 | 4814 | 38091 |
| 1a | *Polyphemus pediculus* | 1 | 7113 | 509 | 15253 | 924 | 23799 |
| 1a | *Aglantha digitale* | NA | 1 | 0 | 0 | 0 | 1 |
| 1a | *Artemia spp* | NA | 8 | 0 | 9 | 0 | 17 |
| 1a | *Balanus crenatus* | NA | 2 | 2 | 16 | 2 | 22 |
| 1a | *Balanus glandula* | NA | 26 | 8 | 3 | 4 | 41 |
| 1a | *Bythotrephes longimanus* | NA | 0 | 0 | 1 | 0 | 1 |
| 1a | *Caridean larvae* | NA | 2 | 0 | 0 | 0 | 2 |
| 1a | *Centropages abdominalis* | NA | 8 | 0 | 0 | 0 | 8 |
| 1a | *Cercopagis pengoi* | NA | 0 | 0 | 0 | 1 | 1 |
| 1a | *Chthamalus dalli* | NA | 0 | 16 | 1 | 1 | 18 |
| 1a | *Corbicula fluminea* | NA | 0 | 22 | 0 | 3 | 25 |
| 1a | *Crangonidae* | NA | 16 | 0 | 0 | 0 | 16 |
| 1a | *Crassostrea gigas* | NA | 3 | 0 | 0 | 0 | 3 |
| 1a | *Daphnia magna* | NA | 7 | 0 | 40 | 1 | 48 |
| 1a | *Eucyclops speratus* | NA | 1 | 0 | 0 | 0 | 1 |
| 1a | *Eurytemora affinis* | NA | 10 | 0 | 27 | 0 | 37 |
| 1a | *Gammarus oceanicus* | NA | 0 | 4 | 2 | 1 | 7 |
| 1a | *Hemigrapsus oregonensis* | NA | 0 | 17 | 76 | 2 | 95 |
| 1a | *Leptodiaptomus minutus* | NA | 4 | 2 | 0 | 0 | 6 |
| 1a | *Limnoperna fortunei* | NA | 35 | 1 | 12 | 2 | 50 |
| 1a | *Lophopanopeus bellus* | NA | 4 | 3 | 25 | 0 | 32 |
| 1a | *Mytilus edulis* | NA | 2 | 0 | 0 | 0 | 2 |
| 1a | *Nassarius distortus* | NA | 1 | 0 | 0 | 0 | 1 |
| 1a | *Neotrypaea californiensis* | NA | 9 | 0 | 0 | 0 | 9 |
| 1a | *Nerita spp* | NA | 4 | 0 | 0 | 0 | 4 |
| 1a | *Palaemonetes spp* | NA | 18 | 0 | 0 | 0 | 18 |
| 1a | *Tisbe furcata* | NA | 1 | 0 | 0 | 0 | 1 |
| 1a | *Tortanus discaudatus* | NA | 8 | 2 | 9 | 2 | 21 |
| 1b | *Artemia spp* | 1 | 48709 | 341 | 97932 | 475 | 147457 |
| 1b | *Corbicula fluminea* | 1 | 2 | 9935 | 46 | 11205 | 21188 |
| 1b | *Crangonyx* | 1 | 46 | 0 | 0 | 0 | 46 |
| 1b | *Eurytemora affinis* | 1 | 12309 | 4480 | 121711 | 1263 | 139763 |
| 1b | *Gammarus lacustris* | 1 | 5785 | 0 | 0 | 0 | 5785 |
| 1b | *Gammarus oceanicus* | 1 | 0 | 37145 | 9671 | 2617 | 49433 |
| 1b | *Hyalella azteca* | 1 | 0 | 50 | 905 | 2336 | 3291 |
| 1b | *Hyalella clade 8* | 1 | 8259 | 0 | 0 | 0 | 8259 |
| 1b | *Hyperia galba* | 1 | 0 | 0 | 4 | 1 | 5 |
| 1b | *Limnoperna fortunei* | 1 | 3051 | 3320 | 6966 | 4139 | 17476 |
| 1b | *Themisto libellula* | 1 | 9322 | 0 | 0 | 0 | 9322 |
| 1b | *Balanus crenatus* | NA | 0 | 2 | 224 | 0 | 226 |
| 1b | *Balanus glandula* | NA | 19 | 4 | 0 | 3 | 26 |
| 1b | *Botryllus schlosseri* | NA | 0 | 4 | 0 | 0 | 4 |
| 1b | *Caridean larvae* | NA | 4 | 0 | 0 | 0 | 4 |
| 1b | *Centropages abdominalis* | NA | 12 | 0 | 0 | 0 | 12 |
| 1b | *Chthamalus dalli* | NA | 6 | 29 | 1 | 4 | 40 |
| 1b | *Crangonidae* | NA | 10 | 0 | 0 | 0 | 10 |
| 1b | *Daphnia magna* | NA | 0 | 0 | 456 | 0 | 456 |
| 1b | *Daphnia pulex* | NA | 2 | 0 | 0 | 0 | 2 |
| 1b | *Diaphanosoma brachyurum* | NA | 21 | 0 | 0 | 0 | 21 |
| 1b | *Eucyclops speratus* | NA | 3 | 0 | 0 | 0 | 3 |
| 1b | *Hemigrapsus oregonensis* | NA | 0 | 10 | 66 | 1 | 77 |
| 1b | *Holopedium gibberum* | NA | 0 | 0 | 7 | 0 | 7 |
| 1b | *Leptodiaptomus minutus* | NA | 0 | 0 | 1 | 0 | 1 |
| 1b | *Leptodora kindtii* | NA | 0 | 1 | 7 | 0 | 8 |
| 1b | *Lophopanopeus bellus* | NA | 3 | 12 | 28 | 1 | 44 |
| 1b | *Macrocyclops albidus* | NA | 1 | 0 | 0 | 0 | 1 |
| 1b | *Mytilus edulis* | NA | 4307 | 0 | 0 | 0 | 4307 |
| 1b | *Neotrypaea californiensis* | NA | 6 | 0 | 0 | 0 | 6 |
| 1b | *Nerita spp* | NA | 14 | 0 | 0 | 1 | 15 |
| 1b | *Polyphemus pediculus* | NA | 0 | 0 | 2 | 0 | 2 |
| 1b | *Styela clava* | NA | 88 | 0 | 0 | 0 | 88 |
| 1b | *Tisbe furcata* | NA | 1 | 0 | 0 | 0 | 1 |
| 1b | *Tortanus discaudatus* | NA | 6 | 0 | 8 | 0 | 14 |
| 1b | *Zaus abbreviatus* | NA | 0 | 0 | 1 | 0 | 1 |
| 1c | *Balanus crenatus* | 1 | 5 | 3 | 419 | 3 | 430 |
| 1c | *Balanus glandula* | 1 | 70521 | 14483 | 11278 | 16675 | 112957 |
| 1c | *Carcinus maenas* | 1 | 7 | 22 | 1 | 18 | 48 |
| 1c | *Caridean larvae* | 1 | 17169 | 0 | 0 | 0 | 17169 |
| 1c | *Chthamalus dalli* | 1 | 3621 | 32066 | 1713 | 9452 | 46852 |
| 1c | *Crangonidae* | 1 | 51560 | 0 | 0 | 0 | 51560 |
| 1c | *Limacina helicina* | 1 | 0 | 18 | 14 | 1223 | 1255 |
| 1c | *Mytilus edulis* | 1 | 6523 | 0 | 62 | 9 | 6594 |
| 1c | *Nassarius distortus* | 1 | 3291 | 0 | 0 | 0 | 3291 |
| 1c | *Nerita spp* | 1 | 4137 | 7 | 31 | 1624 | 5799 |
| 1c | *Aglantha digitale* | NA | 2 | 0 | 0 | 0 | 2 |
| 1c | *Artemia spp* | NA | 13 | 0 | 13 | 0 | 26 |
| 1c | *Bosmina longirostris* | NA | 1 | 0 | 0 | 0 | 1 |
| 1c | *Botryllus schlosseri* | NA | 0 | 1 | 0 | 0 | 1 |
| 1c | *Bythotrephes longimanus* | NA | 0 | 0 | 1 | 0 | 1 |
| 1c | *Centropages abdominalis* | NA | 11 | 0 | 0 | 0 | 11 |
| 1c | *Cercopagis pengoi* | NA | 1 | 0 | 0 | 0 | 1 |
| 1c | *Corbicula fluminea* | NA | 0 | 4 | 0 | 6 | 10 |
| 1c | *Crassostrea gigas* | NA | 3 | 0 | 1 | 0 | 4 |
| 1c | *Daphnia magna* | NA | 1 | 0 | 13 | 0 | 14 |
| 1c | *Daphnia pulex* | NA | 9 | 0 | 0 | 0 | 9 |
| 1c | *Eucyclops speratus* | NA | 3 | 0 | 0 | 0 | 3 |
| 1c | *Eurytemora affinis* | NA | 8 | 0 | 29 | 0 | 37 |
| 1c | *Gammarus lacustris* | NA | 2 | 0 | 0 | 0 | 2 |
| 1c | *Gammarus oceanicus* | NA | 0 | 10 | 2 | 0 | 12 |
| 1c | *Hemigrapsus oregonensis* | NA | 3 | 24339 | 164685 | 2971 | 191998 |
| 1c | *Holopedium gibberum* | NA | 0 | 0 | 7 | 0 | 7 |
| 1c | *Leptodiaptomus minutus* | NA | 1 | 1 | 1 | 0 | 3 |
| 1c | *Leptodora kindtii* | NA | 0 | 0 | 2 | 0 | 2 |
| 1c | *Limnoperna fortunei* | NA | 62 | 1 | 17 | 2 | 82 |
| 1c | *Lophopanopeus bellus* | NA | 10011 | 2128 | 53099 | 211 | 65449 |
| 1c | *Macrocyclops albidus* | NA | 1 | 0 | 0 | 0 | 1 |
| 1c | *Neotrypaea californiensis* | NA | 8 | 0 | 0 | 0 | 8 |
| 1c | *Oithona atlantica* | NA | 8 | 0 | 0 | 0 | 8 |
| 1c | *Palaemonetes spp* | NA | 1 | 0 | 0 | 0 | 1 |
| 1c | *Polyphemus pediculus* | NA | 1 | 0 | 2 | 0 | 3 |
| 1c | *Pteropoda* | NA | 7 | 0 | 0 | 0 | 7 |
| 1c | *Themisto libellula* | NA | 1 | 0 | 0 | 0 | 1 |
| 1c | *Tortanus discaudatus* | NA | 7 | 1 | 24 | 1 | 33 |
| 1c | *Zaus abbreviatus* | NA | 1 | 0 | 0 | 0 | 1 |
| 1d | *Bythotrephes longimanus* | 1 | 0 | 9 | 1966 | 4 | 1979 |
| 1d | *Calanus finmarchicus* | 1 | 115 | 0 | 0 | 0 | 115 |
| 1d | *Centropages abdominalis* | 1 | 39936 | 367 | 1 | 5 | 40309 |
| 1d | *Diaphanosoma brachyurum* | 1 | 26 | 0 | 0 | 0 | 26 |
| 1d | *Eucyclops speratus* | 1 | 7905 | 0 | 0 | 0 | 7905 |
| 1d | *Leptodiaptomus minutus* | 1 | 5535 | 12671 | 9232 | 195 | 27633 |
| 1d | *Macrocyclops albidus* | 1 | 8719 | 0 | 0 | 0 | 8719 |
| 1d | *Neotrypaea californiensis* | 1 | 50011 | 0 | 0 | 0 | 50011 |
| 1d | *Oikopleura labradonensis* | 1 | 11 | 0 | 0 | 0 | 11 |
| 1d | *Oithona atlantica* | 1 | 8 | 0 | 0 | 0 | 8 |
| 1d | *Tisbe furcata* | 1 | 1707 | 0 | 2358 | 1 | 4066 |
| 1d | *Zaus abbreviatus* | 1 | 3402 | 2 | 174 | 144 | 3722 |
| 1d | *Acartia longiremis* | NA | 0 | 15 | 5 | 0 | 20 |
| 1d | *Aglantha digitale* | NA | 2 | 0 | 0 | 0 | 2 |
| 1d | *Artemia spp* | NA | 10 | 0 | 10 | 0 | 20 |
| 1d | *Balanus crenatus* | NA | 4 | 10 | 17 | 0 | 31 |
| 1d | *Balanus glandula* | NA | 23 | 6 | 2 | 4 | 35 |
| 1d | *Bosmina longirostris* | NA | 2 | 0 | 0 | 0 | 2 |
| 1d | *Caridean larvae* | NA | 3 | 0 | 0 | 0 | 3 |
| 1d | *Cercopagis pengoi* | NA | 3 | 0 | 0 | 0 | 3 |
| 1d | *Ceriodaphnia lacustris* | NA | 0 | 0 | 2 | 0 | 2 |
| 1d | *Chthamalus dalli* | NA | 0 | 17 | 1 | 2 | 20 |
| 1d | *Corbicula fluminea* | NA | 0 | 1 | 0 | 1 | 2 |
| 1d | *Crangonidae* | NA | 12 | 0 | 0 | 0 | 12 |
| 1d | *Crassostrea gigas* | NA | 3 | 0 | 0 | 0 | 3 |
| 1d | *Daphnia magna* | NA | 0 | 0 | 9 | 1 | 10 |
| 1d | *Daphnia pulex* | NA | 8 | 0 | 0 | 2 | 10 |
| 1d | *Eurytemora affinis* | NA | 6030 | 2370 | 53693 | 5017 | 67110 |
| 1d | *Gammarus lacustris* | NA | 1 | 0 | 0 | 0 | 1 |
| 1d | *Gammarus oceanicus* | NA | 0 | 3 | 2 | 0 | 5 |
| 1d | *Hemigrapsus oregonensis* | NA | 0 | 24 | 82 | 1 | 107 |
| 1d | *Holopedium gibberum* | NA | 0 | 44 | 3899 | 0 | 3943 |
| 1d | *Hyalella clade 8* | NA | 2 | 0 | 0 | 0 | 2 |
| 1d | *Leptodora kindtii* | NA | 0 | 11 | 10 | 1 | 22 |
| 1d | *Limnoperna fortunei* | NA | 10 | 0 | 0 | 0 | 10 |
| 1d | *Lophopanopeus bellus* | NA | 4 | 6 | 18 | 0 | 28 |
| 1d | *Mytilus edulis* | NA | 7 | 0 | 0 | 0 | 7 |
| 1d | *Nassarius distortus* | NA | 2 | 0 | 0 | 0 | 2 |
| 1d | *Nerita spp* | NA | 2 | 0 | 0 | 2 | 4 |
| 1d | *Polyphemus pediculus* | NA | 1 | 0 | 2 | 0 | 3 |
| 1d | *Tortanus discaudatus* | NA | 2 | 0 | 0 | 0 | 2 |
| 1e | *Artemia spp* | 1 | 17044 | 21 | 23988 | 151 | 41204 |
| 1e | *Balanus crenatus* | 1 | 12 | 22 | 69 | 5 | 108 |
| 1e | *Balanus glandula* | 1 | 46040 | 11466 | 4662 | 18818 | 80986 |
| 1e | *Bosmina longirostris* | 1 | 48 | 0 | 0 | 0 | 48 |
| 1e | *Bythotrephes longimanus* | 1 | 0 | 1 | 299 | 0 | 300 |
| 1e | *Calanus finmarchicus* | 1 | 9 | 0 | 0 | 0 | 9 |
| 1e | *Carcinus maenas* | 1 | 10 | 21 | 0 | 13 | 44 |
| 1e | *Caridean larvae* | 1 | 10548 | 0 | 0 | 0 | 10548 |
| 1e | *Centropages abdominalis* | 1 | 6796 | 5 | 0 | 0 | 6801 |
| 1e | *Ceriodaphnia lacustris* | 1 | 0 | 0 | 17 | 0 | 17 |
| 1e | *Chthamalus dalli* | 1 | 2258 | 24727 | 700 | 9075 | 36760 |
| 1e | *Corbicula fluminea* | 1 | 0 | 714 | 6 | 4670 | 5390 |
| 1e | *Crangonidae* | 1 | 34549 | 0 | 0 | 0 | 34549 |
| 1e | *Crangonyx* | 1 | 5 | 0 | 0 | 0 | 5 |
| 1e | *Daphnia pulex* | 1 | 917 | 0 | 0 | 9 | 926 |
| 1e | *Diaphanosoma brachyurum* | 1 | 10 | 0 | 0 | 0 | 10 |
| 1e | *Eucyclops speratus* | 1 | 942 | 0 | 0 | 0 | 942 |
| 1e | *Eurytemora affinis* | 1 | 4107 | 333 | 41692 | 527 | 46659 |
| 1e | *Gammarus lacustris* | 1 | 2350 | 0 | 0 | 0 | 2350 |
| 1e | *Gammarus oceanicus* | 1 | 0 | 3513 | 1374 | 768 | 5655 |
| 1e | *Holopedium gibberum* | 1 | 0 | 7 | 1859 | 2 | 1868 |
| 1e | *Hyalella azteca* | 1 | 0 | 5 | 186 | 888 | 1079 |
| 1e | *Hyalella clade 8* | 1 | 3448 | 0 | 0 | 0 | 3448 |
| 1e | *Hyperia galba* | 1 | 0 | 1 | 1 | 1 | 3 |
| 1e | *Leptodiaptomus minutus* | 1 | 951 | 406 | 1967 | 7 | 3331 |
| 1e | *Leptodora kindtii* | 1 | 0 | 34 | 150 | 13 | 197 |
| 1e | *Limacina helicina* | 1 | 0 | 23 | 7 | 1055 | 1085 |
| 1e | *Limnoperna fortunei* | 1 | 872 | 215 | 1048 | 1324 | 3459 |
| 1e | *Macrocyclops albidus* | 1 | 1131 | 0 | 0 | 0 | 1131 |
| 1e | *Mytilus edulis* | 1 | 3936 | 0 | 73 | 2 | 4011 |
| 1e | *Nassarius distortus* | 1 | 1533 | 0 | 0 | 0 | 1533 |
| 1e | *Neotrypaea californiensis* | 1 | 5688 | 0 | 0 | 0 | 5688 |
| 1e | *Nerita spp* | 1 | 1673 | 10 | 26 | 1125 | 2834 |
| 1e | *Oikopleura labradonensis* | 1 | 3 | 0 | 0 | 0 | 3 |
| 1e | *Oithona atlantica* | 1 | 1 | 0 | 0 | 0 | 1 |
| 1e | *Polyphemus pediculus* | 1 | 111 | 4 | 89 | 0 | 204 |
| 1e | *Pteropoda* | 1 | 4 | 0 | 0 | 0 | 4 |
| 1e | *Themisto libellula* | 1 | 2743 | 0 | 0 | 0 | 2743 |
| 1e | *Tisbe furcata* | 1 | 240 | 0 | 436 | 0 | 676 |
| 1e | *Zaus abbreviatus* | 1 | 447 | 0 | 34 | 3 | 484 |
| 1e | *Crassostrea gigas* | NA | 1 | 0 | 0 | 0 | 1 |
| 1e | *Daphnia magna* | NA | 3 | 0 | 9 | 1 | 13 |
| 1e | *Hemigrapsus oregonensis* | NA | 0 | 18003 | 84487 | 2802 | 105292 |
| 1e | *Lophopanopeus bellus* | NA | 5496 | 1701 | 23941 | 261 | 31399 |
| 1e | *Palaemonetes spp* | NA | 5 | 0 | 0 | 0 | 5 |
| 1e | *Styela clava* | NA | 24 | 0 | 0 | 0 | 24 |
| 1g | *Acartia hudsonica* | 1 | 2 | 0 | 145 | 0 | 147 |
| 1g | *Aglantha digitale* | 1 | 2413 | 1 | 1 | 8 | 2423 |
| 1g | *Artemia spp* | 1 | 3009 | 8 | 3457 | 6 | 6480 |
| 1g | *Asellus* | 1 | 44 | 0 | 0 | 0 | 44 |
| 1g | *Balanus crenatus* | 1 | 16 | 79 | 1149 | 14 | 1258 |
| 1g | *Botrylloides violaceus* | 1 | 0 | 33 | 0 | 31 | 64 |
| 1g | *Botryllus schlosseri* | 1 | 0 | 4272 | 2 | 0 | 4274 |
| 1g | *Brachionus calyciflorus* | 1 | 0 | 8 | 0 | 1 | 9 |
| 1g | *Bythotrephes longimanus* | 1 | 0 | 0 | 4 | 0 | 4 |
| 1g | *Caprella mutica* | 1 | 4 | 0 | 170 | 1 | 175 |
| 1g | *Cercopagis pengoi* | 1 | 2740 | 19 | 163 | 2109 | 5031 |
| 1g | *Corbicula fluminea* | 1 | 0 | 897 | 2 | 703 | 1602 |
| 1g | *Crassostrea gigas* | 1 | 8083 | 29 | 1627 | 1338 | 11077 |
| 1g | *Daphnia magna* | 1 | 1712 | 995 | 108848 | 1614 | 113169 |
| 1g | *Hemigrapsus oregonensis* | 1 | 1 | 13172 | 22895 | 1782 | 37850 |
| 1g | *Hemimysis anomala* | 1 | 344 | 21 | 1 | 7 | 373 |
| 1g | *Keratella quadrata* | 1 | 8 | 0 | 0 | 0 | 8 |
| 1g | *Limacina helicina* | 1 | 0 | 80 | 9 | 1314 | 1403 |
| 1g | *Limnoperna fortunei* | 1 | 75881 | 771 | 869 | 924 | 78445 |
| 1g | *Lophopanopeus bellus* | 1 | 4840 | 28915 | 72878 | 2654 | 109287 |
| 1g | *Macoma secta* | 1 | 2797 | 0 | 0 | 0 | 2797 |
| 1g | *Metacarcinus magister* | 1 | 0 | 500 | 351 | 943 | 1794 |
| 1g | *Microsetella norvegica* | 1 | 22 | 0 | 0 | 0 | 22 |
| 1g | *Styela clava* | 1 | 321 | 18 | 1 | 973 | 1313 |
| 1g | *Tortanus discaudatus* | 1 | 807 | 48 | 173 | 14 | 1042 |
| 1g | *Balanus glandula* | NA | 17 | 6 | 3 | 1 | 27 |
| 1g | *Caridean larvae* | NA | 4 | 0 | 0 | 0 | 4 |
| 1g | *Centropages abdominalis* | NA | 7 | 0 | 0 | 0 | 7 |
| 1g | *Ceriodaphnia lacustris* | NA | 0 | 0 | 1 | 0 | 1 |
| 1g | *Chthamalus dalli* | NA | 0 | 10 | 2 | 1 | 13 |
| 1g | *Crangonidae* | NA | 13 | 0 | 0 | 0 | 13 |
| 1g | *Daphnia pulex* | NA | 158 | 0 | 0 | 0 | 158 |
| 1g | *Eurytemora affinis* | NA | 2 | 1 | 26 | 0 | 29 |
| 1g | *Gammarus lacustris* | NA | 1 | 0 | 0 | 0 | 1 |
| 1g | *Gammarus oceanicus* | NA | 0 | 8 | 2 | 0 | 10 |
| 1g | *Holopedium gibberum* | NA | 0 | 1 | 3 | 0 | 4 |
| 1g | *Leptodiaptomus minutus* | NA | 1 | 0 | 0 | 0 | 1 |
| 1g | *Leptodora kindtii* | NA | 0 | 6 | 6 | 5 | 17 |
| 1g | *Mytilus edulis* | NA | 2 | 0 | 3 | 1 | 6 |
| 1g | *Neotrypaea californiensis* | NA | 5 | 0 | 0 | 0 | 5 |
| 1g | *Nerita spp* | NA | 1 | 0 | 0 | 0 | 1 |
| 1g | *Polyphemus pediculus* | NA | 0 | 0 | 2 | 0 | 2 |
| 1g | *Pteropoda* | NA | 14 | 0 | 0 | 0 | 14 |
| 2a | *Gammarus lawrencianus* | 1 | 0 | 251 | 223 | 111 | 585 |
| 2a | *Leptodiaptomus minutus* | 1 | 10281 | 1066 | 713 | 21 | 12081 |
| 2a | *Palaemonetes spp* | 5 | 120748 | 0 | 0 | 0 | 120748 |
| 2a | *Balanus crenatus* | 10 | 77705 | 78621 | 304921 | 33971 | 495218 |
| 2a | *Acartia hudsonica* | NA | 0 | 0 | 1 | 0 | 1 |
| 2a | *Artemia spp* | NA | 7 | 0 | 11 | 0 | 18 |
| 2a | *Balanus glandula* | NA | 7434 | 1261 | 21 | 162 | 8878 |
| 2a | *Botryllus schlosseri* | NA | 0 | 4 | 0 | 0 | 4 |
| 2a | *Caprella mutica* | NA | 0 | 0 | 1 | 0 | 1 |
| 2a | *Carcinus maenas* | NA | 0 | 2 | 0 | 0 | 2 |
| 2a | *Cercopagis pengoi* | NA | 1 | 0 | 0 | 0 | 1 |
| 2a | *Chthamalus dalli* | NA | 36 | 5 | 1 | 4 | 46 |
| 2a | *Corbicula fluminea* | NA | 0 | 12 | 0 | 10 | 22 |
| 2a | *Crangonidae* | NA | 1 | 0 | 0 | 0 | 1 |
| 2a | *Crassostrea gigas* | NA | 2 | 0 | 0 | 0 | 2 |
| 2a | *Daphnia magna* | NA | 0 | 0 | 41 | 0 | 41 |
| 2a | *Daphnia pulex* | NA | 30 | 0 | 0 | 1 | 31 |
| 2a | *Eurytemora affinis* | NA | 46 | 5 | 36 | 2 | 89 |
| 2a | *Gammarus lacustris* | NA | 1 | 0 | 0 | 0 | 1 |
| 2a | *Hemigrapsus oregonensis* | NA | 0 | 2 | 0 | 0 | 2 |
| 2a | *Holopedium gibberum* | NA | 0 | 1 | 2 | 0 | 3 |
| 2a | *Hyalella clade 8* | NA | 6 | 0 | 0 | 0 | 6 |
| 2a | *Leptodora kindtii* | NA | 0 | 2 | 6 | 0 | 8 |
| 2a | *Limacina helicina* | NA | 0 | 0 | 0 | 1 | 1 |
| 2a | *Limnoperna fortunei* | NA | 32 | 1 | 11 | 1 | 45 |
| 2a | *Lophopanopeus bellus* | NA | 1 | 7 | 3 | 0 | 11 |
| 2a | *Nerita spp* | NA | 6 | 1 | 1 | 3 | 11 |
| 2a | *Polyphemus pediculus* | NA | 1 | 0 | 1 | 0 | 2 |
| 2a | *Tortanus discaudatus* | NA | 7 | 4 | 13 | 0 | 24 |
| 2b | *Chthamalus dalli* | 1 | 22117 | 87360 | 9683 | 19204 | 138364 |
| 2b | *Artemia spp* | 2 | 82148 | 285 | 136249 | 723 | 219405 |
| 2b | *Hyalella azteca clade 8* | 5 | 58960 | 0 | 0 | 0 | 58960 |
| 2b | *Leptodora kindtii* | 5 | 0 | 4270 | 31645 | 1130 | 37045 |
| 2b | *Balanus crenatus* | NA | 12 | 22 | 106 | 6 | 146 |
| 2b | *Balanus glandula* | NA | 2 | 0 | 0 | 0 | 2 |
| 2b | *Botryllus schlosseri* | NA | 0 | 3 | 0 | 0 | 3 |
| 2b | *Brachionus calyciflorus* | NA | 0 | 2 | 0 | 0 | 2 |
| 2b | *Centropages abdominalis* | NA | 1 | 0 | 0 | 0 | 1 |
| 2b | *Corbicula fluminea* | NA | 0 | 26 | 0 | 10 | 36 |
| 2b | *Crassostrea gigas* | NA | 0 | 0 | 1 | 0 | 1 |
| 2b | *Daphnia magna* | NA | 0 | 3 | 27 | 0 | 30 |
| 2b | *Daphnia pulex* | NA | 10 | 0 | 0 | 0 | 10 |
| 2b | *Eurytemora affinis* | NA | 54 | 8 | 55 | 1 | 118 |
| 2b | *Gammarus oceanicus* | NA | 0 | 11 | 1 | 0 | 12 |
| 2b | *Hyalella clade 1* | NA | 66 | 0 | 0 | 0 | 66 |
| 2b | *Leptodiaptomus minutus* | NA | 2 | 0 | 0 | 0 | 2 |
| 2b | *Limnoperna fortunei* | NA | 76 | 2 | 18 | 2 | 98 |
| 2b | *Lophopanopeus bellus* | NA | 0 | 3 | 8 | 0 | 11 |
| 2b | *Nerita spp* | NA | 5 | 0 | 1 | 3 | 9 |
| 2b | *Palaemonetes spp* | NA | 7 | 0 | 0 | 0 | 7 |
| 2b | *Tortanus discaudatus* | NA | 14 | 3 | 10 | 2 | 29 |
| 2c | *Nerita spp* | 1 | 31272 | 1370 | 9301 | 20703 | 62646 |
| 2c | *Carcinus maenas* | 2 | 168 | 2887 | 5 | 57 | 3117 |
| 2c | *Corbicula fluminea* | 5 | 9 | 118847 | 855 | 65196 | 184907 |
| 2c | *Daphnia pulex* | 10 | 65503 | 44 | 3617 | 1027 | 70191 |
| 2c | *Aglantha digitale* | NA | 5 | 0 | 0 | 0 | 5 |
| 2c | *Artemia spp* | NA | 14 | 0 | 11 | 0 | 25 |
| 2c | *Balanus crenatus* | NA | 12 | 37 | 81 | 12 | 142 |
| 2c | *Balanus glandula* | NA | 16 | 1 | 0 | 1 | 18 |
| 2c | *Botryllus schlosseri* | NA | 0 | 6 | 0 | 0 | 6 |
| 2c | *Brachionus calyciflorus* | NA | 0 | 73 | 0 | 0 | 73 |
| 2c | *Centropages abdominalis* | NA | 1 | 0 | 0 | 0 | 1 |
| 2c | *Cercopagis pengoi* | NA | 1 | 0 | 0 | 1 | 2 |
| 2c | *Chthamalus dalli* | NA | 3 | 28 | 20 | 1 | 52 |
| 2c | *Crangonidae* | NA | 1 | 0 | 0 | 0 | 1 |
| 2c | *Crassostrea gigas* | NA | 3 | 0 | 0 | 0 | 3 |
| 2c | *Daphnia magna* | NA | 12 | 0 | 16 | 0 | 28 |
| 2c | *Eurytemora affinis* | NA | 46 | 11 | 42 | 1 | 100 |
| 2c | *Hemigrapsus oregonensis* | NA | 0 | 6 | 14 | 1 | 21 |
| 2c | *Hyalella azteca clade 8* | NA | 4 | 0 | 0 | 0 | 4 |
| 2c | *Leptodiaptomus minutus* | NA | 3 | 0 | 0 | 0 | 3 |
| 2c | *Leptodora kindtii* | NA | 0 | 0 | 3 | 0 | 3 |
| 2c | *Limnoperna fortunei* | NA | 48 | 26 | 9 | 1 | 84 |
| 2c | *Lophopanopeus bellus* | NA | 11 | 67 | 9 | 0 | 87 |
| 2c | *Metacarcinus magister* | NA | 0 | 0 | 1 | 0 | 1 |
| 2c | *Mytilus edulis* | NA | 3 | 0 | 0 | 0 | 3 |
| 2c | *Palaemonetes spp* | NA | 29 | 0 | 0 | 0 | 29 |
| 2c | *Tortanus discaudatus* | NA | 21 | 1 | 18 | 1 | 41 |
| 2d | *Eurytemora affinis* | 23 | 75980 | 55610 | 220231 | 31413 | 383234 |
| 2d | *Artemia spp* | NA | 8 | 1 | 5 | 0 | 14 |
| 2d | *Balanus crenatus* | NA | 36 | 58 | 183 | 10 | 287 |
| 2d | *Balanus glandula* | NA | 2 | 0 | 0 | 0 | 2 |
| 2d | *Botryllus schlosseri* | NA | 0 | 4 | 0 | 0 | 4 |
| 2d | *Centropages abdominalis* | NA | 25 | 0 | 0 | 0 | 25 |
| 2d | *Chthamalus dalli* | NA | 4 | 11 | 1 | 2 | 18 |
| 2d | *Corbicula fluminea* | NA | 0 | 19 | 0 | 6 | 25 |
| 2d | *Crassostrea gigas* | NA | 3 | 0 | 0 | 0 | 3 |
| 2d | *Daphnia magna* | NA | 0 | 0 | 7 | 0 | 7 |
| 2d | *Daphnia pulex* | NA | 4 | 0 | 0 | 0 | 4 |
| 2d | *Diaphanosoma brachyurum* | NA | 167 | 0 | 0 | 0 | 167 |
| 2d | *Dreissena polymorpha or rostriformis bugensis* | NA | 1 | 0 | 0 | 0 | 1 |
| 2d | *Eucyclops speratus* | NA | 2 | 0 | 0 | 0 | 2 |
| 2d | *Hemigrapsus oregonensis* | NA | 0 | 1 | 3 | 0 | 4 |
| 2d | *Hyalella azteca clade 8* | NA | 4 | 0 | 0 | 0 | 4 |
| 2d | *Leptodiaptomus minutus* | NA | 8 | 2 | 0 | 0 | 10 |
| 2d | *Leptodora kindtii* | NA | 0 | 10 | 24 | 0 | 34 |
| 2d | *Limnoperna fortunei* | NA | 8 | 0 | 0 | 0 | 8 |
| 2d | *Lophopanopeus bellus* | NA | 0 | 8 | 6 | 0 | 14 |
| 2d | *Macrocyclops albidus* | NA | 1 | 0 | 0 | 0 | 1 |
| 2d | *Neotrypaea californiensis* | NA | 6 | 0 | 0 | 0 | 6 |
| 2d | *Nerita spp* | NA | 4 | 0 | 4 | 3 | 11 |
| 2d | *Palaemonetes spp* | NA | 24 | 0 | 0 | 0 | 24 |
| 2d | *Tisbe furcata* | NA | 1 | 0 | 0 | 0 | 1 |
| 2e | *Chthamalus dalli* | 1 | 1754 | 10880 | 89 | 5254 | 17977 |
| 2e | *Gammarus lawrencianus* | 1 | 0 | 99 | 110 | 54 | 263 |
| 2e | *Leptodiaptomus minutus* | 1 | 1509 | 496 | 237 | 8 | 2250 |
| 2e | *Nerita spp* | 1 | 1081 | 2 | 7 | 511 | 1601 |
| 2e | *Artemia spp* | 2 | 14504 | 15 | 1169 | 124 | 15812 |
| 2e | *Carcinus maenas* | 2 | 7 | 8 | 0 | 3 | 18 |
| 2e | *Corbicula fluminea* | 5 | 0 | 561 | 9 | 3341 | 3911 |
| 2e | *Hyalella clade 8* | 5 | 17518 | 0 | 0 | 0 | 17518 |
| 2e | *Leptodora kindtii* | 5 | 0 | 298 | 157 | 94 | 549 |
| 2e | *Palaemonetes spp* | 5 | 54207 | 0 | 0 | 0 | 54207 |
| 2e | *Balanus crenatus* | 10 | 34321 | 46910 | 196638 | 30798 | 308667 |
| 2e | *Daphnia pulex* | 10 | 3682 | 0 | 3 | 23 | 3708 |
| 2e | *Eurytemora affinis* | 23 | 30051 | 7415 | 74084 | 9078 | 120628 |
| 2e | *Balanus glandula* | NA | 2957 | 481 | 3 | 100 | 3541 |
| 2e | *Bythotrephes longimanus* | NA | 0 | 1 | 0 | 0 | 1 |
| 2e | *Centropages abdominalis* | NA | 3 | 0 | 0 | 0 | 3 |
| 2e | *Cercopagis pengoi* | NA | 3 | 0 | 0 | 0 | 3 |
| 2e | *Crangonidae* | NA | 11 | 0 | 0 | 0 | 11 |
| 2e | *Crassostrea gigas* | NA | 0 | 0 | 0 | 1 | 1 |
| 2e | *Daphnia magna* | NA | 2 | 0 | 11 | 0 | 13 |
| 2e | *Diaphanosoma brachyurum* | NA | 68 | 0 | 0 | 0 | 68 |
| 2e | *Hemigrapsus oregonensis* | NA | 0 | 3 | 8 | 2 | 13 |
| 2e | *Limnoperna fortunei* | NA | 7 | 1 | 0 | 0 | 8 |
| 2e | *Lophopanopeus bellus* | NA | 2 | 4 | 9 | 0 | 15 |
| 2e | *Macrocyclops albidus* | NA | 3 | 0 | 0 | 0 | 3 |
| 2e | *Mytilus edulis* | NA | 3 | 0 | 0 | 0 | 3 |
| 2e | *Tortanus discaudatus* | NA | 3 | 0 | 0 | 0 | 3 |
| 2g | *Asellus* | 1 | 1 | 0 | 0 | 0 | 1 |
| 2g | *Botryllus schlosseri* | 1 | 0 | 17393 | 2 | 20 | 17415 |
| 2g | *Caprella mutica* | 1 | 3 | 1 | 568 | 3 | 575 |
| 2g | *Hemimysis anomala* | 1 | 135 | 4 | 0 | 1 | 140 |
| 2g | *Limacina helicina* | 1 | 0 | 116 | 23 | 2637 | 2776 |
| 2g | *Macoma secta* | 1 | 1867 | 0 | 0 | 0 | 1867 |
| 2g | *Metacarcinus magister* | 1 | 0 | 598 | 466 | 1286 | 2350 |
| 2g | *Styela clava* | 1 | 284 | 18 | 0 | 1848 | 2150 |
| 2g | *Artemia spp* | 2 | 3019 | 15 | 1510 | 19 | 4563 |
| 2g | *Botrylloides violaceus* | 2 | 0 | 8 | 0 | 15 | 23 |
| 2g | *Corbicula fluminea* | 2 | 1 | 3339 | 6 | 2738 | 6084 |
| 2g | *Crassostrea gigas* | 2 | 13453 | 49 | 2693 | 1939 | 18134 |
| 2g | *Hemigrapsus oregonensis* | 2 | 0 | 4715 | 8063 | 595 | 13373 |
| 2g | *Lophopanopeus bellus* | 2 | 3862 | 27036 | 72664 | 2533 | 106095 |
| 2g | *Balanus crenatus* | 3 | 367 | 2491 | 21246 | 299 | 24403 |
| 2g | *Cercopagis pengoi* | 3 | 3756 | 23 | 146 | 1566 | 5491 |
| 2g | *Daphnia magna* | 3 | 2853 | 1204 | 132843 | 2264 | 139164 |
| 2g | *Limnoperna fortunei* | 3 | 53593 | 1451 | 1526 | 1272 | 57842 |
| 2g | *Microsetella norvegica* | 3 | 11 | 0 | 0 | 0 | 11 |
| 2g | *Acartia hudsonica* | 4 | 4 | 0 | 217 | 0 | 221 |
| 2g | *Tortanus discaudatus* | 4 | 1463 | 121 | 527 | 43 | 2154 |
| 2g | *Brachionus calyciflorus* | 5 | 15 | 8 | 0 | 5 | 28 |
| 2g | *Keratella quadrata* | 5 | 5 | 0 | 0 | 4 | 9 |
| 2g | *Balanus glandula* | NA | 1 | 0 | 0 | 0 | 1 |
| 2g | *Carcinus maenas* | NA | 0 | 2 | 0 | 0 | 2 |
| 2g | *Chthamalus dalli* | NA | 4 | 9 | 1 | 1 | 15 |
| 2g | *Daphnia pulex* | NA | 250 | 0 | 0 | 0 | 250 |
| 2g | *Dreissena polymorpha or rostriformis bugensis* | NA | 1 | 0 | 0 | 0 | 1 |
| 2g | *Eurytemora affinis* | NA | 10 | 4 | 19 | 2 | 35 |
| 2g | *Hyalella clade 8* | NA | 4 | 0 | 0 | 0 | 4 |
| 2g | *Leptodiaptomus minutus* | NA | 2 | 0 | 0 | 0 | 2 |
| 2g | *Leptodora kindtii* | NA | 0 | 4 | 7 | 0 | 11 |
| 2g | *Mytilus edulis* | NA | 0 | 0 | 15 | 2 | 17 |
| 2g | *Nerita spp* | NA | 4 | 0 | 1 | 4 | 9 |
| 2g | *Palaemonetes spp* | NA | 10 | 0 | 0 | 0 | 10 |
| 2g | *Pteropoda* | NA | 2 | 0 | 0 | 0 | 2 |
| 3a1 | *Limnoperna fortunei* | 1 | 105955 | 14326 | 149790 | 16161 | 286232 |
| 3a1 | *Balanus crenatus* | NA | 13 | 37 | 166 | 1 | 217 |
| 3a1 | *Balanus glandula* | NA | 1 | 2 | 0 | 0 | 3 |
| 3a1 | *Botryllus schlosseri* | NA | 0 | 2 | 0 | 0 | 2 |
| 3a1 | *Cercopagis pengoi* | NA | 3 | 0 | 0 | 1 | 4 |
| 3a1 | *Crassostrea gigas* | NA | 39 | 0 | 1 | 0 | 40 |
| 3a1 | *Daphnia magna* | NA | 0 | 2400 | 159360 | 632 | 162392 |
| 3a1 | *Hemigrapsus oregonensis* | NA | 0 | 1 | 0 | 0 | 1 |
| 3a1 | *Limacina helicina* | NA | 0 | 60 | 90 | 2582 | 2732 |
| 3a1 | *Lophopanopeus bellus* | NA | 2 | 4 | 0 | 0 | 6 |
| 3a1 | *Metacarcinus magister* | NA | 0 | 15 | 14 | 0 | 29 |
| 3a1 | *Mytilus edulis* | NA | 1 | 0 | 0 | 0 | 1 |
| 3a1 | *Pteropoda* | NA | 9 | 0 | 0 | 0 | 9 |
| 3a1 | *Styela clava* | NA | 0 | 0 | 0 | 5 | 5 |
| 3a1 | *Tortanus discaudatus* | NA | 1 | 0 | 9 | 0 | 10 |
| 3a2 | *Limnoperna fortunei* | 10 | 165347 | 14031 | 153880 | 10453 | 343711 |
| 3a2 | *Artemia spp* | NA | 0 | 0 | 1 | 0 | 1 |
| 3a2 | *Balanus crenatus* | NA | 7 | 21 | 120 | 0 | 148 |
| 3a2 | *Balanus glandula* | NA | 0 | 1 | 0 | 0 | 1 |
| 3a2 | *Bythotrephes longimanus* | NA | 0 | 1 | 0 | 0 | 1 |
| 3a2 | *Chthamalus dalli* | NA | 0 | 1 | 0 | 0 | 1 |
| 3a2 | *Daphnia magna* | NA | 0 | 0 | 14 | 0 | 14 |
| 3a2 | *Eurytemora affinis* | NA | 0 | 0 | 2 | 0 | 2 |
| 3a2 | *Leptodora kindtii* | NA | 0 | 0 | 1 | 0 | 1 |
| 3a2 | *Limacina helicina* | NA | 0 | 0 | 0 | 2 | 2 |
| 3a2 | *Mytilus edulis* | NA | 1 | 0 | 0 | 0 | 1 |
| 3a2 | *Tortanus discaudatus* | NA | 2 | 1 | 5 | 0 | 8 |
| 3a3 | *Limnoperna fortunei* | 30 | 207171 | 23678 | 237670 | 18006 | 486525 |
| 3a3 | *Balanus crenatus* | NA | 11 | 36 | 126 | 1 | 174 |
| 3a3 | *Balanus glandula* | NA | 2 | 1 | 0 | 0 | 3 |
| 3a3 | *Chthamalus dalli* | NA | 0 | 0 | 0 | 1 | 1 |
| 3a3 | *Corbicula fluminea* | NA | 0 | 2 | 0 | 1 | 3 |
| 3a3 | *Daphnia magna* | NA | 0 | 0 | 13 | 0 | 13 |
| 3a3 | *Hemigrapsus oregonensis* | NA | 0 | 0 | 4 | 0 | 4 |
| 3a3 | *Limacina helicina* | NA | 0 | 1 | 0 | 0 | 1 |
| 3a3 | *Nerita spp* | NA | 3 | 0 | 0 | 0 | 3 |
| 3a3 | *Tortanus discaudatus* | NA | 2 | 1 | 14 | 1 | 18 |
| 3b1 | *Balanus crenatus* | 1 | 49784 | 87106 | 228805 | 4644 | 370339 |
| 3b1 | *Balanus glandula* | NA | 3 | 1 | 0 | 0 | 4 |
| 3b1 | *Chthamalus dalli* | NA | 3 | 2 | 0 | 0 | 5 |
| 3b1 | *Daphnia magna* | NA | 0 | 0 | 18 | 0 | 18 |
| 3b1 | *Eurytemora affinis* | NA | 0 | 0 | 5 | 0 | 5 |
| 3b1 | *Leptodora kindtii* | NA | 0 | 6 | 14 | 0 | 20 |
| 3b1 | *Limnoperna fortunei* | NA | 53 | 7 | 68 | 4 | 132 |
| 3b2 | *Balanus crenatus* | 10 | 42001 | 73379 | 215991 | 1923 | 333294 |
| 3b2 | *Artemia spp* | NA | 0 | 0 | 1 | 0 | 1 |
| 3b2 | *Balanus glandula* | NA | 3053 | 7250 | 664 | 9 | 10976 |
| 3b2 | *Centropages abdominalis* | NA | 1 | 0 | 0 | 0 | 1 |
| 3b2 | *Chthamalus dalli* | NA | 20 | 4 | 1 | 0 | 25 |
| 3b2 | *Daphnia magna* | NA | 0 | 0 | 18 | 0 | 18 |
| 3b2 | *Eurytemora affinis* | NA | 1 | 2 | 1 | 0 | 4 |
| 3b2 | *Leptodora kindtii* | NA | 0 | 7 | 7 | 0 | 14 |
| 3b2 | *Limnoperna fortunei* | NA | 60 | 7 | 71 | 1 | 139 |
| 3b2 | *Mytilus edulis* | NA | 1 | 0 | 0 | 0 | 1 |
| 3b2 | *Palaemonetes spp* | NA | 1 | 0 | 0 | 0 | 1 |
| 3b3 | *Balanus crenatus* | 17 | 39405 | 7412 | 198797 | 2 | 245616 |
| 3b3 | *Acartia longiremis* | NA | 1 | 0 | 0 | 0 | 1 |
| 3b3 | *Balanus glandula* | NA | 3 | 0 | 0 | 0 | 3 |
| 3b3 | *Chthamalus dalli* | NA | 1 | 0 | 0 | 0 | 1 |
| 3b3 | *Crassostrea gigas* | NA | 1 | 0 | 0 | 0 | 1 |
| 3b3 | *Daphnia magna* | NA | 0 | 0 | 18 | 0 | 18 |
| 3b3 | *Hemigrapsus oregonensis* | NA | 0 | 0 | 1 | 0 | 1 |
| 3b3 | *Leptodora kindtii* | NA | 0 | 3 | 2 | 0 | 5 |
| 3b3 | *Limacina helicina* | NA | 0 | 0 | 0 | 1 | 1 |
| 3b3 | *Limnoperna fortunei* | NA | 84 | 6 | 62 | 2 | 154 |
| 3c1 | *Tortanus discaudatus* | 1 | 71308 | 36404 | 318338 | 19419 | 445469 |
| 3c1 | *Acartia hudsonica* | NA | 323 | 0 | 4 | 0 | 327 |
| 3c1 | *Balanus crenatus* | NA | 1 | 1 | 37 | 1 | 40 |
| 3c1 | *Balanus glandula* | NA | 0 | 5 | 0 | 0 | 5 |
| 3c1 | *Botryllus schlosseri* | NA | 0 | 4 | 0 | 0 | 4 |
| 3c1 | *Brachionus calyciflorus* | NA | 0 | 8 | 0 | 0 | 8 |
| 3c1 | *Ceriodaphnia lacustris* | NA | 0 | 0 | 1 | 0 | 1 |
| 3c1 | *Chthamalus dalli* | NA | 0 | 5 | 0 | 0 | 5 |
| 3c1 | *Crassostrea gigas* | NA | 2 | 0 | 0 | 0 | 2 |
| 3c1 | *Daphnia magna* | NA | 0 | 633 | 6720 | 69 | 7422 |
| 3c1 | *Eurytemora affinis* | NA | 8 | 0 | 0 | 0 | 8 |
| 3c1 | *Hemigrapsus oregonensis* | NA | 0 | 0 | 0 | 3 | 3 |
| 3c1 | *Holopedium gibberum* | NA | 0 | 0 | 1 | 0 | 1 |
| 3c1 | *Leptodora kindtii* | NA | 0 | 93 | 144 | 2 | 239 |
| 3c1 | *Limacina helicina* | NA | 0 | 0 | 0 | 5 | 5 |
| 3c1 | *Limnoperna fortunei* | NA | 8 | 2 | 23 | 0 | 33 |
| 3c1 | *Lophopanopeus bellus* | NA | 1 | 0 | 0 | 0 | 1 |
| 3c1 | *Palaemonetes spp* | NA | 1 | 0 | 0 | 0 | 1 |
| 3c1 | *Polyphemus pediculus* | NA | 0 | 0 | 1 | 0 | 1 |
| 3c2 | *Tortanus discaudatus* | 8 | 86973 | 35889 | 184408 | 30596 | 337866 |
| 3c2 | *Acartia hudsonica* | NA | 337 | 0 | 971 | 0 | 1308 |
| 3c2 | *Artemia spp* | NA | 2 | 0 | 14 | 0 | 16 |
| 3c2 | *Daphnia magna* | NA | 0 | 20414 | 151378 | 2878 | 174670 |
| 3c2 | *Eurytemora affinis* | NA | 4 | 0 | 10 | 0 | 14 |
| 3c2 | *Gammarus oceanicus* | NA | 0 | 0 | 4 | 0 | 4 |
| 3c2 | *Hemigrapsus oregonensis* | NA | 0 | 3 | 0 | 0 | 3 |
| 3c2 | *Hyalella azteca clade 8* | NA | 1 | 0 | 0 | 0 | 1 |
| 3c2 | *Leptodora kindtii* | NA | 0 | 82 | 128 | 5 | 215 |
| 3c2 | *Limnoperna fortunei* | NA | 15 | 2 | 22 | 0 | 39 |
| 3c3 | *Tortanus discaudatus* | 15 | 75067 | 60966 | 345532 | 24873 | 506438 |
| 3c3 | *Acartia hudsonica* | NA | 332 | 0 | 373 | 0 | 705 |
| 3c3 | *Corbicula fluminea* | NA | 0 | 1 | 0 | 0 | 1 |
| 3c3 | *Crangonidae* | NA | 4 | 0 | 0 | 0 | 4 |
| 3c3 | *Daphnia magna* | NA | 0 | 117 | 2276 | 24 | 2417 |
| 3c3 | *Eurytemora affinis* | NA | 70 | 0 | 0 | 0 | 70 |
| 3c3 | *Gammarus oceanicus* | NA | 0 | 1 | 0 | 0 | 1 |
| 3c3 | *Hemigrapsus oregonensis* | NA | 0 | 0 | 21 | 0 | 21 |
| 3c3 | *Leptodora kindtii* | NA | 0 | 133 | 141 | 6 | 280 |
| 3c3 | *Limacina helicina* | NA | 0 | 0 | 0 | 1 | 1 |
| 3c3 | *Limnoperna fortunei* | NA | 9 | 2 | 28 | 0 | 39 |
| 3c3 | *Mytilus edulis* | NA | 1 | 0 | 0 | 0 | 1 |
| 3c3 | *Styela clava* | NA | 0 | 0 | 0 | 22 | 22 |
| 3d1 | *Leptodora kindtii* | 1 | 10 | 104481 | 294868 | 0 | 399359 |
| 3d1 | *Balanus crenatus* | NA | 3 | 12 | 40 | 0 | 55 |
| 3d1 | *Centropages abdominalis* | NA | 2 | 0 | 0 | 0 | 2 |
| 3d1 | *Corbicula fluminea* | NA | 0 | 0 | 0 | 3 | 3 |
| 3d1 | *Daphnia magna* | NA | 0 | 0 | 13 | 0 | 13 |
| 3d1 | *Eurytemora affinis* | NA | 10 | 0 | 27 | 0 | 37 |
| 3d1 | *Leptodiaptomus minutus* | NA | 1 | 1 | 0 | 0 | 2 |
| 3d1 | *Macrocyclops albidus* | NA | 1 | 0 | 0 | 0 | 1 |
| 3d1 | *Tortanus discaudatus* | NA | 25 | 27 | 148 | 0 | 200 |
| 3d2 | *Leptodora kindtii* | 10 | 8 | 167544 | 301656 | 16348 | 485556 |
| 3d2 | *Artemia spp* | NA | 0 | 0 | 1 | 0 | 1 |
| 3d2 | *Balanus crenatus* | NA | 4 | 5 | 51 | 1 | 61 |
| 3d2 | *Bythotrephes longimanus* | NA | 0 | 0 | 12 | 0 | 12 |
| 3d2 | *Chthamalus dalli* | NA | 0 | 1 | 0 | 0 | 1 |
| 3d2 | *Crangonidae* | NA | 2 | 0 | 0 | 0 | 2 |
| 3d2 | *Daphnia magna* | NA | 0 | 0 | 16 | 0 | 16 |
| 3d2 | *Eurytemora affinis* | NA | 7 | 0 | 14 | 0 | 21 |
| 3d2 | *Gammarus oceanicus* | NA | 0 | 0 | 1 | 0 | 1 |
| 3d2 | *Hemigrapsus oregonensis* | NA | 0 | 0 | 11 | 1 | 12 |
| 3d2 | *Leptodiaptomus minutus* | NA | 227 | 0 | 0 | 0 | 227 |
| 3d2 | *Limnoperna fortunei* | NA | 2 | 0 | 0 | 0 | 2 |
| 3d2 | *Lophopanopeus bellus* | NA | 0 | 0 | 1 | 0 | 1 |
| 3d2 | *Tortanus discaudatus* | NA | 16 | 14 | 154 | 6 | 190 |
| 3d3 | *Leptodora kindtii* | 28 | 8 | 139830 | 164686 | 18336 | 322860 |
| 3d3 | *Artemia spp* | NA | 1 | 0 | 0 | 0 | 1 |
| 3d3 | *Balanus crenatus* | NA | 2 | 0 | 36 | 0 | 38 |
| 3d3 | *Cercopagis pengoi* | NA | 0 | 0 | 0 | 1 | 1 |
| 3d3 | *Crassostrea gigas* | NA | 1 | 0 | 0 | 0 | 1 |
| 3d3 | *Daphnia magna* | NA | 0 | 1 | 30 | 0 | 31 |
| 3d3 | *Daphnia pulex* | NA | 1 | 0 | 0 | 0 | 1 |
| 3d3 | *Eurytemora affinis* | NA | 1 | 1 | 0 | 0 | 2 |
| 3d3 | *Hemigrapsus oregonensis* | NA | 0 | 1 | 4 | 1 | 6 |
| 3d3 | *Holopedium gibberum* | NA | 0 | 0 | 2 | 0 | 2 |
| 3d3 | *Leptodiaptomus minutus* | NA | 2 | 0 | 0 | 0 | 2 |
| 3d3 | *Limnoperna fortunei* | NA | 2 | 0 | 0 | 0 | 2 |
| 3d3 | *Lophopanopeus bellus* | NA | 0 | 2 | 4 | 0 | 6 |
| 3d3 | *Macoma secta* | NA | 1 | 0 | 0 | 0 | 1 |
| 3d3 | *Tortanus discaudatus* | NA | 14 | 23 | 121 | 6 | 164 |

Table S8: Number of reads assigned to the expected species in the mock communities, and to the other species in the local database as contamination.

| Library | Species Expected | Species Detected | 18S | | | FC | | | Leray | | | Folmer | | |
| --- | --- | --- | --- | --- | --- | --- | --- | --- | --- | --- | --- | --- | --- | --- |
|  |  |  | Assigned | Expected | Contamination | Assigned | Expected | Contamination | Assigned | Expected | Contamination | Assigned | Expected | Contamination |
| 1a | 10 | 6 | 39923 | 39753 | 170 | 4062 | 3985 | 77 | 151626 | 151405 | 221 | 11945 | 11926 | 19 |
| 1b | 16 | 11 | 91986 | 87483 | 4503 | 55333 | 55271 | 62 | 238036 | 237235 | 801 | 22046 | 22036 | 10 |
| 1c | 14 | 10 | 167000 | 156834 | 10166 | 73084 | 46599 | 26485 | 231414 | 13518 | 217896 | 32195 | 29004 | 3191 |
| 1d | 16 | 12 | 123506 | 117375 | 6131 | 15556 | 13049 | 2507 | 71483 | 13731 | 57752 | 5380 | 349 | 5031 |
| 1e | 56 | 40 | 153955 | 148426 | 5529 | 61232 | 41528 | 19704 | 187120 | 78683 | 108437 | 41520 | 38456 | 3064 |
| 1g | 27 | 25 | 103269 | 103044 | 225 | 49898 | 49866 | 32 | 212793 | 212745 | 48 | 14444 | 14436 | 8 |
| 2a | 4 | 4 | 216345 | 208734 | 7611 | 81245 | 79938 | 1307 | 306006 | 305857 | 149 | 34287 | 34103 | 184 |
| 2b | 5 | 4 | 163474 | 163225 | 249 | 91998 | 91915 | 83 | 177804 | 177577 | 227 | 21081 | 21057 | 24 |
| 2c | 4 | 4 | 97185 | 96952 | 233 | 123404 | 123148 | 256 | 14002 | 13778 | 224 | 87002 | 86983 | 19 |
| 2d | 1 | 1 | 76288 | 75980 | 308 | 55724 | 55610 | 114 | 220464 | 220231 | 233 | 31434 | 31413 | 21 |
| 2e | 14 | 13 | 161696 | 158634 | 3062 | 67174 | 66684 | 490 | 272534 | 272503 | 31 | 49391 | 49288 | 103 |
| 2g | 26 | 23 | 84980 | 84692 | 288 | 58609 | 58590 | 19 | 242543 | 242500 | 43 | 19096 | 19087 | 9 |
| 3a1 | 1 | 1 | 106024 | 105955 | 69 | 16847 | 14326 | 2521 | 309430 | 149790 | 159640 | 19382 | 16161 | 3221 |
| 3a2 | 1 | 1 | 165357 | 165347 | 10 | 14056 | 14031 | 25 | 154023 | 153880 | 143 | 10455 | 10453 | 2 |
| 3a3 | 1 | 1 | 207189 | 207171 | 18 | 23719 | 23678 | 41 | 237827 | 237670 | 157 | 18010 | 18006 | 4 |
| 3b1 | 1 | 1 | 49843 | 49784 | 59 | 87122 | 87106 | 16 | 228910 | 228805 | 105 | 4648 | 4644 | 4 |
| 3b2 | 1 | 1 | 45138 | 42001 | 3137 | 80649 | 73379 | 7270 | 216754 | 215991 | 763 | 1933 | 1923 | 10 |
| 3b3 | 1 | 1 | 39495 | 39405 | 90 | 7421 | 7412 | 9 | 198880 | 198797 | 83 | 5 | 2 | 3 |
| 3c1 | 1 | 1 | 71652 | 71308 | 344 | 37155 | 36404 | 751 | 325269 | 318338 | 6931 | 19499 | 19419 | 80 |
| 3c2 | 1 | 1 | 87332 | 86973 | 359 | 56390 | 35889 | 20501 | 336935 | 184408 | 152527 | 33479 | 30596 | 2883 |
| 3c3 | 1 | 1 | 75483 | 75067 | 416 | 61220 | 60966 | 254 | 348371 | 345532 | 2839 | 24926 | 24873 | 53 |
| 3d1 | 1 | 1 | 52 | 10 | 42 | 104521 | 104481 | 40 | 295096 | 294868 | 228 | 3 | 0 | 3 |
| 3d2 | 1 | 1 | 266 | 8 | 258 | 167564 | 167544 | 20 | 301917 | 301656 | 261 | 16356 | 16348 | 8 |
| 3d3 | 1 | 1 | 33 | 8 | 25 | 139858 | 139830 | 28 | 164883 | 164686 | 197 | 18344 | 18336 | 8 |

Table S9: Mock communities assemblage

| Community Type | Library | # Species | # Individuals/Species | Total # Individuals |
| --- | --- | --- | --- | --- |
| SIS | 1a | 10 | 1 | 10 |
| SIS | 1b | 16 | 1 | 16 |
| SIS | 1c | 14 | 1 | 14 |
| SIS | 1d | 16 | 1 | 16 |
| SIS | 1e | 56 | 1 | 56 |
| SIS | 1g | 27 | 1 | 27 |
| MIS | 2a | 4 | 1-10 | 17 |
| MIS | 2b | 5 | 1-5 | 18 |
| MIS | 2c | 4 | 1-10 | 18 |
| MIS | 2d | 1 | 23 | 23 |
| MIS | 2e | 14 | 1-23 | 76 |
| MIS | 2g | 26 | 1-5 | 62 |
| PSS | 3a1 | 1 | 1 | 1 |
| PSS | 3a2 | 1 | 10 | 10 |
| PSS | 3a3 | 1 | 30 | 30 |
| PSS | 3b1 | 1 | 1 | 1 |
| PSS | 3b2 | 1 | 10 | 10 |
| PSS | 3b3 | 1 | 17 | 17 |
| PSS | 3c1 | 1 | 1 | 1 |
| PSS | 3c2 | 1 | 8 | 8 |
| PSS | 3c3 | 1 | 15 | 15 |
| PSS | 3d1 | 1 | 1 | 1 |
| PSS | 3d2 | 1 | 10 | 10 |
| PSS | 3d3 | 1 | 28 | 28 |

Fig. S1: Comparison of species detection rates between PCR & gel electrophoresis vs. NGS metabarcoding on the 49 species used in both the primer testing and mock communities. ‘PCR only’ (left side in creamy) refers to the species detected only by PCR and gel electrophoresis (ie. when single species were amplified but not from metabarcoding). ‘NGS only’ (right side in blue) refers to the species detected only by next-generation sequencing mock communities in the metabarcoding approach. “All” and “COI” boxes refer to all 3 COI primer pairs, “FC”, “Leray”, and “Folmer” boxes refer to 3 different COI primer pairs.


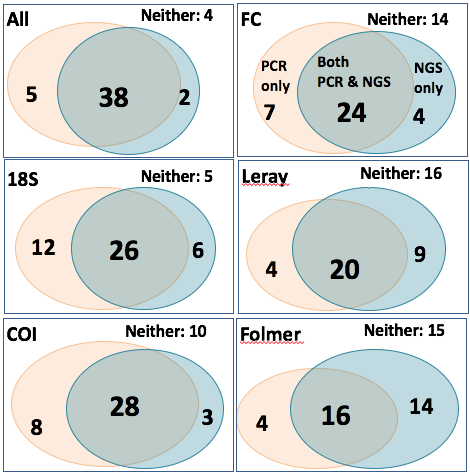


Fig. S2 The major steps involved during taxonomic assignment.


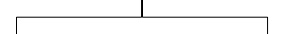


Appendix S1: Scripting for the Bioinformatic Analysis

# Step1_Trim Raw Reads using FastX toolkit and Check with FastQC reports

# Step2_Blast Trimmed Reads against the Local Reference Database

# Step3_Filter Blast Hits for each read

# Step4_Combine R1 and R2 Blast results using R

# Step5_Summarize Blast results for each library

# Step1_Trim Raw Reads using FastX toolkit and Check with FastQC reports

#Bash

cd {files_location}

R1=R1.fastq.gz

R2=R2.fastq.gz

zcat $R1 | fastq_quality_trimmer -t 20 -l 150 -Q 33 > R1_trimmed.fastq

zcat $R2 | fastq_quality_trimmer -t 20 -l 150 -Q 33 > R2_trimmed.fastq

fastqc R1_trimmed.fastq

fastqc R2_trimmed.fastq

sed -n '1~4s/^@/>/p;2~4p' R1_trimmed.fastq > R1_trimmed.fasta

sed -n '1~4s/^@/>/p;2~4p' R2_trimmed.fastq > R2_trimmed.fasta

# Step2_Blast Trimmed Reads against the Local Reference Database

#Bash

DB=Local_Reference_Database.fasta

blast -task blastn -db $DB -query R1_trimmed.fasta -out R1-trimmed.blast.out -max_target_seqs 5 -num_threads 12 -evalue 0.001 -outfmt "6 sstart send qstart qend qseqid sseqid pident length score evalue"

blast -task blastn -db $DB -query R2_trimmed.fasta -out R2-trimmed.blast.out -max_target_seqs 5 -num_threads 12 -evalue 0.001 -outfmt "6 sstart send qstart qend qseqid sseqid pident length score evalue"

# Step3_Filter Blast Hits for each read

#Bash

for file in R1-trimmed.blast.out R2-trimmed.blast.out;do

threshLen=150

cat $file | awk -v len=$threshLen '$8>len {print}' | awk '{OFS="\t";if($5==prev){if(id==$7 && len==$8){hit=hit";"$6;$6=hit;j=$0}}else{if(NR>1){print j}else{print "Start","End","Qstart","Qend","Reads","Taxa","Pident","Length","Score","Evalue"};j=$0;hit=$6;id=$7;len=$8}prev=$5}END{print j}' > $file.tophits

done

# Step4_Combine R1 and R2 Blast results using R

#R

library(stringr)

library(plyr)

file1<-“R1-trimmed.blast.out.tophits”

file2<-“R2-trimmed.blast.out.tophits”

name<-“R1R2”

columns<-5

data1<-read.delim(file1,header=T,sep="\t",row.names=columns)

data2<-read.delim(file2,header=T,sep="\t",row.names=columns)

### Merge data by rows

hits<-merge(data1,data2,by="row.names",all=T)

write.table(file=paste(name,"info",sep="."),hits,quote=F,sep="\t",row.names=F,col.names=T)

# Step5_Summarize Blast results for each library

#BASH

echo "File Reads" > RawReadCount.out

for i in *.fastq.gz;do ###loop through raw read files

echo -n "$i " >> RawReadCount.out

zcat $i | echo $((`wc -l`/4)) >> RawReadCount.out

done

#BASH

threshold=95

Summary="Summary.$threshold.Reads.out"

Summary2="Summary.$threshold.Species.out"

echo "Library RawReads ReadsWithBothBLASTHit ReadsWithBothBLASTHitProp ReadsWithBothBLASTHitSameSpecies ReadsWithBothBLASTHitSameSpeciesProp ReadsWithBLASTHitRead1 ReadsWithAnyBLASTHitRead1Prop ReadsWithBLASTHitRead2 ReadsWithAnyBLASTHitRead2Prop 18SBoth FCBoth LerayBoth FolmerBoth 18SBothProp FCBothProp LerayBothProp FolmerBothProp 18SRead1 FCRead1 LerayRead1 FolmerRead1 18SRead1Prop FCRead1Prop LerayRead1Prop FolmerRead1Prop 18SRead2 FCRead2 LerayRead2 FolmerRead2 18SRead2Prop FCRead2Prop LerayRead2Prop FolmerRead2Prop TotalNum_Species 18S_Species FC_Species Leray_Species Folmer_Species TotalNum_Species_Read1 18S_Species_Read1 FC_Species_Read1 Leray_Species_Read1 Folmer_Species_Read1 TotalNum_Species_Read2 18S_Species_Read2 FC_Species_Read2 Leray_Species_Read2 Folmer_Species_Read2" > $Summary

echo "Library Species 18S FC Leray Folmer 18SProp FCProp LerayProp FolmerProp" > $Summary2

done

###now loop through every library and summarize species overall and then by fragment

thresh=$threshold

for file in *info;do ###loop through file

library=${file%.*}

Fsummary="$file.$threshold.summary"

Raw=`grep $library /RawReadCount.out | awk '{sum+=$2}END{print sum}'`

All=`sed '1,1d' $file | awk -v T=$thresh '{if($7>T || $16>T){print $0}}' | wc -l`

NA1only=`cat $file | awk '{if($3=="NA" && $12!="NA"){print $0}}' | awk -v T=$thresh '{if($16>T){print $0}}' |wc -l`

NA2only=`cat $file | awk '{if($3!="NA" && $12=="NA"){print $0}}' | awk -v T=$thresh '{if($7>T){print $0}}' |wc -l`

r1=`sed '1,1d' $file | awk -v T=$thresh '{if($6!="NA" && $7>T){print $0}}' | wc -l`

r2=`sed '1,1d' $file | awk -v T=$thresh '{if($15!="NA" && $16>T){print $0}}' | wc -l`

Opposite=`cat $file | sed '1,1d' | grep -v " NA " | awk '{if($6!=$15){print $0}}' | awk -v T=$thresh '{if($7>T && $16>T){print $0}}' | wc -l`

Both=`grep -v " NA " $file | awk -v T=$thresh '{if($7>T && $16>T){print $0}}' | wc -l`

BothSame=`grep -v " NA " $file | awk '{if($6==$15){print $0}}' | awk -v T=$thresh '{if($7>T && $16>T){print $0}}' | wc -l`

##Fragments (18S, FC, Leray, and Folmer), based on where reads map on the reference

eS=`grep 18S $file | awk -v T=$thresh '{if($6==$15 && $7>T && $16>T){print $0}}' | wc -l`

FC=`grep COI $file | awk -v T=$thresh '{if($6==$15 && $7>T && $16>T){print $0}}' | awk '{if(($2<400 && $3<400 && $11<400 && $12<400)){print $0}}' | wc -l`

Leray=`grep COI $file | awk -v T=$thresh '{if($6==$15 && $7>T && $16>T){print $0}}' | awk '{if(($2>300 && $3>300 && $11>300 && $12>300)){print $0}}' | wc -l`

Folmer=`grep COI $file | awk -v T=$thresh '{if($6==$15 && $7>T && $16>T){print $0}}' | awk '{if(($2<300 || $3<300 || $11<300 || $12<300) && ($2>400 || $4>500 || $11>400 || $12>400)){print $0}}' | wc -l`

eSr1=`grep 18S $file | awk -v T=$thresh '{if($6!="NA" && $7>T){print $6}}' | wc -l`

FCr1=`grep COI $file | awk -v T=$thresh '{if($6!="NA" && $7>T){print $0}}' | awk '{if(($2<400 && $3<400)){print $6}}' | wc -l`

Lerayr1=`grep COI $file | awk -v T=$thresh '{if($6!="NA" && $7>T){print $0}}' | awk '{if(($2>300 && $3>300)){print $6}}' |wc -l`

Folmerr1=`grep COI $file | awk -v T=$thresh '{if($6!="NA" && $7>T){print $0}}' | awk '{if(($2<300 || $3<300) && ($2>400 || $3>400)){print $6}}' |wc -l`

eSr2=`grep 18S $file | awk -v T=$thresh '{if($15!="NA" && $16>T){print $15}}' | wc -l`

FCr2=`grep COI $file | awk -v T=$thresh '{if($15!="NA" && $16>T){print $0}}' | awk '{if(($11<400 && $12<400)){print $15}}' | wc -l`

Lerayr2=`grep COI $file | awk -v T=$thresh '{if($15!="NA" && $16>T){print $0}}' | awk '{if(($11>300 && $12>300)){print $15}}' | wc -l`

Folmerr2=`grep COI $file | awk -v T=$thresh '{if($15!="NA" && $16>T){print $0}}' | awk '{if(($11<300 || $12<300) && ($11>400 || $12>400)){print $15}}' | wc -l`

##Species summaries, overall

spAll=`sed 1,1d $file | awk '{if($6==$15){print $0}}' | awk -v T=$thresh '{if($7>T && $16>T){print $6}}' | sort | uniq | wc -l`

spAllr1=`sed 1,1d $file | awk -v T=$thresh '{if($6!="NA" && $7>T){print $6}}' | sort | uniq | wc -l`

spAllr2=`sed 1,1d $file | awk -v T=$thresh '{if($15!="NA" && $16>T){print $15}}' | sort | uniq | wc -l`

##Species summaries per fragment

speS=`grep 18S $file | awk -v T=$thresh '{if($6==$15 && $7>T && $16>T){print $0}}' | cut -f 6 | sort | uniq | wc -l`

spFC=`grep COI $file | awk -v T=$thresh '{if($6==$15 && $7>T && $16>T){print $0}}' | awk '{if(($2<400 && $3<400 && $11<400 && $12<400)){print $0}}' | cut -f 6 | sort | uniq | wc -l`

spLeray=`grep COI $file | awk -v T=$thresh '{if($6==$15 && $7>T && $16>T){print $0}}' | awk '{if(($2>300 && $3>300 && $11>300 && $12>300)){print $0}}' | cut -f 6 | sort | uniq | wc -l`

spFolmer=`grep COI $file | awk -v T=$thresh '{if($6==$15 && $7>T && $16>T){print $0}}' | awk '{if(($2<300 || $3<300 || $11<300 || $12<300) && ($2>400 || $3>400 || $11>400 || $12>400)){print $0}}' | cut -f 6 | sort | uniq | wc -l`

speSr1=`grep 18S $file | awk -v T=$thresh '{if($6!="NA" && $7>T){print $0}}' | cut -f 6 | sort | uniq | wc -l`

spFCr1=`grep COI $file | awk -v T=$thresh '{if($6!="NA" && $7>T){print $0}}' | awk '{if(($2<400 && $3<400)){print $6}}' | sort | uniq | wc -l`

spLerayr1=`grep COI $file | awk -v T=$thresh '{if($6!="NA" && $7>T){print $0}}' | awk '{if(($2>300 && $3>300)){print $6}}' | sort | uniq | wc -l`

spFolmerr1=`grep COI $file | awk -v T=$thresh '{if($6!="NA" && $7>T){print $0}}' | awk '{if(($2<300 || $3<300) && ($2>400 || $3>400)){print $6}}' | sort | uniq | wc -l`

speSr2=`grep 18S $file | awk -v T=$thresh '{if($15!="NA" && $16>T){print $0}}' | cut -f 15 | sort | uniq | wc -l`

spFCr2=`grep COI $file | awk -v T=$thresh '{if($15!="NA" && $16>T){print $0}}' | awk '{if(($11<400 && $12<400)){print $15}}' | sort | uniq | wc -l`

spLerayr2=`grep COI $file | awk -v T=$thresh '{if($15!="NA" && $16>T){print $0}}' | awk '{if(($11>300 && $12>300)){print $15}}' |sort | uniq | wc -l`

spFolmerr2=`grep COI $file | awk -v T=$thresh '{if($15!="NA" && $16>T){print $0}}' | awk '{if(($11<300 || $12<300) && ($11>400 || $12>400)){print $15}}' | sort | uniq | wc -l`

###Proportions

AllP=`printf %.2f $(echo "$All / $Raw" | bc -l)`

NA1onlyP=`printf %.2f $(echo "$NA1only / $All" | bc -l)`

NA2onlyP=`printf %.2f $(echo "$NA2only / $All" | bc -l)`

r1P=`printf %.2f $(echo "$r1 / $All" | bc -l)`

r2P=`printf %.2f $(echo "$r2 / $All" | bc -l)`

OppositeP=`printf %.2f $(echo "$Opposite / $All" | bc -l)`

BothP=`printf %.2f $(echo "$Both / $All" | bc -l)`

BothSameP=`printf %.2f $(echo "$BothSame / $All" | bc -l)`

eSP=`printf %.2f $(echo "$eS / $BothSame" | bc -l)`

FCP=`printf %.2f $(echo "$FC / $BothSame" | bc -l)`

LerayP=`printf %.2f $(echo "$Leray / $BothSame" | bc -l)`

FolmerP=`printf %.2f $(echo "$Folmer / $BothSame" | bc -l)`

eSr1P=`printf %.2f $(echo "$eSr1 / $r1" | bc -l)`

FCr1P=`printf %.2f $(echo "$FCr1 / $r1" | bc -l)`

Lerayr1P=`printf %.2f $(echo "$Lerayr1 / $r1" | bc -l)`

Folmerr1P=`printf %.2f $(echo "$Folmerr1 / $r1" | bc -l)`

eSr2P=`printf %.2f $(echo "$eSr2 / $r2" | bc -l)`

FCr2P=`printf %.2f $(echo "$FCr2 / $r2" | bc -l)`

Lerayr2P=`printf %.2f $(echo "$Lerayr2 / $r2" | bc -l)`

Folmerr2P=`printf %.2f $(echo "$Folmerr2 / $r2" | bc -l)`

##Print

echo "$library $Raw $Both $BothP $BothSame $BothSameP $r1 $r1P $r2 $r2P $eS $FC $Leray $Folmer $eSP $FCP $LerayP $FolmerP $eSr1 $FCr1 $Lerayr1 $Folmerr1 $eSr1P $FCr1P $Lerayr1P $Folmerr1P $eSr2 $FCr2 $Lerayr2 $Folmerr2 $eSr2P $FCr2P $Lerayr2P $Folmerr2P $spAll $speS $spFC $spLeray $spFolmer $spAllr1 $speSr1 $spFCr1 $spLerayr1 $spFolmerr1 $spAllr2 $speSr2 $spFCr2 $spLerayr2 $spFolmerr2" >> $Summary

##Print out all species, and number of reads per species per fragment

Species=`sed '1,1d' $file | awk '{if($6==$15){print $0}}' | awk -v T=$thresh '{if($7>T && $16>T){print $0}}' | cut -f 6 | sort | uniq`

previous=""

for sp in $Species;do ###loop through species

if [[ $sp != $previous ]];then

if [[ $sp =~ ";" ]];then

eS=`cat $file | grep "$sp" | grep -v COI | awk '{if($6==$15){print $0}}' | awk -v T=$thresh '{if($7>T && $16>T){print $0}}' | wc -l`

FC=`cat $file | grep "$sp" | grep -v 18S | awk '{if($6==$15){print $0}}' | awk '{if(($2<400 && $3<400 && $11<400 && $12<400)){print $0}}' | awk -v T=$thresh '{if($7>T && $16>T){print $0}}' | wc -l`

Leray=`cat $file | grep "$sp" | grep -v 18S | awk '{if($6==$15){print $0}}' | awk '{if(($2>300 && $3>300 && $11>300 && $12>300)){print $0}}' | awk -v T=$thresh '{if($7>T && $16>T){print $0}}' | wc -l`

Folmer=`cat $file | grep "$sp" | grep -v 18S | awk '{if($6==$15){print $0}}' | awk '{if(($2<300 || $3<300 || $11<300 || $12<300) && ($2>400 || $3>400 || $11>400 || $12>400)){print $0}}' | awk -v T=$thresh '{if($7>T && $16>T){print $0}}' | wc -l`

else

eS=`cat $file | grep "$sp" | grep 18S | awk '{if($6==$15){print $0}}' | awk -v T=$thresh '{if($7>T && $16>T){print $0}}' | wc -l`

FC=`cat $file | grep "$sp" | grep COI | awk '{if($6==$15){print $0}}' | awk '{if(($2<400 && $3<400 && $11<400 && $12<400)){print $0}}' | awk -v T=$thresh '{if($7>T && $16>T){print $0}}' | wc -l`

Leray=`cat $file | grep "$sp" | grep COI | awk '{if($6==$15){print $0}}' | awk '{if(($2>300 && $3>300 && $11>300 && $12>300)){print $0}}' | awk -v T=$thresh '{if($7>T && $16>T){print $0}}' | wc -l`

Folmer=`cat $file | grep "$sp" | grep COI | awk '{if($6==$15){print $0}}' | awk '{if(($2<300 || $3<300 || $11<300 || $12<300) && ($2>400 || $3>400 || $11>400 || $12>400)){print $0}}' | awk -v T=$thresh '{if($7>T && $16>T){print $0}}' | wc -l`

fi

###Proportions

eSP=`printf %.6f $(echo "$eS / $BothSame" | bc -l)`

FCP=`printf %.6f $(echo "$FC / $BothSame" | bc -l)`

LerayP=`printf %.6f $(echo "$Leray / $BothSame" | bc -l)`

FolmerP=`printf %.6f $(echo "$Folmer / $BothSame" | bc -l)`

echo "$library $sp $eS $FC $Leray $Folmer $eSP $FCP $LerayP $FolmerP" >> $Summary2

fi

previous=$sp

done###end of loop through species

done###end of loop through file

**References for Supporting Information**

Costa, F.O., deWaard, J.R., Boutillier, J. *et al.* (2007). Biological identifications through DNA barcodes: the case of the Crustacea. *Canadian Journal of Fisheries and Aquatic Sciences*, 64, 272-295.

Folmer, O., Black, M., Hoeh, W., Lutz, R & Vrijenhoek R. (1994) DNA primers for amplification of mitochondrial cytochrome *c* oxidase subunit I from diverse metazoan invertebrates. *Molecular Marine Biology and Biotechnology*, 3, 294-299.

Geller, J., Meyer, C., Parker, M. *et al.* (2013). Redesign of PCR primers for mitochondrial cytochrome *c* oxidase subunit I for marine invertebrates and application in all-taxa biotic surveys. *Molecular Ecology Resources*, 13, 851-861.

Leray, M., Yang, J.Y., Meyer, C.P., Mills, S.C., Agudelo, N., Ranwez, V., Boehm, JT. & Machida R.J. (2013). A new versatile primer set targeting a short fragment of the mitochondrial COI region for metabarcoding metazoan diversity: application for characterizing coral reef fish gut contents. *Frontiers in Zoology*, 10, 34.

Lobo, J., Costa, P.M., Teixeira, M.A., Ferreira, M.S., Costa, M.H., Costa, F.O. (2013) Enhanced primers for amplification of DNA barcodes from a broad range of marine metazones. *BMC Ecology*, 13, 34.

Meusnier, I., Singer, G.A.C., Landry, J.F. *et al.* (2008). A universal DNA mini-barcode for biodiversity analysis. *BMC Genomics*, 9, 214.

Meyer, C.P. (2003). Molecular systematic of cowries (Gastropoda: Cypraeidae) and diversification patterns in the tropics. *Biological Journal of the Linnean Society*, 79, 401-459.

Prosser, S., Martinez-Arce, A. & Elias-Gutiérrez, M. (2013). A new set of primers for COI amplification from freshwater microcrustaceans. *Molecular Ecology Resources*, 13, 1151-1155.

Radulovici, A.E., Bernard, S.M., Dufresne, F. (2009) DNA barcoding of marine crustaceans from the Estuary and Gulf of St Lawrence: a regional-scale approach. *Molecular Ecology Resources*, 9, 181-187.

Shokralla, S., Porter, T.M., Gibson, J.F., Dobosz, R., Janzen, D.H., Hallwachs, W., Golding, B., Hajibabaei, M. (2015). Massively parallel multiplex DNA sequencing for specimen identification using an Illumina MiSeq platform. *Scintific Reports*, 5, 9687.

Zhan, A., Hulák, M., Sylvester, F., Huang, X., Adebayo, A.A., Abbott, C.L. … MacIssac, H.J. (2013). High sensitivity of 454 pyrosequencing for detection of rare species in aquatic communities. *Methods in Ecology and Evolution*, 4, 558-565.
